# Safety and tolerability of subcutaneous trastuzumab at home administration, results of the phase IIIb open-label BELIS study in HER2-positive early breast cancer

**DOI:** 10.1007/s10549-020-05604-7

**Published:** 2020-04-02

**Authors:** Hannelore Denys, Corina L. Martinez-Mena, Marc T. Martens, Randal G. D’Hondt, Marie-Pascale L. Graas, Ella Evron, Georgeta Fried, Noa E. Ben-Baruch, Christof Vulsteke, Mona M. Van Steenberghe

**Affiliations:** 1 UZ Gent, C. Heymanslaan 10, 9000 Gent, Belgium; 2 CHU St Pierre, 105, rue aux Laines, 1000 Brussels, Belgium; 3 AZ Turnhout, Rubensstraat 166, 2300 Turnhout, Belgium; 4 AZ Damiaan, Gouwelozestraat 100, 8400 Ostend, Belgium; 5 CHC - Clinique Saint-Joseph, Rue de Hesbaye 75, 4000 Liège, Belgium; 6 Assaf Harofeh Medical Center, 70300 Rishon-Le-Zion, Israel; 7 Rambam Medical Center, HaAliya HaShniya St 8, PO Box 9602, 31096 Haifa, Israel; 8 Kaplan Medical Center, Derekh Pasternak, Bilu Junction, PO Box 1, 76100 Rehovot, Israel; 9 Integrated Cancer Center Ghent, AZ Maria Middelares, Buitenring Sint-Denijs 30, 9000 Gent, Belgium; 10 Center for Oncological Research (CORE), Antwerp University, Wilrijkstraat 10, 2650 Edegem, Belgium; 11 Roche nv/sa, Dantestraat 75, 1070 Brussels, Belgium

**Keywords:** Safety, Tolerability, Subcutaneous trastuzumab, HER2-positive early breast cancer, At home administration

## Abstract

**Purpose:**

The subcutaneous (SC) administration of trastuzumab is highly preferred by patients. At home, administration of trastuzumab SC might further improve patient benefit. The aims of the BELIS study are to evaluate the safety and tolerability of trastuzumab SC when administered at home by a healthcare professional (HCP) and to evaluate patient-reported outcomes for treatment experience of at home cancer therapy.

**Methods:**

This open-label phase IIIb study enrolled HER2-positive early breast cancer patients in Belgium and Israel who completed the first six cycles of trastuzumab IV (neo)adjuvant therapy. The study consisted of three consecutive treatment periods: three cycles of trastuzumab IV and SC each at the hospital and six cycles of trastuzumab SC at home.

**Results:**

Between November 2013 and December 2014, 23 centres enrolled 102 patients in the intent-to-treat population of which 101 patients entered the safety population. No new safety signals were detected with as expected, more mild administration site events with trastuzumab SC when compared to IV treatment. All patients agreed that they had benefit from at home administration to a large (18/81; 22%) or very large (63/81; 78%) extent. All HCPs (21/21) agreed that SC is the quickest method from start of preparation to finish of administration and that less resource use is needed.

**Conclusion:**

The results of the BELIS study support that trastuzumab SC can be safely administered at home by a HCP and all patients considered this setting as beneficial. HCPs consider the SC formulation as the quickest method to administer trastuzumab.

**Trial registration:**

EudraCT Identifier: 2013-000123-13. ClinicalTrials.gov Identifier: NCT01926886.

**Electronic supplementary material:**

The online version of this article (10.1007/s10549-020-05604-7) contains supplementary material, which is available to authorized users.

## Introduction

Among females worldwide, breast cancer (BC) is the most commonly diagnosed cancer (24% of the total new cancer cases) and the leading cause of cancer death (15% of the total cancer deaths) [1]. The large majority of newly diagnosed BCs are early BC (eBC) and 15% to 20% of patients have tumours that exhibit overexpression of the Human Epidermal Growth Factor Receptor 2 (HER2) [2, 3]. These tumours are associated with poorer prognosis compared with tumours that do not overexpress HER2 [4, 5].

It has been demonstrated in large randomized trials that adjuvant trastuzumab for HER2-positive eBC improves disease-free survival (DFS) and overall survival [6–8]. One year adjuvant treatment with trastuzumab is well established in HER2-positive eBC. The subcutaneous (SC) administration of trastuzumab over about 5 min has a pharmacokinetic profile and efficacy non-inferior to intravenous (IV) administration with a similar safety profile [9–12]. The SC administration of trastuzumab is highly preferred by the patients and this is important for treatments over prolonged periods of time [13, 14]. At home administration of trastuzumab SC might further improve the patient satisfaction during treatment compared to administration in the hospital.

An important reduction of patient chair time and active healthcare professional (HCP) time with trastuzumab SC was demonstrated in the time and motion study within the PrefHer study [15]. Shorter patient chair time could increase centre capacity and reduce waiting lists. HCP time savings could allow for more time dedicated to other patient care activities.

The BELIS study is to our knowledge the first prospective study to assess the overall safety and tolerability of trastuzumab SC when administered by a HCP at the home of the patient. Patient-reported outcomes for experience and satisfaction with the treatment provided at home and in the hospital are important aspects of the study.

## Methods

### Study design, participants and procedures

**Fig. 1:**
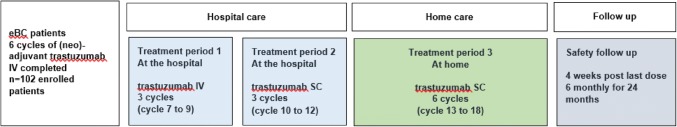
Study design

For this prospective, phase IIIb, open-label, multinational, multicentre study, centres in Belgium and Israel enrolled patients with HER2-positive eBC. Eligible patients with an Eastern Cooperative Oncology Group (ECOG) performance status of 0–1, a left ventricular ejection fraction (LVEF) of at least 50%, had completed the first six cycles of (neo)adjuvant trastuzumab treatment were enrolled in the intent-to-treat population (ITT). All enrolled patients who received at least one dose of the study medication (trastuzumab SC or trastuzumab IV) were included in the safety (SA) population. Patients were treated with surgery, chemotherapy, radiotherapy or hormonotherapy according to local standards. The study included three consecutive trastuzumab treatment periods. In the first treatment period, patients received in the hospital three cycles of the recommended maintenance dose of 6 mg/kg trastuzumab IV at 3-weekly intervals. In the second treatment period, patients received in the hospital three cycles of trastuzumab SC 600 mg fixed dose at 3-weekly intervals. In the third treatment period, patients received at home six cycles of 600 mg trastuzumab SC fixed dose, administered by a trained HCP at 3-weekly intervals (Fig. 1).

The safety follow-up visit, 4 weeks after the end of treatment, was followed by a long-term follow-up period of two years with four visits every 6 months.

### Outcomes

Primary outcome of the study was overall safety and tolerability of trastuzumab SC when administered at home by a trained HCP. The adverse events (AE) and serious adverse events (SAE) were continuously monitored and documented during the entire study, at each 3-weekly treatment visit and during post-treatment follow-up. Intensity of the recorded AEs were graded according to the National Cancer Institute Common Terminology Criteria for Adverse Events (NCI CTCAE) version 4.0. Adverse events of special interest, such as congestive heart failure (CHF), LVEF decrease over time or other cardiac events were collected. Secondary objectives were patient experience with trastuzumab IV and SC, patient-reported quality of care, patient-reported symptoms, HCP overall satisfaction and perceived time savings and efficacy assessed through disease-free survival (DFS).

In order to evaluate the patient experience with trastuzumab IV and SC administered in the hospital, HCPs interviewed the patients before the start of the third dose of trastuzumab at the hospital and completed the patient experience (PEX)-P1questionnaire which contains 25 items. At the safety follow-up visit, patients were interviewed again by the HCP to complete the 6-item PEX-P2 questionnaire to evaluate their experience with trastuzumab SC administered at home. To evaluate overall perception of the quality of care in the hospital, patients completed the patient satisfaction (PS)Q1 questionnaire before receiving the first dose of trastuzumab SC at home. PSQ1 is a quality of care questionnaire containing 28 questions categorised on the opinion of patients about the clinicians, the hospital staff and other questions. To evaluate the experience with at home treatment, patients completed the PSQ2 questionnaire before receiving the fifth cycle of trastuzumab SC at home. PSQ2 is a quality of care questionnaire similar to PSQ1 containing 26 questions. Also patients rated interference with daily life and the severity of 13 symptoms (pain, fatigue, nausea, vomiting, disturbed sleep, distress, shortness of breath, memory difficulties, lack of appetite, drowsiness, dry mouth, sadness, numbness or tingling) on a ten-point scale of the MD Anderson Symptom Inventory (MDASI) questionnaire at cycles 7, 10, 13 and 16. Health care professionals completed the Healthcare Professionals’ Experience (HCPEX) questionnaire to evaluate overall satisfaction and perceived time savings with trastuzumab SC, after at least 3 patients had completed the hospital SC and IV treatment periods.

Disease-free survival, defined as time from the date of first study drug administration to the date of local, regional or distant recurrence, contralateral BC or death due to any cause was monitored. Diagnosis of BC relapse was made based on routine clinical practice.

### Statistical methods

Descriptive statistical methods were used to analyse and report the results of this single-arm study. It was planned to enrol 100 patients. This sample size was not based on a formal calculation but mainly driven by feasibility and a reasonable width of a 95% confidence interval (CI) for the rate of AEs and SAEs. Based on an expected rate of AEs of 97% [9] and 100 patients, a 95% CI of 91% to 99% would be obtained. For an expected rate of SAEs of 21% [9] and 100 patients, a 95% CI ranging from 13 to 29% would be obtained (CIs calculated using Clopper–Pearson methodology).

The protocol with a full description of the methods and the questionnaires used, is available in Online Resource 1.

## Results

**Fig. 2:**
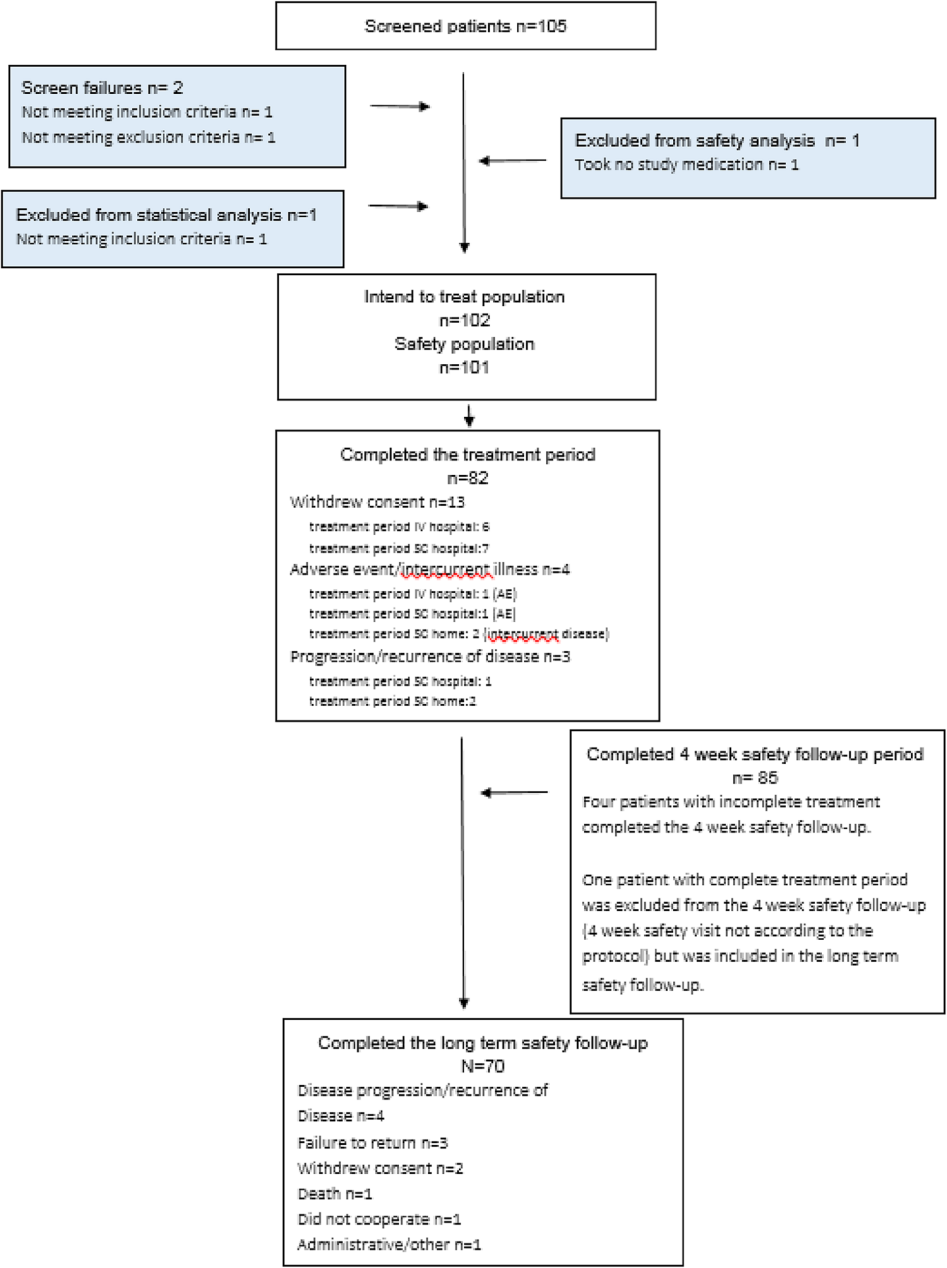
Patient disposition

Between November 2013 and December 2014, 23 centres, 13 in Belgium and 10 in Israel, screened 105 patients and enrolled 102 patients in the intent-to-treat (ITT) population, 51 patients in Belgium and 51 patients in Israel. One ITT patient did not receive trastuzumab and was excluded from the safety (SA) population. Twenty patients did not complete the study period. The most important reason was withdrawal of consent during the hospital IV and SC treatment periods (Fig. 2).

In the SA population, overall mean age at screening was 54 years (range 24–80 years). All patients were female except for one male patient. Medical and breast cancer disease history were recorded: 73.3% of patients in the SA population had an oestrogen receptor positive tumour and 50.5% of patients a progesterone receptor positive tumour (Table 1).

**Table 1:** Population characteristics

Characteristic	Safety population *N* = 101
*Demographic characteristic*	
Mean (range) age at screening (years)	54 (24–80)
Gender, *n* (%)	
Female	100 (99.0%)
Male	1 (1.0%)
*History of breast cancer (primary tumour)*	
Primary tumour size, *n* (%)	
< 2 cm	47 (46.5%)
2 cm–5 cm	43 (42.6%)
> 5 cm	11 (10.9%)
Breast cancer subtype^a^, *n* (%)	
Ductal	90 (89.1%)
Lobular	3 (3.0%)
Both ductal and other	4 (4.0%)
Other	4 (4.0%)
Breast cancer type, *n* (%)	
Operable	93 (92.1%)
Locally advanced inoperable	6 (5.9%)
Inflammatory	2 (2.0%)
Nuclear grade, *n* (%)	
Grade 1	1 (1.0%)
Grade 2	34 (33.7%)
Grade 3	46 (45.5%)
Unknown/missing	20 (19.8%)
Oestrogen receptor positive, *n* (%)	74 (73.3%)
Progesterone receptor positive, *n* (%)	51 (50.5%)
*Primary surgery for breast cancer*	
Lymph node status, *n* (%)	
Node negative	59 (58.4%)
Node positive	40 (39.6%)
Unknown/missing	2 (2.0%)

^a^A patient can have several breast cancer subtypes

### Safety results during the treatment period and 4 weeks safety follow-up

A total of 551 AEs were reported in 91/101 (90.1%–95% CI [82.54–95.15]) patients. Of these, 194 events in 52/101 (51.5%–95% CI [41.33–61.55]) patients were considered to be related to trastuzumab. During the 3 cycles of trastuzumab IV at the hospital, 7/101 (6.9%–95% CI [2.83–13.76]) patients experienced a total of 7 trastuzumab-related AEs. During the 3 cycles of trastuzumab SC at the hospital, 30/94 (31.9%–95% CI [22.67–42.33]) patients experienced a total of 49 trastuzumab-related AEs. During the 6 cycles with trastuzumab SC at home, 40/86 (46.5%–95% CI [35.68–57.59]) patients experienced a total of 139 trastuzumab-related AEs. It is to be noted that the number of cycles during the at home SC treatment is twofold higher (6 cycles) compared to the number of cycles during the hospital IV or SC treatment periods (3 cycles). The related AEs observed in at least 5% of the patients during at least 1 treatment period were mostly administration site conditions and fatigue with trastuzumab SC (Table 2). Most of the related and unrelated AEs were graded CTC 1 or 2 (Table 3). Data of the administrations site conditions and fatigue at patient level are available in Online Resource 2.

**Table 2:** Patients with AEs related to study drug occurring in at least 5% of patients during at least 1 treatment period

Trastuzumab-related adverse events	Trastuzumab IV At the hospital 3 cycles *N* = 101	Trastuzumab SC At the hospital 3 cycles *N* = 94	Trastuzumab SC At home 6 cycles *N* = 86
Any AE related to study drug, *n* (%)	7 (6.9%)	30 (31.9%)	40 (46.5%)
General disorders and administration site conditions, *n* (%)	2 (2.0%)	20 (21.3%)	33 (38.4%)
Injection site swelling, *n* (%)	0 (0%)	2 (2.1%)	13 (15.1%)
Injection site pain, *n* (%)	0 (0%)	8 (8.5%)	7 (8.1%)
Injection site erythema, *n* (%)	0 (0%)	4 (4.3%)	12 (14.0%)
Fatigue, *n* (%)	1 (1.0%)	3 (3.2%)	5 (5.8%)
Musculoskeletal and connective tissue disorders, *n* (%)	0 (0%)	8 (8.5%)	5 (5.8%)
Myalgia, *n* (%)	0 (0%)	4 (4.3%)	2 (2.3%)

**Table 3:** Patients with AEs by worst intensity

Adverse events	Trastuzumab IV hospital 3 cycles *N* = 101	Trastuzumab SC hospital 3 cycles *N* = 94	Trastuzumab SC at home 6 cycles *N* = 86
At least one AE, *n* (%)	57 (56.4%)	57 (60.6%)	73 (84.9%)
Grade unknown	1 (1.0%)	0	1 (1.2%)
CTC grade 1	45 (44.6%)	46 (48.9%)	66 (76.7%)
CTC grade 2	23 (22.8%)	20 (21.3%)	35 (40.7%)
CTC grade 3	1 (1.0%)	4 (4.3%)	5 (5.8%)
CTC grade 4	1(1.0%)	0	0
CTC grade 5	0	0	0
Injection site swelling, *n* (%)			
Grade unknown	0	0	0
CTC grade 1	0	2 (2.1%)	12 (14%)
CTC grade 2	0	0	1 (1.2%)
CTC grade 3	0	0	0
CTC grade 4	0	0	0
Injection site pain, *n* (%)			
CTC grade unknown	0	0	0
CTC grade 1	0	6 (6.4%)	6 (7.0%)
CTC grade 2	0	2 (2.1%)	2 (2.3%)
CTC grade 3	0	1 (1.1%)	0
CTC grade 4	0	0	0
Injection site erythema, *n* (%)			
CTC grade unknown	0	0	0
CTC grade 1	0	5 (5.3%)	12 (14.0%)
CTC grade 2	0	0	0
CTC grade 3	0	0	0
CTC grade 4	0	0	0
Fatigue, *n* (%)			
CTC grade unknown	0	0	0
CTC grade 1	7 (6.9%)	5 (5.3%)	13 (15.1%)
CTC grade 2	3 (3.0%)	0	9 (10.5%)
CTC grade 3	0	1 (1.1%)	0
CTC grade 4	0	0	0
Myalgia, *n* (%)			
CTC grade unknown	0	0	0
CTC grade 1	0	3 (3.2%)	3 (3.5%))
CTC grade 2	1 (1.0%)	1 (1.1%)	1 (1.2%)
CTC grade 3	0	0	0
CTC grade 4	0	0	0
Ejection fraction decrease, *n* (%)			
CTC grade unknown	0	0	0
CTC grade 1	0	0	0
CTC grade 2	1 (1.0%)	1 (1.1%)	0
CTC grade 3	0	1 (1.1%)	2 (2.3%)
CTC grade 4	0	0	0

Overall, four cardiac AEs were reported in four patients (atrial fibrillation, sinus tachycardia, hypertension and palpitation).

A total of eight SAEs were reported in eight patients. Of these, five were considered to be related to trastuzumab, all five were a decrease of LVEF. Two cases of LVEF decrease during the hospital treatment period led to withdrawal of the study treatment. Two cases of LVEF decrease occurred in the at home treatment period. During the at home treatment period, there were no AEs that led to discontinuation of study medication.

### Safety results during the long-term follow-up

During the long-term safety follow-up period, a total of 35 non-emergent AEs were reported in 24 patients. Non-emergent AEs are defined as events occurring at least 35 days after the last study drug administration. Three cardiac non-emergent AEs were reported in 3 patients during the long-term follow-up. One decrease in LVEF was a serious cardiac non-emergent AE considered related to trastuzumab. Left ventricular dysfunction and atrial tachycardia were non-serious non-emergent cardiac events. Two deaths were reported, both assessed unrelated to trastuzumab. There were no reports of CHF events during the entire conduct of the study.

### The patient experience questionnaires PEX-P1, PEX-P2 and MDASI

**Fig. 3:**
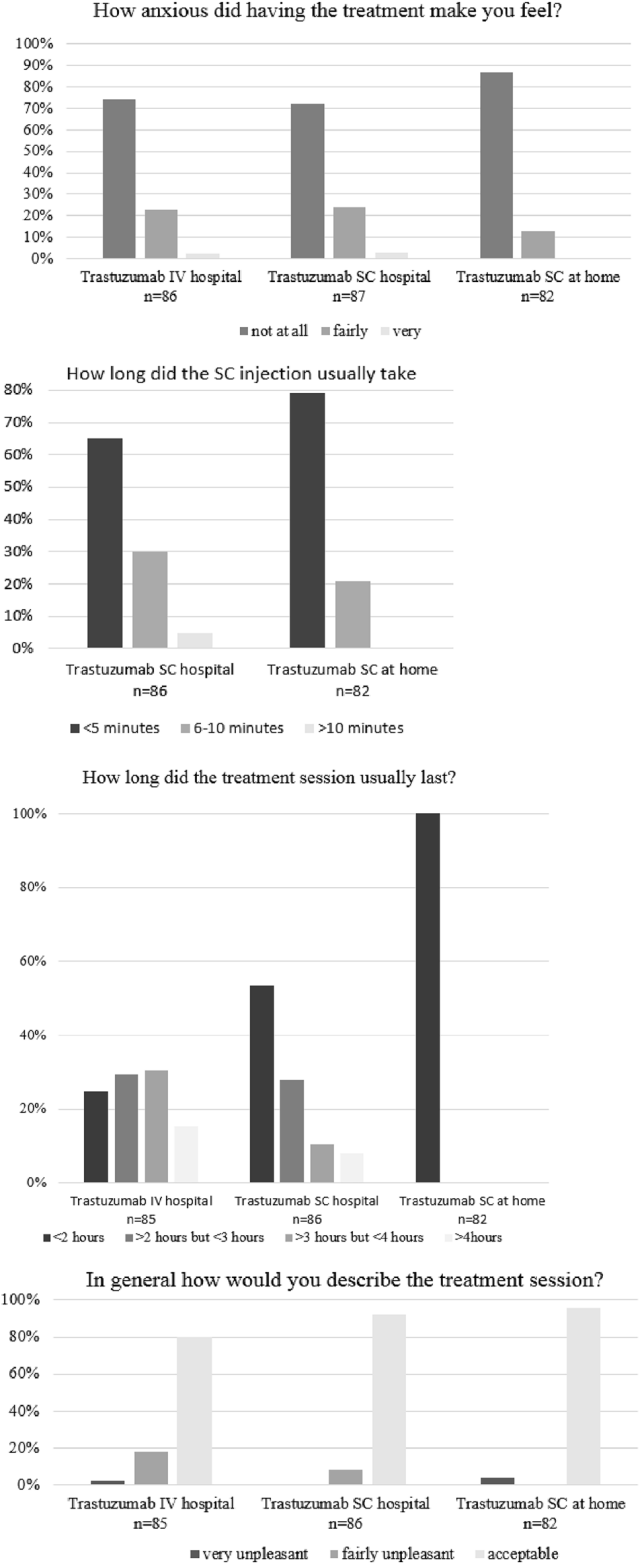
Treatment experience with trastuzumab at the hospital (IV and SC) and at home (SC)

HCPs interviewed the patients about their experience with IV and SC trastuzumab administration in the hospital and trastuzumab SC administration at home. Travelling to the hospital for treatment was a problem for 18/87 (20.7%) of patients (Belgium 6.3%–Israel 38.5%), mainly due to long travelling time, unable to travel alone, cost and difficulties to travel. The SC injection took 10 min or less for almost all patients. The complete treatment session was reduced to 2 h for all at home treated patients 82/82 (100%), the majority 71/82 (86.6%) of patients was not at all anxious during the at home treatment and 79/82 (96.3%) patients indicated that the treatment session at home was acceptable (Fig. 3).

*Results of the patient satisfaction questionnaires PSQ1 and PSQ2*, completed by the patients, indicated that patients had confidence and felt supported by the doctors and nurses in the hospital and in the at home setting. Overall, patients were satisfied to a large or very large extent with the help and treatment they received in the hospital 83/84 (98.8%) and at home 81/81 (100%). However, 24/82 (29.3%) patients indicated that they had to wait quite long or very long before being admitted for service at the institution (Table 4). All patients 81/81 (100%) indicated that home care was beneficial to a large 18/81 (22.2%) or very large 63/81 (77.8%) extent and this benefit was very important or of utmost importance for 62/82 (75.6%) patients.

*On the MDASI scale*, all experienced symptoms were rated as relatively mild with all mean scores remaining lower than or equal to 3.76 on the 10-point scale at all time points. Most severe symptoms were fatigue, disturbed sleep and numbness or tingling. The interference of those symptoms with the daily life and functioning also remained equal or lower to the mean score of 3.34 on the 10-point scale at all time points.

**Table 4:** Waiting time before being admitted for service in the hospital

Did you have to wait long before you were admitted for service in the hospital	*N* = 82
No *n* (%)	24 (29.3%)
Yes but not long *n* (%)	34 (41.5%)
Yes quite long *n* (%)	20 (24.4%)
Yes very long *n* (%)	4 (4.9%)

### HCP experience with trastuzumab IV and SC

Results of the HCPEX questionnaire for trastuzumab treatment showed that all HCPs found it very easy or fairly easy to administer trastuzumab via the IV or the SC route. However, 11/21 (52.4%) of responding HCPs estimated that the duration of the trastuzumab IV session lasted more than 2 h but less than 3 h and 5/21 (23.8%) of responding HCPs longer than 3 h. All HCPs (21/21; 100%) agreed that SC would be the quickest method from start of preparation to finish of administration and that less resource use for preparation and administration was needed.

### Efficacy results

Up until the safety follow-up visit (4 weeks after end of treatment), 3 patients had a recurrence of the disease and during the 2-year long-term follow-up 9 patients had a recurrence of the disease. The median time to event could not be estimated because only 12 patients out of 101 (11.9%) had a recurrence or progression or died.

## Discussion

The BELIS study is the first prospective study to assess safety and tolerability of trastuzumab SC administered at the home of the patient by a HCP. During the 6 cycles of trastuzumab SC at home, 40/86 (46.5%) patients experienced a total of 139 trastuzumab-related AEs. As expected, most of these related events were mild administration site conditions such as injection site swelling, injection site pain or injection site erythema and fatigue. During the 3 cycles of trastuzumab IV at the hospital, 7/101 (6.9%) patients experienced a related AE. No administration site conditions were reported during the IV treatment period. During the 3 cycles of trastuzumab SC at the hospital the observed AEs were again mostly mild grade 1 administration site conditions. There was a larger proportion of patients experiencing trastuzumab-related events during the at home treatment period compared to the hospital treatment periods. It should be noted that the at home period contained 6 treatment cycles compared to 3 treatment cycles during the hospital IV and SC periods. Also, at home the HCP was responsible for only one patient. This might have resulted in a more complete reporting of the mostly mild events in the at home setting compared to reporting in a busy hospital setting.

During the treatment period, five trastuzumab-related SAEs were reported: five cases of LVEF decrease of which two during the at home treatment period. During the long-term safety follow-up, one case of LVEF decrease, classified as a SAE related to trastuzumab, was reported. There were no cases of CHF during the entire study period. During the conduct of the BELIS study, no new safety signals were detected for trastuzumab SC. The observed safety profile of trastuzumab SC at home and in the hospital was comparable to the safety results of two randomized studies and the single-arm study SafeHer [9–12, 16].

Observed differences in AE rates between trastuzumab SC and trastuzumab IV in the large PrefHer study were driven by injection site reactions. Adverse events rates were similar between trastuzumab SC (275/479 [57.4%]) and trastuzumab IV (58/478 [54.0%]) when injection site reactions were excluded [10].

An important aspect of the study was the experience and satisfaction of the patients with trastuzumab SC treatment at the home of the patient. There is an increasing interest in the administration of cancer treatment outside the hospital based on patient preferences, capacity issues in oncology day care hospitals facing an increasing number of patients and treatments, and the increased use of oral chemotherapy [17]. In a feasibility study of home-based chemotherapy, patient benefits of home care included increased comfort and freedom [18]. All the patients in the BELIS study who completed the questionnaire concerning the quality of care in the hospital and at home indicated that being treated at home with trastuzumab SC was beneficial to a large or very large extent. The duration of the complete treatment session was reduced to 2 h for all at home treated patients and difficulties to travel to the hospital can be avoided. We observed surprisingly long treatment sessions in the hospital for the trastuzumab SC treatment as well as for the trastuzumab IV sessions. The underlying reasons for these long treatment sessions are not clear but possibly due to logistic problems in the organisation of the hospital.

Results of the BELIS study confirm the benefit for patients and HCPs of SC administration of trastuzumab compared to IV administration observed in previous studies [13, 14, 19]. Also, the time savings observed with SC treatment could lead to a capacity gain of the day care facilities and patients spending less time at the hospital, which may improve their quality of life. The time and motion study within the PrefHer study showed that across different countries and centres, the trastuzumab SC administration led to an important reduction of patient chair time and of active HCP time [15]. In a single-centre, Belgian, time, motion and cost assessment study, it was observed that the total time patients spent in the day care centre was 71% shorter with SC administration of trastuzumab. From a HCP perspective, the total preparation and administration time for IV trastuzumab was 4.07 times longer than the total time required for SC administration. Also, IV administration was more costly, mainly due to more HCP time, more consumable supplies and drug wastage [20].

The BELIS study is to our knowledge the first prospective study evaluating the safety and patient experience of trastuzumab SC administered by a HCP at the home of the patient. During treatment in the hospital or at home, the observed safety profile of trastuzumab SC was consistent with the known safety profile of trastuzumab. As expected, more administration site reactions of mild intensity were reported with the SC administration. All patients agreed that they had benefit from at home administration to a large or very large extent. The SC administration is considered by the HCPs as the quickest way to administer trastuzumab. The results of the BELIS study support that trastuzumab SC can be safely and comfortably administered by a HCP at the home of the patient and this setting is beneficial for HER2, eBC patients undergoing treatment.

## Electronic supplementary material

Below is the link to the electronic supplementary material.

Supplementary file1 (PDF 605 kb)

Supplementary file2 (PDF 55 kb)
