# Supplementary material for: Safety and tolerability of subcutaneous trastuzumab at home administration, results of the phase IIIb open-label BELIS study in HER2-positive early breast cancer
Source: Breast Cancer Res Treat. 2020 Apr 2;181(1):97–105. doi: 10.1007/s10549-020-05604-7 (PMC7182624; doi:10.1007/s10549-020-05604-7)
Supplement: Supplementary file 1 — Supplementary file1 (PDF 605 kb) [file 10549_2020_5604_MOESM1_ESM.pdf]

PROTOCOL

**TITLE:**	**A SINGLE ARM MULTI-CENTER STUDY INVESTIGATING THE AT HOME ADMINISTRATION OF TRASTUZUMAB SUBCUTANEOUS VIAL FOR THE TREATMENT OF PATIENTS WITH HER2-POSITIVE EARLY BREAST CANCER.**
**PROTOCOL NUMBER:**	ML28794
**VERSION NUMBER:**	3.0
**EUDRACT NUMBER:**	2013-000123-13
**IND NUMBER:**	N.A.
**TEST PRODUCT:**	Trastuzumab SC (RO 45-2317)
**MEDICAL MONITOR:**	Marianne Heijndijk
**SPONSOR:**	F. Hoffmann-La Roche Ltd

FINAL PROTOCOL APPROVAL

Dr. Hans Warrinnier

Medical Director

Date: **Signature:**

Dr. Christine Lenaerts

Qualified person Pharmacovigilance

Date: **Signature:**

CONFIDENTIAL STATEMENT

The information contained in this document, especially any unpublished data, is the property of F. Hoffmann-La Roche Ltd. (or under its control), and therefore provided to you in confidence as an investigator, potential investigator, or consultant, for review by you, your staff, and an applicable Ethics Committee or Institutional Review Board. It is understood that this information will not be disclosed to others without written authorization from Roche except to the extent necessary to obtain informed consent from those persons to whom the drug may be administered.

TABLE OF CONTENTS

PROTOCOL ACCEPTANCE FORM 8

PROTOCOL SYNOPSIS 9

1. BACKGROUND 20

1.1 Background on Breast Cancer – Treatment Options for Early Breast Cancer 20

1.2 Background on Study Treatments 20

1.2.1 Human Epidermal Growth Factor Receptor 2 (HER2) 20

1.2.2 Trastuzumab (rhuMAb HER2, Herceptin®) 21

1.2.2.1 Trastuzumab IV 22

1.2.2.2 Pharmacokinetics of Trastuzumab IV 22

1.2.2.3 Efficacy of Trastuzumab IV in Early Breast Cancer (Adjuvant Setting) 23

1.2.2.4 Safety of Trastuzumab IV 24

1.2.2.5 Trastuzumab SC 26

1.2.2.6 Non-clinical Studies with Trastuzumab SC 28

1.2.2.7 Clinical Studies with Trastuzumab SC 28

1.3 Study Rationale and Benefit–Risk Assessment 32

1.3.1 Rationale for Test Product Dosage 32

1.3.2 Rationale for Patients with eBC 33

1.3.3 Benefits of SC Administration of Trastuzumab 34

2. OBJECTIVES 35

2.1 Primary Objective 35

2.2 Secondary Objectives 36

3. STUDY DESIGN 36

3.1 Description of Study 36

3.1.1 Patient population 37

3.1.2 Steering Committee 37

3.2 Length of Study 37

3.3 Safety Follow-up 37

3.4 End of Study 37
3.5 Overall Number of Patients 37
3.6 Patient Enrolment 37
3.7 Countries/Centers 37
3.8 Rationale for Study Design 38
3.8.1 Rationale for Pooling Strategy 38
3.8.2 Rationale for Test Product Regimen 39
3.9 Outcome Measures 40
3.9.1 Safety Outcome Measures 40
3.9.2 Patient-Reported Outcome Measures 40
3.9.3 HCP-REPORTED OUTCOME MEASURES 41
3.9.4 OTHER 41

4. MATERIALS AND METHODS 41
4.1 Patients 41
4.2 Inclusion Criteria 42
4.3 Exclusion Criteria 42
4.4 Study Treatment 44
4.4.1 Formulation, Packaging, and Handling 44
4.4.1.1 Trastuzumab IV 44
4.4.1.2 Trastuzumab SC 45
4.4.2 Dosage, Administration, and Compliance 45
4.4.3 Investigational Medicinal Product Accountability 46
4.4.3.1 Assessment of Compliance 47
4.4.3.2 Destruction of the IMPs 47
4.4.4 Post-Trial Access to Trastuzumab SC 48
4.5 Concomitant Therapy 48
4.5.1 Permitted Therapy 48
4.5.2 Prohibited Therapy 48
4.6 Study Assessments 49
4.6.1 Description of Study Assessments 49
4.6.1.1 Mandatory Procedure for Enrolment 49
4.6.1.2 Clinical Assessments During Treatment Period 50

4.6.1.3 Clinical Assessments at the Safety Follow-up visit 54
4.6.1.4 Clinical Assessments in Follow-up visits 54
4.6.1.5 Follow-Up Assessments 54
4.6.1.6 Assessments at Unplanned Visits 54
4.7 Patient, Study, and Site Discontinuation 55
4.7.1 Patient Discontinuation 55
4.7.1.1 Discontinuation from Study Drug 55
4.7.1.2 Withdrawal from Study 55
4.7.2 Study and Site Discontinuation 55
5. ASSESSMENT OF SAFETY 56
5.1 Safety Plan 56
5.1.1 General Safety Assessments 56
5.1.2 Cardiac Safety Assessments 57
5.1.2.1 LVEF Assessment 57
5.1.3 Management of Specific AEs 57
5.1.3.1 Infusion-Associated Symptoms and Allergic Reactions 58
5.1.4 Management of Specific Adverse Events 58
5.1.4.1 Dose Modifications, Interruptions and Delays for Trastuzumab 58
5.2 Safety Parameters and Definitions 59
5.2.1 Adverse Events 60
5.2.2 Serious Adverse Events (Immediately Reportable to the Sponsor) 60
5.2.3 Pregnancy and Contraception 61
5.3 Methods and Timing for Capturing and Assessing Safety Parameters 61
5.3.1 Adverse Event Reporting Period 62
5.3.2 Table 3 Eliciting Adverse Event Information 62
5.3.3 Assessment of Severity of Adverse Events 62
5.3.4 Assessment of Causality of Adverse Events 63
5.3.5 Procedures for Recording Adverse Events 64
5.3.5.1 Diagnosis versus Signs and Symptoms 64

5.3.5.2 Adverse Events Occurring Secondary to Other Events 64
5.3.5.3 Persistent or Recurrent Adverse Events 65
5.3.5.4 Abnormal Laboratory Values 65
5.3.5.5 Abnormal Vital Sign Values 66
5.3.5.6 Abnormal Liver Function Tests 66
5.3.5.7 Deaths 67
5.3.5.8 Preexisting Medical Conditions 67
Worsening of the Underlying Condition 67
5.3.5.9 Hospitalization or Prolonged Hospitalization 67
5.3.5.10 Overdoses 68
5.3.5.11 Patient-Reported Outcome Data 68
5.4 Immediate Reporting Requirements from Investigator to Sponsor 68
5.4.1 Emergency Medical Contacts 69
5.4.2 Reporting Requirements for Serious Adverse Events 69
5.4.3 Reporting Requirements for Pregnancies 70
5.4.3.1 Pregnancies in Female Patients 70
5.4.3.2 Pregnancies in Female Partners of Male Patients 70
5.4.3.3 Abortions 71
5.4.3.4 Congenital Anomalies/Birth Defects 71
5.4.4 Reporting Requirements for Cardiac Events related to CHF 71
5.5 Follow-Up of Patients after Adverse Events 71
5.5.1 Investigator Follow-Up 71
5.5.2 Sponsor Follow-Up 72
5.6 Post-Study Adverse Events 72
5.7 Expedited Reporting to Health Authorities, Investigators, Institutional Review Boards, and Ethics Committees 73
6. STATISTICAL CONSIDERATIONS AND ANALYSIS PLAN 73
6.1 Determination of Sample Size 74
6.2 Summaries of Conduct of Study 74

6.3 Summaries of BASELINE CHARACTERISTICS 74
6.4 Safety Analyses 74
6.5 Patient Reported Outcome Analysis 76
7. DATA COLLECTION AND MANAGEMENT 76
7.1 Data Quality Assurance 76
7.2 Electronic Case Report Forms 77
7.3 Patient-Reported Outcome Data 78
7.4 Source Data Documentation 78
7.5 Use of Computerized Systems 79
7.6 Retention of Records 79
8. ETHICAL CONSIDERATIONS 79
8.1 Compliance with Laws and Regulations 79
8.2 Informed Consent 79
8.3 Institutional Review Board or Ethics Committee 80
8.4 Confidentiality 80
8.5 Financial Disclosure 81
9. STUDY DOCUMENTATION, MONITORING, AND ADMINISTRATION 81
9.1 Study Documentation 81
9.2 Site Inspections 81
9.3 Administrative Structure 81
9.4 Publication of Data and Protection of Trade Secrets 81
9.5 Protocol Amendments 82
10. REFERENCES 83

LIST OF APPENDICES

1. Table of Assessments
2. LVEF Algorithm
3. NYHA Classification
4. MDASI
5. HCPEX
6. PSQ1 & PSQ2
7. PEX-P1 & PEX-P2

PROTOCOL ACCEPTANCE FORM

**TITLE:**	**A SINGLE ARM MULTI-CENTER STUDY INVESTIGATING THE AT HOME ADMINISTRATION OF TRASTUZUMAB SUBCUTANEOUS VIAL FOR THE TREATMENT OF PATIENTS WITH HER2-POSITIVE EARLY BREAST CANCER.**
**PROTOCOL NUMBER:**	ML28794
**VERSION NUMBER:**	3.0
**EUDRACT NUMBER:**	2013-000123-13
**TEST PRODUCT:**	Trastuzumab SC (RO 45-2317)
**MEDICAL MONITOR:**	Marianne Heijndijk
**SPONSOR:**	N.V. Roche S.A.

I agree to conduct the study in accordance with the current protocol.

____

Principal Investigator's Name (print)

______________________________________ ________________

Principal Investigator's Signature Date

Please return a copy of the form to the contact provided below. Please retain the original for your study files.

{Name}
{Address}

PROTOCOL SYNOPSIS

**TITLE:**	**A SINGLE ARM MULTI-CENTER STUDY INVESTIGATING THE AT HOME ADMINISTRATION OF TRASTUZUMAB SUBCUTANEOUS VIAL FOR THE TREATMENT OF PATIENTS WITH HER2-POSITIVE EARLY BREAST CANCER.**
**PROTOCOL NUMBER:**	ML28794
**EUDRACT NUMBER:**	2013-000123-13
**TEST PRODUCT:**	Trastuzumab SC (RO 45-2317)
**PHASE:**	IIIb
**INDICATION:**	HER2-positive early breast cancer.
**SPONSOR:**	N.V. Roche S.A.

Objectives

Primary Objective

The primary objective of this study is to assess the overall safety and tolerability of subcutaneous (SC) trastuzumab with assisted administration using a conventional syringe and needle (vial formulation, hereafter referred to as trastuzumab SC vial) when administered at home for the treatment of patients with HER2-positive (HER2+) early breast cancer (eBC).

Secondary Objectives

To describe:

- The evolution of individual patient experience across treatment periods:
  - Patient experience with trastuzumab IV administered in the hospital.
  - Patient experience with trastuzumab SC vial administered in the hospital.
  - Patient experience with trastuzumab SC vial administered at home.
- Patient reporting of symptoms.
- Patient reporting on the quality of care provided in the hospital and at home.
- HCP overall satisfaction and perceived time savings with trastuzumab SC vial formulation administered in the hospital.
- Disease-free survival (DFS).

Study Design

Description of Study

Single arm multicenter study planned to enroll 100 patients with HER2+ eBC as part of a global prospective, non-randomized, open label phase IIIb study.

Patients with HER2+ eBC who completed the first 6 cycles of trastuzumab IV as part of the (neo)adjuvant treatment can be included to continue to receive 12 cycles of trastuzumab to complete a total of 18 cycles of trastuzumab.

The study will assess the safety and tolerability of trastuzumab SC vial when administered at home.

In total, patients will receive 12 cycles of trastuzumab in the study to complete a total of 18 cycles trastuzumab.

The 12 cycles of trastuzumab will be administered according to following schedule:

- Trastuzumab IV will be administered in the hospital by a HCP for the first 3 cycles (Trastuzumab cycle 7 to 9 defined as treatment period 1)
- Trastuzumab SC vial will be administered in the hospital by a HCP for the next 3 cycles (Trastuzumab cycle 10 to 12 defined as treatment period 2)
- Trastuzumab SC vial will be administered at home by a HCP for the following 6 cycles (Trastuzumab cycles 13 to 18 defined as treatment period 3)

Appendix 1, schedule of assessments.

Number of Patients

This study will include 100 patients in total.

Target Population

Patients must meet the following criteria for study entry:

Inclusion Criteria:

1. Female and male patients aged ≥ 18 years
2. Signed informed consent prior to any study specific procedure
3. Able and willing to comply with protocol
4. Eastern Cooperative Oncology Group (ECOG) performance status 0–1
5. Hormonal therapy will be allowed as per institutional guidelines
6. Left ventricular ejection fraction (LVEF) of ≥ 50% measured by echocardiography (ECHO) or multiple gated acquisition (MUGA) scan prior to first dose of trastuzumab SC, or, for those who were receiving trastuzumab when beginning the study, documented results within an acceptable limit from a cardiac assessment within 3 months prior to enrolment
7. HER2-positive disease immunohistochemistry (IHC)3^{+} or in situ hybridization (ISH) positive, in line with local reimbursement criteria and determined in a local laboratory that is experienced/certified in HER2-expression testing using an accurate and validated assay
8. Histologically confirmed non-metastatic primary invasive adenocarcinoma of the breast
9. No evidence of residual, locally recurrent or metastatic disease after completion of surgery and chemotherapy, (neo-adjuvant or adjuvant). Available patient data supporting continuation of trastuzumab, no restaging required.
10. Use of concurrent curative radiotherapy will be permitted
11. Patients have completed the first 6 cycles of trastuzumab IV as part of the (neo)adjuvant treatment

Exclusion Criteria:

1. History of other malignancy which could affect compliance with the protocol or interpretation of results. Patients with curatively treated carcinoma in situ of the cervix or basal cell carcinoma, and patients with other curatively treated malignancies who have been disease-free for at least 5 years, are eligible
2. Patients with severe dyspnea at rest or requiring supplementary oxygen therapy
3. Patients with other concurrent serious diseases that may interfere with planned treatment, including severe pulmonary conditions/illness
4. Serious cardiac illness or medical conditions that would preclude the use of trastuzumab, specifically: history of documented congestive heart failure (CHF), high-risk uncontrolled arrhythmias, angina pectoris requiring medication, clinically significant valvular disease, evidence of transmural infarction on electrocardiogram (ECG), diagnosed poorly controlled hypertension
5. Known infection with human immunodeficiency virus (HIV), active hepatitis B virus (HBV) or hepatitis C virus (HCV)

6. Pregnant or lactating women. Positive serum pregnancy test in women of childbearing potential, premenopausal or less than 12 months of amenorrhea post-menopause, within 7 days prior to the first dose of study drug
7. Women of childbearing potential, premenopausal or less than 12 months of amenorrhea post-menopause (unless surgically sterile), and male patients with partners of childbearing potential who are unable or unwilling to use adequate contraceptive measures during study treatment. In this study, menopause is defined as a minimum of 12 consecutive months of amenorrhea during which time no other biological or physiological cause had been identified as a potential cause of this state. Examples of adequate contraceptive measures are intrauterine device, barrier method (condoms, diaphragm) also in conjunction with spermicidal jelly, or total abstinence. Oral, injectable, or implant hormonal contraceptives are not acceptable
8. Concurrent enrolment in another clinical trial using an investigational anti-cancer treatment, including hormonal therapy, bisphosphonate therapy and immunotherapy, within 28 days prior to the first dose of study treatment
9. Known hypersensitivity to trastuzumab, murine proteins, to any of the excipients of Herceptin® including hyaluronidase, or the adhesive of the SC device, or a history of severe allergic or immunological reactions, e.g. difficult to control asthma
10. Patients assessed by the investigator to be unable or unwilling to comply with the requirements of the protocol
11. Inadequate bone marrow function (as indicated by any of the following):
    a) Absolute neutrophil count (ANC) < 1,500 / mm^3 (< 1.5 × 10^9/L)
    b) Platelets < 100,000 / mm^3 (< 100 × 10^9/L)

Hemoglobin < 10 g/dL

12. Impaired hepatic function (as indicated by any of the following):
    a) Serum total bilirubin > 1.5 × upper limit of normal (ULN)
    b) Alanine amino transferase (ALT) and/or aspartate amino transferase (AST) > 2.5 × ULN
    c) Alkaline phosphatase (ALP) > 2.5 × ULN
13. Inadequate renal function, as indicated by serum creatinine > 1.5 × ULN

Length of Study

The local study is estimated to last approximately 4 years from enrollment of the first patient up to 1 year of recruitment, 8 months of study treatment, and 2 years of drug free follow-up.

Treatment Duration

Patients with HER2+ eBC who completed the first 6 cycles of trastuzumab IV as part of the (neo)adjuvant treatment can be included to continue to receive 12 cycles of trastuzumab to complete a total of 18 cycles of trastuzumab, unless intolerable toxicity or investigator-assessed disease progression occurs, or the patient withdraws consent.

The first 3 cycles trastuzumab in the study will be administered intravenously in the hospital. The next 3 cycles trastuzumab will be administered subcutaneously with a handheld syringe by a trained HCP in the hospital and the last 6 cycles trastuzumab will be administered subcutaneously with a handheld syringe by a trained HCP at the patients home.

Safety Follow-up

All patients will be followed up until investigator-assessed disease progression occurs, withdrawn consent, loss to follow-up, death or up to 24 months after the patient has received his/her last study treatment, whichever occurs first.

End of Study

Last Patient Last Visit (LPLV) in the follow-up period.

Centers

This is a multicenter study. In total 23 centers will participate.

Safety Outcome Measures

Primary objective is safety and tolerability as a function of collected AEs. AEs will be monitored and documented continuously during study (at each 3-weekly treatment visit and during the post-treatment follow-up, as detailed in Section 5.3.1). Incidence and severity by NCI CTCAE version 4.0 of AEs and serious adverse events (SAEs)

- AEs leading to premature discontinuation of study treatment
- Cardiac safety
  - Cardiac AEs
  - CHF (according to NCI CTCAE version 4.0 and New York Heart Association [NYHA] Classification)
  - LVEF over time. In the event of an asymptomatic decline in LVEF, an algorithm (Appendix 2 in the protocol) will be used to determine whether to continue trastuzumab SC treatment
- Secondary safety assessments will include the following:
  - Exposure to study medication
  - Duration of treatment, follow-up, and safety observation
  - ECOG
  - Concomitant medications
  - Laboratory data, vital signs and physical examination
  - Premature withdrawals and major protocol violations

Patient-Reported Outcome Measures

- Patient experience with the treatment provided during the in-hospital part of the study: Patients will be asked to complete a questionnaire (PSQ1, appendix 6) related to the quality of care provided during trastuzumab administration in the hospital prior to the first dose trastuzumab SC vial administered at home = prior to cycle13.

- Patient experience with the treatment provided during the at home part of the study: Patients will be asked to complete a questionnaire (PSQ2, appendix 6) related to the quality of care provided during trastuzumab SC vial administration at home prior to the fifth dose trastuzumab SC vial administered at home = prior to cycle 17.

- Patient reporting of symptoms using the MD Anderson Symptom Inventory (MDASI, appendix 4): Patients will be asked to rate the severity of 13 core items on a scale from 0 to 10. Patients will be asked to complete the MDASI 4 times: Prior to the first dose trastuzumab IV = prior to cycle 7, prior to the first dose trastuzumab SC vial = prior to cycle 10, prior to the first dose trastuzumab SC vial administered at home = prior to cycle 13 and prior to the fourth dose trastuzumab SC vial administered at home = prior to cycle 16.

HCP-Reported Outcome Measures

- HCP overall satisfaction and perceived time savings with trastuzumab SC vial in the hospital will be assessed by a Health Care Professional Questionnaire (HCPEX-1, appendix 5): The HCPEX-1 questionnaire will be completed by a HCP administering trastuzumab IV and SC vial in the hospital after at least 3 patients completed the in hospital part of the study (3 patients must have completed treatment period 1 and 2).

Other outcome Measures

Patients will be interviewed by a HCP to assess:

- Patient experience with trastuzumab IV and SC vial administered in the hospital.

  Patients will be interviewed by a HCP to complete the PEX-P1 (appendix 7) prior to the third dose trastuzumab SC = prior to cycle 12.

- Patient experience with trastuzumab SC vial administered at home.

  Patients will be interviewed by a HCP to complete the PEX-P2 (appendix 7) at the safety follow-up visit, following 4 weeks after the last trastuzumab SC vial administration at home.

Investigational Medicinal Products

Test Product

Both trastuzumab IV and SC are regarded as investigational medicinal product in this study.

Trastuzumab SC

Trastuzumab SC will be administered subcutaneously with a fixed dose of 600 mg (irrespective of body weight) throughout the study treatment phase q3w for 9 cycles. Concurrent curative radiotherapy or anti-hormone therapy will be allowed as per institutional guidelines.

In this local study, trastuzumab SC will be supplied as a vial for manual administration via hand-held syringe, trastuzumab will be subcutaneously injected slowly over a period of up to 5 minutes.

Vial: ready to use solution (600 mg trastuzumab/5 ml) for manual administration using a hand-held syringe administered by HCP.

Trastuzumab IV.

As a three-weekly regimen the recommended initial loading dose of trastuzumab IV is 8 mg/kg body weight. The recommended maintenance dose of trastuzumab IV at three-weekly intervals is 6 mg/kg body weight, beginning three weeks after the loading dose.

Trastuzumab IV loading dose should be administered as a 90-minute intravenous infusion. Do not administer as an intravenous push or bolus. Herceptin intravenous infusion should be administered by a health-care provider prepared to manage anaphylaxis and an emergency kit should be available. Patients should be observed for at least six hours after the start of the first infusion and for two hours after the start of the subsequent infusions for symptoms like fever and chills or other infusion-related symptoms. Interruption or slowing the rate of the infusion may help control such symptoms. The infusion may be resumed when symptoms abate.

If the initial loading dose was well tolerated, the subsequent doses can be administered as a 30-minute infusion.

Comparator

Not applicable.

Procedures (summary)

The complete schedule of assessment is provided in Appendix 1 of the protocol.

Statistical Methods

The primary safety population will be defined as all enrolled patients who received at least one dose of trastuzumab IV study medication.

The secondary safety population will be defined as all enrolled patients who received at least one dose of trastuzumab SC study medication.

All summaries of safety data will be based on the Safety population.

The Intent-to-Treat (ITT) population will be defined as all enrolled patients. All baseline summaries and efficacy analyses will be based on the ITT population.

Study Analysis Methods

Primary Analysis

The primary analysis of the safety endpoints will be undertaken once all patients have completed the study treatment phase and safety follow-up 4 weeks after their last dose of study treatment. Overall safety follow-up 24 months subsequently to the first safety follow-up visit.

The incidence of AEs and SAEs will be summarized according to the primary system-organ class (SOC) and within each SOC, by the Medical Dictionary for Regulatory Activities (MedDRA) preferred term.

Summaries will include the incidence of AEs and SAEs, AEs leading to premature discontinuation of study treatment, and specific cardiac AEs and SAEs. Summaries will include frequency counts and percentages. For certain AEs (or groups of AEs), e.g. cardiac AEs, 95% confidence intervals (calculated using Clopper-Pearson methodology) for incidences will be provided.

Secondary safety parameters include summaries of LVEF, exposure to study medication, duration of treatment and follow-up, vital signs, weight, height, ECOG, concomitant medications, laboratory parameters, premature withdrawals, and major protocol violations.

Secondary analysis

The secondary analyses of patients' experience, patient reporting of symptoms and health care professional experience and overall satisfaction will be undertaken once all patients have completed the study treatment phase and safety follow-up 4 weeks after their last dose of study treatment)

Details will be described in the statistical analysis plan that will be finalized before data base lock.

Determination of Study Sample Size

One hundred subjects are foreseen to be enrolled to assess the overall safety and tolerability of trastuzumab SC vial when administered at home. This sample size was not based on a formal calculation but mainly driven by feasibility and a reasonable width of a 95% confidence interval for the rate of AEs and SAEs. Based on an expected rate of AEs of 97% (23) and 100 subjects, a 95% confidence interval of 91% to 99% would be obtained. For an expected rate of SAEs of 21% (ref. 23) and 100 subjects, a 95% confidence interval ranging from 13% to 29% would be obtained.

LIST OF ABBREVIATIONS AND DEFINITIONS OF TERMS

Abbreviation	Definition
AE	adverse event
ALP	alkaline phosphatase
ALT	alanine aminotransferase
ANC	absolute neutrophil count
AST	aspartate aminotransferase
AUC	area under the serum concentration-time curve
BC	breast cancer
BUN	blood urea nitrogen
CHF	congestive heart failure
CI	confidence interval
C_{max}	maximum concentration
CNS	central nervous system
CRO	contract research organization
CT	computed tomography
CTCAE	common terminology criteria for adverse events
DCIS	ductal carcinoma in situ
DLT	dose-limiting toxicity
DoR	duration of response
eBC	early breast cancer
EC	Ethics Committee
ECG	electrocardiogram
ECHO	echocardiography
ECOG	Eastern Cooperative Oncology Group
eCRF	electronic Case Report Form
EDC	electronic data capture
EEA	European economic area
EGRF	epidermal growth factor receptor
EU	European Union
FDA	Food and Drug Administration
Hb	hemoglobin
HBV	hepatitis B virus
HCV	hepatitis C virus
HER2	human epidermal growth factor receptor 2
HIV	immunodeficiency virus
HCP	health care professional

HR	hazard ratio
ICH	International Conference on Harmonization
IHC	immunohistochemistry
IMP	investigational medicinal product
IND	Investigational New Drug (application)
INR	international normalized ratio
IRB	Institutional Review Board
iDMC	Internal Data Monitoring Committee
IRR	infusion-related reaction
ISH	in situ hybridization
ITT	intent-to-treat
IV	intravenous
LVEF	left ventricular ejection fraction
LPLV	last patient, last visit
mBC	metastatic breast cancer
MedDRA	Medical Dictionary for Regulatory Activities
MRI	magnetic resonance imaging
MUGA	multiple-gated acquisition
NCI	National Cancer Institute
NYHA	New York Heart Association
ORR	overall response rate
OS	overall survival
pCR	pathological complete response
PD	progressive disease
PET	positron emission tomography
PFS	progression free survival
PK	pharmacokinetic
PR	partial response
PRO	patient-reported outcome
q3w	every 3 weeks
qw	weekly
RECIST	response evaluation criteria for solid tumors
rHuPH20	recombinant humanized hyaluronidase
SAE	serious adverse event
SC	subcutaneous
SD	standard deviation
SID	single-use injection device

SGOT	serum glutamic oxaloacetic transaminase
SGPT	serum glutamic pyruvic transaminase
SOC	system-organ class
ULN	upper limit of normal
WBC	white blood cells
WHO	World Health Organization

1. BACKGROUND

1.1 BACKGROUND ON BREAST CANCER – TREATMENT OPTIONS FOR EARLY BREAST CANCER

Breast cancer (BC) is the most common cancer in women (23% of all cancers), with a global prevalence of more than 1 million patients and an annual mortality rate of approximately 450,000 deaths (American Cancer Society). In Europe and the USA, most BCs are diagnosed when the cancer is still confined to the breast, with or without loco-regional lymph node spread, and can be treated with curative intent. In Europe, around 79% are potentially operable (stage T1-3N0/+M0), 7% are locally advanced (T4NxM0), and 6% are metastatic (M1) at diagnosis (Sant et al. 2003). However, BC remains a major cause of death in women aged between 35 and 59 years.

Surgery is the main modality of local treatment for early breast cancer (eBC) and (with or without radiotherapy) can control loco-regional disease in the majority of patients. However, a significant percentage of patients relapse after loco-regional treatment and develop metastases. Systemic chemotherapy or endocrine therapy in hormone receptor-positive disease reduce the risk of relapse and are given either prior to surgery (neoadjuvant therapy) or following surgery (adjuvant therapy). In recent decades, the use of adjuvant systemic therapies for eBC has increased extensively and has most likely contributed to the substantial decline in BC mortality observed in the U.S. and in some European countries (Verma et al. 2010; Colozza et al. 2006, Ferlay et al. 2007).

In the last few years, there has been accelerated progress in the treatment of eBC, with the introduction of taxanes and aromatase inhibitors, and, most impressively, trastuzumab to the adjuvant portfolio (Colozza et al. 2006). Cytotoxic chemotherapy, endocrine therapy, radiotherapy, and molecular targeted therapies currently represent the backbone of modern systemic BC treatment. Several targeted drugs with different molecular pathways have received approval for metastatic breast cancer (mBC), but trastuzumab is the only such therapy that is currently approved for adjuvant and neoadjuvant treatment of eBC (Untch 2010). The use of trastuzumab in the adjuvant setting is supported by international treatment guidelines for women with HER2-positive BC (NCCN 2012; Gnant et al. 2011; Aebi et al. 2011). The introduction of trastuzumab last decade has improved the outcome for eBC patients with HER2-positive disease.

1.2 BACKGROUND ON STUDY TREATMENTS

1.2.1 HUMAN EPIDERMAL GROWTH FACTOR RECEPTOR 2 (HER2)

The human epidermal growth factor receptor 2 (HER2, HER2/neu, c-erbB-2) gene, first discovered in 1984 (Schechter et al. 1984), is localized to chromosome 17q and encodes a transmembrane tyrosine kinase receptor protein that is a member of the epidermal growth factor receptor (EGRF), or HER, family (Ross et al. 2009). This HER family of four receptors mediates the growth, differentiation and survival of cells (Sundaresan et al. 1999; Yarden and Sliwkowski 2001; Gschwind et al. 2004). There is clear evidence that increased expression and activity of HER2 induces cell transformation and tumorigenesis. In BC, unlike a variety of other epithelial

malignancies, HER2 gene amplification is associated with HER2 (p185neu) protein overexpression.

HER2 gene amplification and/or protein overexpression has been associated with aggressive tumor behaviour, including increased cell proliferation, cell motility, tumor invasiveness, progressive regional and distant metastases, accelerated angiogenesis, and reduced apoptosis and poor prognosis (Ross et al. 2009; Slamon et al. 1987; Slamon et al. 1989; Sjögren et al. 1998; Moasser 2007; Ménard et al. 2001). A review of 107 studies involving 39,730 BC patients found that in the majority (88%) of the studies, either HER2 gene amplification or HER2 (p185neu) protein overexpression predicted BC outcome by either univariate or multivariate analysis (Ross et al. 2009). In current practice, most investigators report that the HER2-positive rate is in the range of 15%–20% (Ross et al. 2009; Lund et al. 2010). The major slide-based HER2 testing approaches include immunohistochemistry (IHC), fluorescence in situ hybridization, and chromogenic in situ hybridization.

HER2 amplified BCs comprise a specific disease subset with a unique molecular portrait and biologic characteristics that distinguish them from other types of BCs (Moasser 2007; Crowe et al. 2006). Studies have shown that women whose tumors exhibit either amplification of the HER2 gene or overexpression of its encoded protein have a more aggressive form of BC that is associated with significantly shortened disease-free and overall survival (OS) compared with women whose tumors do not over express HER2 (Dawood et al. 2010).

Evidence indicates that dysregulation of ligands and receptors of the HER family are important in the pathogenesis of cancer. HER receptors normally exist as inactive monomers. Activation of HER receptors occurs following ligand binding, leading to receptor dimerization and cell signaling through the PI3-kinase/AKT pathway for promotion of tumor cell survival and through the mitogen-activated protein kinase pathway for cellular proliferation. Overexpression of HER2 in BC has been correlated with high histological grade, increased mitotic activity, p53 mutation, negative estrogen receptor (ER) status, absence of bcl2, and absence of lobular architecture. Despite associations with other known negative prognostic factors, HER2 overexpression has been independently associated with poorer disease-free survival (DFS) and OS compared with tumors that do not overexpress HER2 (Pauletti et al. 2000, Ménard et al. 2001). Approximately 65% of BCs are ER-positive and progesterone receptor-positive (American Cancer Society).

Until the advent of HER2-targeted agents, patients who had HER2-positive eBC faced a markedly poorer prognosis, including reduced relapse-free and OS, compared to patients with HER2-negative eBC (Ménard et al. 2001; Ross et al. 1998).

1.2.2 TRASTUZUMAB (RHUMAB HER2, HERCEPTIN®)

Trastuzumab is a humanized monoclonal antibody directed against the extracellular domain of HER2. It is indicated for the treatment of patients with HER2-positive mBC (first approved in 1998) and eBC (approved in 2005) and HER2-positive metastatic gastric cancer (approved in 2010). Since its initial approval in 1998, trastuzumab has become standard of care for patients with HER2-positive BC and is widely used for its approved indications in both the adjuvant and metastatic settings (Ross et al. 2009; NCCN 2012; Gnant et al. 2011; Aebi et al. 2011).

The addition of trastuzumab to standard chemotherapy increases time to progressive disease or the length of progression-free survival (PFS), and improves survival when given with

chemotherapy to women with HER2-positive BC (Romond et al. 2005; Slamon et al. 2001). Clinical benefits are greatest in patients with tumors strongly overexpressing HER2, graded 3+ by IHC, and/or with HER2 gene amplification (see Herceptin® Summary of Product Characteristics, 2006).

A randomized Phase II study evaluated trastuzumab and docetaxel vs. docetaxel alone as a first-line treatment for HER2-positive mBC. The addition of trastuzumab to 100 mg/m^2 docetaxel for at least six cycles resulted in superior clinical efficacy with improved overall response rates (ORR), time to progressive disease, time to treatment failure, and duration of response (Marty et al. 2005).

Trastuzumab is well tolerated both as a single agent and in combination with standard chemotherapy (Cobleigh et al. 1998; Slamon et al. 2001). The most clinically relevant adverse event (AE) observed in patients who received trastuzumab was cardiac dysfunction, reflected by asymptomatic decreases in LVEF and, less frequently, by clinically symptomatic congestive heart failure (CHF). Risk factors for cardiac failure in the setting of trastuzumab treatment include co-administration with anthracycline-based chemotherapy, increasing age, declining LVEF during treatment to below the lower limit of normal, and the use of anti-hypertensive medications (Tan-Chiu et al. 2005).

Currently, the marketed formulation of trastuzumab is for intravenous administration (trastuzumab IV, Herceptin®). The majority of available clinical data have been obtained with trastuzumab IV.

For the regulatory status and approved indications in specific countries, please refer to local prescribing information.

1.2.2.1 Trastuzumab IV

The efficacy and safety of trastuzumab IV have been well characterized. Trastuzumab IV is administered to eBC patients for a total duration of 18 cycles q3w. Adjuvant trastuzumab IV may be given as monotherapy, starting after completion of adjuvant chemotherapy, or in combination with the taxane component of adjuvant chemotherapy (followed by trastuzumab monotherapy). At the time of writing, adjuvant trastuzumab IV monotherapy is widely approved, and concurrent administration in combination with adjuvant chemotherapy is also approved or expected to be approved in many countries. Trastuzumab IV may be given weekly (q1w) or 3-weekly (q3w) to patients with mBC but in the adjuvant setting, when given as monotherapy, it is generally given q3w.

For the regulatory status and approved indications in specific countries, please refer to the current Herceptin (Ro 45-2317, Trastuzumab) Investigator's Brochure (IB) and local prescribing information.

1.2.2.2 Pharmacokinetics of Trastuzumab IV

Based on a population pharmacokinetic (PK) analysis of data primarily from the mBC setting (Herceptin Report No. 1018264) the predicted median AUC (over a period of 3 weeks at steady-state) for the q1w and q3w regimens were 1677 and 1793 mg*day/L, respectively, and the corresponding median Cmin values were 64.9 and 47.3 mg/L, respectively. A two-compartment model satisfactorily described the data. The typical trastuzumab IV PK parameters were as

follows: clearance (CL) of 0.026 L/day and a volume of distribution of the central compartment (Vc) of 3.17 L (which corresponds to human plasma volume, which is the Vc characteristic of IgG immunoglobulins). The equilibrium half-life is about 26 days which is similar to that of endogenous IgG1 immunoglobulin (23 days) which constitutes the backbone of trastuzumab IV.

Refer to the Herceptin (Ro 45-2317, Trastuzumab) IB for further details regarding the pharmacokinetics of trastuzumab IV.

1.2.2.3 Efficacy of Trastuzumab IV in Early Breast Cancer (Adjuvant Setting)

Six phase III multi-centre randomized controlled trials investigated the efficacy and safety of adjuvant trastuzumab IV in combination with or after standard adjuvant chemotherapy in the treatment of early breast cancer:

- Herceptin Adjuvant (HERA, BO16348) trial (Piccart-Gebhart et al. 2005; Smith et al. 2007; Gianni et al. 2011)
- North Central Cancer Treatment Group trial (NCCTG) N9831 trial (Romond et al. 2005; Perez et al. 2007; Perez et al. 2009; Perez et al. 2011)
- National Surgical Adjuvant Breast and Bowel Project (NSABP) trial B-31 (Romond et al. 2005; Perez et al. 2007; Perez et al. 2009; Perez et al. 2011)
- Breast Cancer International Research Group (BCIRG-006) study (Slamon et al. 2009, Slamon et al. 2011)
- Protocol Adjuvant dans le Cancer du Sein (PACS04) trial (Spielmann et al. 2009)
- Finland Herceptin (FinHer) trial (Joensuu et al. 2009).

Together, these trials accrued more than 15,000 women with node-positive or high-risk node-negative BC and used a variety of cytotoxic agents in various combinations, doses, and orders of administration. Four of these trials (HERA, N9831, B31 and BCIRG-006) are considered pivotal.

In the HERA study, trastuzumab treatment was started following completion of an approved neoadjuvant or adjuvant chemotherapeutic regimen (and radiotherapy as indicated) and continued for one or two years. In studies B31, N9831 and BCIRG-006, trastuzumab started after completion of four cycles of doxorubicin/cyclophosphamide and was administered for one year, either concurrently with four cycles of taxane chemotherapy (B31, N9831), or concurrently with six cycles of a non-anthracycline-containing taxane-based regimen (BCIRG-006), or after completion of chemotherapy.

All four pivotal randomized controlled trials (HERA, N9831, B31 and BCIRG-006) demonstrated significantly improved DFS, and three (HERA, B31 and BCIRG-006) demonstrated significantly improved OS. The DFS benefits were observed regardless of age, nodal status, hormonal status, or tumour size in all trials (Gianni et al, 2011; Slamon et al. 2011; Perez et al. 2011). Importantly, the most recent follow-up data from the HERA trial (Gianni et al. 2011) and the combined analysis of the NCCTG N9831 and NASBP B-31 trials (Perez et al. 2011) both demonstrate consistent DFS and OS advantages of adjuvant trastuzumab over a median follow-up of 4 years. Further, the significant benefits in DFS and OS were maintained over a median follow-up of approximately 5 ½ years in the BCIRG-006 study (Slamon et al. 2011), which is the longest follow-up reported to

date. The long-term clinical benefits of one-year trastuzumab treatment clearly continue to outweigh the risks of adverse effects (Perez et al. 2011) and the regimen is considered standard of care with support from all major treatment guidelines (NCCN 2012; Gnant et al. 2011; Aebi et al. 2011).

Of the four pivotal randomized trials, the N9831 study was the only one to directly compare the concurrent and sequential use of trastuzumab. This study identified a strong trend for a 25% reduction in the risk of an outcome event when trastuzumab is started concurrently as compared to sequentially after paclitaxel (Perez et al. 2009). Therefore, based on a positive risk/benefit ratio, the authors recommended that trastuzumab be incorporated in a concurrent fashion when administered with paclitaxel (Perez et al. 2009), which also resulted in the approval of the concurrent use of trastuzumab and chemotherapy.

For further details, refer to the current Herceptin (Ro 45-2317, Trastuzumab) IB.

1.2.2.4 Safety of Trastuzumab IV

1.2.2.4.1 Cardiac Safety of Trastuzumab IV

The most clinically relevant AE associated with trastuzumab IV is left ventricular cardiac dysfunction (e.g. CHF). In patients with HER2-positive eBC enrolled in pivotal clinical trials described in Section 1.2.2.3, trastuzumab treatment for 1 year (administered concurrently or sequentially with chemotherapy) appeared to be associated with a decrease in LVEF, an increase in the incidence of CHF (where specified, this was severe [New York Heart Association or NYHA] class III or IV or grade 3 or 4 or symptomatic CHF) and discontinuation of treatment as a result of cardiac AEs (Garnock-Jones et al. 2010). Cardiac toxicity described as NYHA class III/IV CHF occurred in 0%–0.9% of patients in the control arms and in 0%–3.8% of patients in the trastuzumab-containing arms of the four pivotal trials (HERA, N9831, B31 and BCIRG-006). However, the cardiotoxicity observed with concurrent or sequential trastuzumab treatment appeared to be mostly reversible following trastuzumab discontinuation, and no significant increase in cardiac death was reported (Garnock-Jones et al. 2010).

An overview of cardiac safety data from selected trials of trastuzumab in combination with a taxane after anthracyclines for HER2-overexpressing eBC shows rates of symptomatic or severe CHF of < 4% and asymptomatic declines in left ventricular ejection fractions of > 10 points in ≤ 30% of patients. However, inter-study comparisons of chemotherapy-induced cardiac dysfunction are difficult because of the use of different definitions of cardiac dysfunction and different parameters for assessing cardiac safety (Ewer and O’Shaughnessy 2007). These levels were considered below safety cut-off points set by the individual studies’ independent data monitoring committees (Jahanzeb et al. 2008).

The NSABP B-31 trial determined the 5-year cumulative cardiac event rate (NYHA class III or IV CHF or cardiac death) to be 3.8% in patients randomly assigned to trastuzumab versus 0.9% in patients who received chemotherapy alone (Rastogi et al. 2007, Russell et al. 2010). In the NCCTG N9831 trial, the incidence of CHF was 0% in the chemotherapy-alone arm, 2.2% in patients who received sequential chemotherapy and trastuzumab, and 3.3% in patients who received concurrent chemotherapy and trastuzumab (Perez et al. 2008). An independent adjudication of the cardiac events occurring in studies B-31 and N9831 determined that the

incidence of symptomatic heart failure events was 2.0% in trastuzumab-treated patients compared with 0.45% in the chemotherapy-alone arm, and that and the majority (86%) of these patients recovered with appropriate treatment (Russell et al. 2010).

The long-term incidence of cardiac AEs in patients with eBC who were treated with trastuzumab IV for 1 year after completion of neoadjuvant or adjuvant chemotherapy was also evaluated in the HERA trial. Of the 1,698 patients randomly assigned to observation and 1,703 randomly assigned to 1 year of trastuzumab treatment, 94% had been treated with anthracyclines. The incidence of discontinuation of trastuzumab because of cardiac disorders was low (5.1%). At a median follow-up of 3.6 years, the incidence of cardiac end points remained low, though it was higher in the trastuzumab group than in the observation group (severe CHF, 0.8% vs. 0.0%, respectively; confirmed significant LVEF decreases, 3.6% vs. 0.6%, respectively). In the trastuzumab group, 59 of 73 patients with a cardiac end point reached acute recovery (Procter et al. 2010).

1.2.2.4.2 Post-marketing Safety Summary of Trastuzumab IV

It is estimated that over one million patients have been treated with trastuzumab IV as of October 2011 (Roche, Data on file).

The most common (occurring in ≥1 out of 10 treated patients) adverse reactions are infusion-associated symptoms such as fever and chills, usually following the first infusion of trastuzumab IV. These symptoms are usually mild to moderate in severity and occur infrequently with subsequent trastuzumab IV infusions in up to 40% of patients. Other very common (≥1/10 patients) adverse reactions include febrile neutropenia, tremor, dizziness, headache, blood pressure changes (increase or decrease), irregular heartbeat, palpitation, cardiac flutter, decreased ejection fraction, dyspnea, wheezing, diarrhea, vomiting, nausea, lip swelling, abdominal pain, erythema, rash, swelling of the face, arthralgia, muscle tightness, myalgia, asthenia, chest pain, fatigue, influenza-like symptoms, infusion-related reaction, and pain.

Some adverse reactions to trastuzumab IV infusion can be serious and include dyspnea, hypotension, elevated blood pressure, wheezing, bronchospasm, tachycardia, reduced oxygen saturation, and respiratory distress. In the post-marketing setting, very rare (<1/10,000) occurrences of severe infusion reactions leading to a fatal outcome have been associated with the use of trastuzumab IV.

Severe pulmonary events leading to death have been reported with the use of trastuzumab IV in the post-marketing setting (4 out of 10,000 treated patients). Signs, symptoms, and clinical findings included interstitial lung disease including pulmonary infiltrates, acute respiratory distress syndrome, pneumonia, pneumonitis, pleural effusion, respiratory distress, acute pulmonary edema, and pulmonary insufficiency. These events may or may not occur as sequelae of infusion reactions. Patients with symptomatic intrinsic lung disease or with extensive tumour involvement of the lungs, resulting in dyspnea at rest, may be at greater risk of severe reactions. Other risk factors associated with interstitial lung disease include prior or concomitant therapy with other anti-neoplastic therapies such as taxanes, gemcitabine, vinorelbine and radiation therapy.

In addition, severe hypersensitivity reactions have been infrequently reported in patients treated with trastuzumab IV (the exact incidence of these events is unknown). Signs and symptoms include anaphylaxis, urticaria, bronchospasm, angioedema, and/or hypotension. In some cases, the reactions have been fatal. Symptom onset generally occurred during an infusion, but onset

after the completion of an infusion has also been reported. Reactions were most commonly reported in association with the initial infusion.

The immunogenicity of trastuzumab IV has been investigated in clinical studies that included 903 MBC patients. Human anti–human antibodies to trastuzumab were detected in one patient, who had no allergic manifestations.

More detailed information on the full safety profile of trastuzumab IV is found in the Herceptin (Ro 45-2317, Trastuzumab) IB.

1.2.2.5 Trastuzumab SC

Trastuzumab for subcutaneous (SC) administration has been developed by F. Hoffmann-La Roche Ltd to address the known limitations of IV administration (e.g. infusion-related reactions, long administration times, requirement for hospital facilities, treatment barrier for patients with poor venous access, continued use of port-a-cath systems). Administration of trastuzumab SC takes significantly less time (up to 5 minutes) compared to IV infusion (30 to 90 minutes) and this is expected to improve treatment convenience and compliance. These attributes are particularly important for patients treated over prolonged periods of time, such as in the adjuvant setting. In clinical studies conducted to date, administration of trastuzumab SC is associated with a reduced frequency and intensity of administration-related reactions. Such reduction in adverse effects of administration has also been observed with another monoclonal antibody, alemtuzumab (MabCampath®) (Lundin et al. 2002). Treatment fatigue has been reported to lead to premature treatment cessation in a small proportion of patients treated with chemotherapy (Coates et al. 1983).

In the HannaH study (a Phase III randomized, open-label, international study of the SC formulation of trastuzumab in HER2-positive eBC patients), the safety profile of trastuzumab SC was comparable to that of trastuzumab IV (Jackisch et al. 2012). Subcutaneous injection of trastuzumab SC formulation was generally well tolerated with a low incidence of injection site reactions (grades 1 and 2). These findings support the potential of trastuzumab SC to provide improved convenience for patients compared to the existing IV formulation (Pivot et al. 2012). In addition, SC administration also offers the potential for administration of trastuzumab outside a hospital/outpatient clinic setting in the future, further improving convenience and compliance.

The feasibility and patient acceptability of subcutaneous administration of any drug is dependent on the volume that must be administered. A key excipient in the subcutaneous formulation is the enzyme hyaluronidase, which enables larger volumes to be administered without a decrease in patient acceptability. Animal-derived hyaluronidase has been available commercially for over 60 years and is used primarily as a permeation enhancer to increase the dispersion and absorption of other co-administered drugs. Hyaluronidase transiently hydrolyses hyaluronan, a matrix component of the subcutaneous matrix. The hydrolysis leads to decreased viscosity of the subcutaneous matrix and, thus, to an improved delivery of subcutaneous administered drugs to the systemic circulation. The decreased viscosity is also expected to facilitate subcutaneous administration of larger volumes of fluid.

For information on the subcutaneous formulation please refer to the BP22023, CP2 (Section 1.2.2.7.1) and BO22227, HannaH studies (Section 1.2.2.7.2).

More recently, preparations of recombinant humanized hyaluronidase (rHuPH20) have become available. rHuPH20 has a higher purity and is associated with improved efficacy and tolerability compared with the animal-derived enzyme. In the United States, one recombinant humanized hyaluronidase (Hylenex® [Hylenex: Full Prescribing Information]) is licensed to facilitate the absorption and dispersion of drugs when given subcutaneous at doses between 50 IU and 300 IU (Frost et al. 2007). The rHuPH20 used in this study is generated from a second generation of the Hylenex® process (Gen 2 process) with an improved yield and purity.

1.2.2.5.1 Recombinant human hyaluronidase (rHuPH20)

1.2.2.5.1.1 Non-clinical Studies with rHuPH20

After IV administration in the dose range 0.3 to 30 mg/kg, rHuPH20 demonstrated nonlinear PK, rapid clearance and a half-life of around 5 minutes at the lowest dose tested. The bioavailability of rHuPH20 following subcutaneous administration was extremely low (not determinable at low doses, 6% to 8% in the dose range 3 to 30 mg/kg). Treatment of various species with rHuPH20 (IV or SC) was generally well tolerated and no major abnormalities were noted in any toxicology studies.

For details on non-clinical studies with rHuPH20 please refer to the Herceptin® Investigator's Brochure.

1.2.2.5.1.2 Clinical Studies with rHuPH20

The safety and efficacy of hyaluronidase products have been widely established. The most significant safety risk identified is hypersensitivity/allergenicity, which is thought to be related to the lack of purity of the animal-derived preparations (Frost et al. 2007, Harris 2003). The purity and hence the safety risks of hyaluronidase preparations have further been enhanced by the development of the humanized recombinant enzyme rHuPH20. Clinical data are available from four studies with rHuPH20.

- In an allergic sensitivity study (R04-0851), 100 healthy volunteers were injected intradermally with 0.1 ml (15 U) of rHuPH20 and saline control. The most common side effects were generally mild redness, bruising, swelling, discomfort and itching. No AEs were serious and none were judged to be related to study treatment

- A proof-of-concept dose escalation study (HZ2-06-02) with adalimumab and rHuPH20 in 15 patients with rheumatoid arthritis evaluated the effects of rHuPH20 on the PK, safety and tolerability of adalimumab. A single co-administration of adalimumab with rHuPH20 increased adalimumab exposure by a weighted average of 13% compared to adalimumab alone. The injection was well tolerated with only mild and moderate AEs

- HZ2-07-01 was a double-blinded, within-subject-controlled, two way cross-over study comparing the time to inject (flow rate), safety and tolerability of a subcutaneous administered 10% (2,000 mg in 20 ml) solution of immunoglobulin G (diluted Carimune® NF) with and without rHuPH20 in 30 healthy volunteers. There was a statistically non-significant trend towards a decrease in time to inject and an increase in flow rate in the presence of rHuPH20 relative to the control group. The most common AEs were injection

site reactions, consisting of erythema, pain, edema, induration or pruritus (communication Halozyme Therapeutics Inc. on preliminary study results)

- HZ2-07-02 investigated the subcutaneous injection of different rHuPH20 concentrations in a viscous solution of IgG and adalimumab in healthy volunteers using different volumes of injection (2, 8 and 16 ml). The maximum total enzyme dose administered in this study was 96,000 U. The injections were well tolerated with no serious adverse events (SAEs) reported. All injection site reactions such as erythema, pain and induration were mild (98%) or moderate (2%) in severity. There was a trend to lower mean time to inject in subjects who received rHuPH20 compared to those who received injections without rHuPH20, as well as a trend towards an increase in the exposure to adalimumab in the presence of rHuPH20. Pain increased across all volume cohorts after injection, with no clear difference between the presence and absence of rHuPH20. The highest total rHuPH20 dose administered in the clinical studies was 96,000 U and this was well tolerated by healthy volunteers.

Overall, the results of these studies have shown that rHuPH20 is generally well tolerated, with no SAEs reported. AEs were mild or moderate in severity and most were injection site reactions. The highest total rHuPH20 dose administered in the clinical studies was 96,000 U and this was well tolerated by healthy volunteers.

For detailed information on the clinical trials conducted with rHuPH20, please refer to the Herceptin® Investigator's Brochure.

1.2.2.6 Non-clinical Studies with Trastuzumab SC

An overview of completed non-clinical pharmacology, PK, and toxicology studies for trastuzumab subcutaneous is provided in the Herceptin® Investigator's Brochure. Overall, these studies showed that rHuPH20 enabled more rapid absorption of trastuzumab SC, and that subcutaneous administration of trastuzumab formulated with rHuPH20 was well tolerated locally and systemically.

1.2.2.7 Clinical Studies with Trastuzumab SC

Trastuzumab SC (formulated with rHuPH20) has been reported in one completed clinical trial (BP22023, CP2) (Wynne et al. 2010) and one ongoing clinical trial in eBC (BO22227, HannaH) using conventional syringe and hypodermic needle for administration. In addition, the recently completed study (BO25532, CP3) demonstrated comparable exposure between subcutaneous administration with the single-use injection device (SID) versus with the conventional syringe and needle (Wynne et al. 2012). A patient preference and health care professional (HCP) satisfaction study (MO22982, PrefHer) comparing trastuzumab subcutaneous administration using SID or hand-held syringe with conventional administration of trastuzumab IV administration is currently ongoing. A Phase III prospective study (MO28048, SafeHer) to assess the safety of trastuzumab SC (manual administration via hand-held syringe or SID) as adjuvant therapy in patients with operable HER2-positive eBC has been recently initiated.

An overview of the clinical development of Trastuzumab SC is presented in Table 1. As shown a pharmacokinetics study is completed and several phase II and III studies are ongoing.

Table 1 Overview of the Clinical Development Program of SC Trastuzumab

Studies	Status	Design	Primary Objective
**Trastuzumab SC (vial)**			
Phase Ib Dose-finding Study (BP22023, CP2)	Completed	Dose-finding/dose confirmation study<br>OL, PG, single dose, MC	Select the dose of trastuzumab SC which results in comparable exposure to that achieved from an IV dose of trastuzumab
Phase III Clinical Study (BO22227, HannaH)	Completed	PK, efficacy and safety study in the neoadjuvant/adjuvant setting<br>OL, PG, randomized, multiple-dose, MC	Non-inferiority of pre-surgery trastuzumab C_{trough} and pCR
Phase I Device Qualification Study (BO25532, CP3)	Completed	PK bridging to injection device<br>OL, PG, randomized, single dose, MC	PK comparability of trastuzumab SC dosing via a SID or via hand-held needle and syringe used in previous clinical studies.
**Additional Studies**			
Phase II Patient Preference Study (MO22982, PrefHER)	Ongoing	Patient preference and HCP satisfaction study<br>Randomized, MC, cross-over study and PK study	To evaluate patient preference for trastuzumab SC administration using a SID/ hand-held needle and syringe or trastuzumab IV
Phase III Safety Study [MO28048, SafeHer]	Ongoing	Safety Study<br>Non-randomized two-cohort, MC, OL	To evaluate the safety of assisted- and self-administered trastuzumab SC as adjuvant therapy
HCP: Health Care Professional; MC: Multi-centre; OL: Open-label; pCR: pathological complete response; PG: Parallel group; PK: pharmacokinetic; SID: single use injection device			

See the Herceptin Investigator's Brochure for details on clinical studies.

1.2.2.7.1 Study BP22023

Study BP22023 (CP2, Wynne et al. 2010) was a dose-finding study of trastuzumab SC, conducted in healthy male volunteers and patients with HER2-positive eBC. Part 1 of the study was designed to select a trastuzumab SC dose (formulated with rHuPH20) that resulted in comparable exposure and trough levels at least as high as those achieved with trastuzumab IV at dose of 6 mg/kg. Part 2 of the study was designed to confirm the subcutaneous dose selected from part 1. Twenty-four healthy male subjects and 42 female patients with HER2-positive eBC received single doses of either IV or trastuzumab SC.

In Part 1, a total of 86 AEs were observed in 27 subjects/patients. Of these, 71% were considered to be mild, 28% moderate and there was one SAE (an infusion-related reaction [IRR]).

In Cohort 1, in which male healthy volunteers received trastuzumab IV at 6 mg/kg, the most commonly observed AEs were headache (3 AEs), musculoskeletal pain (2), diarrhea (2), abdominal pain (2) and IRR (2).

In Cohort 2, in which female patients received trastuzumab IV at 6 mg/kg, the most commonly observed AE was headache which occurred in 2 patients.

In Cohorts 3 to 5, in which male subjects received trastuzumab SC at 6, 8 and 10 mg/kg, there was no apparent dose-related increase in AEs and subcutaneous administration was generally well tolerated. The most commonly observed AEs were headache (4 events; 3 mild, 1 moderate), upper respiratory tract infection (4 events; all mild) and influenza-like illness (3 events; 2 moderate, 1 mild).

In Part 2, a total of 181 AEs were observed in 39 female patients. Of these, 72.5% were considered to be mild, 25.5% moderate and there were four (2%) severe AEs.

In Cohorts A and B, there was no apparent dose-related increase in AEs and subcutaneous administration was generally well tolerated. The most commonly observed AEs in these patients were headache (27 events; 18 mild, 8 moderate, 1 severe), diarrhea (8 events; 6 mild, 2 moderate), lethargy (6 events; 4 mild, 2 moderate) and injection site erythema (6 events; all mild).

As this was the first study during which subjects/patients received trastuzumab SC, special consideration was given to the local tolerability related to drug administration. In subjects/patients who received trastuzumab SC, there were 18 AEs that were classified as administration site conditions. All but two of these AEs were mild in severity. There were two instances of moderate injection site pain.

No deaths or serious AEs (SAEs) occurred in this study.

1.2.2.7.2 Study BO22227

Study BO22227 (HannaH) compared the PK profile, efficacy, and safety of the subcutaneous and IV formulations in patients with HER2-positive eBC. The HannaH study was a phase III, randomized, international, open-label, trial in the (neo)adjuvant setting.

Patients with HER2-positive, operable, locally advanced or inflammatory BC were randomly assigned to eight cycles of neoadjuvant chemotherapy administered concurrently with trastuzumab every 3 weeks either IV (8 mg/kg loading dose, 6 mg/kg maintenance dose) or subcutaneous (fixed dose of 600 mg); 1:1 ratio. Chemotherapy consisted of four cycles of docetaxel (75 mg/m^2) followed by four cycles of fluorouracil (500 mg/m^2), epirubicin (75 mg/m^2), and cyclophosphamide (500 mg/m^2), every 3 weeks. After surgery, patients continued trastuzumab to complete 1 year of treatment. Co-primary endpoints were serum trough concentration (C_{trough}) at predose cycle 8 before surgery (non-inferiority margin for the ratio between groups of 0.80) and pathological complete response (pCR; non-inferiority margin for the difference between groups of –12·5%), analyzed in the per-protocol population. This study is registered with ClinicalTrials.gov, number NCT00950300. Finding: 299 patients were randomly assigned to receive intravenous trastuzumab and 297 to receive trastuzumab SC. The geometric

mean pre-surgery C_{trough} was 51.8 µg/mL (coefficient of variation 52.5%) in the IV group and 69.0 µg/mL (55.8%) in the subcutaneous group. The geometric mean ratio of C_{trough} subcutaneous to C_{trough} IV was 1.33 (90% CI 1.24–1.44). 107 (40.7%) of 263 patients in the IV group and 118 (45.4%) of 260 in the subcutaneous group achieved a pCR. The difference between groups in pCR was 4.7% (95% CI –4.0 to 13.4). Thus trastuzumab SC was non-inferior to trastuzumab IV for both co-primary endpoints. The incidence of grade 3–5 adverse events was similar between groups. The most common of these adverse events were neutropenia (99 [33.2%] of 298 patients in the IV group vs 86 [29·0%] of 297 in the subcutaneous group), leucopenia (17 [5.7%] vs 12 [4.0%]), and febrile neutropenia (10 [3.4%] vs 17 [5.7%]). However, more patients had SAE in the subcutaneous group (62 [21%] of 297 patients) than in the IV group (37 [12%] of 298); the difference was mainly attributable to infections and infestations (24 [8.1%] in the subcutaneous group vs 13 [4.4%] in the IV group). Four AEs led to death (one in the intravenous group and three in the subcutaneous group), all of which occurred during the neoadjuvant phase. Of these, two—both in the subcutaneous group—were deemed to be treatment related. Trastuzumab SC, administered over about 5 min, has a PK profile and efficacy non-inferior to standard IV administration, with a similar safety profile to trastuzumab IV, and therefore offers a valid treatment alternative. No new safety signals were identified with trastuzumab SC (Ismael et al. 2012).

1.2.2.7.3 Study BO25532

Study BO25532 (CP3) was a randomized, open-label, parallel, 2-arm, multi-centre Phase I study to investigate the comparability of PK of trastuzumab administered subcutaneously using either the SID or a conventional syringe and hypodermic needle. The study also assessed the performance of the SID and evaluated the immunogenicity of trastuzumab and rHuPH20.

Enrolment was completed in September 2011, with a total of 119 healthy male subjects randomized 1:1 to receive a single 600 mg subcutaneous injection by either administration method. The primary objective of the study was met, with the results for both co-primary PK endpoints within the standard bioequivalence range of [0.8, 1.25], meeting the pre-specified criteria for comparability. Sensitivity analyses of the co-primary endpoints that included non-dose normalized or non-body-weight adjusted calculations were in line with the primary analysis.

Trastuzumab was well tolerated after single-dose administration by both methods and no apparent differences related to the injection method were observed (Wynne et al. 2012).

1.2.2.7.4 Ongoing Studies

Study MO22982

MO22982 (PrefHer) is a Phase II international, randomized, open-label, two-cohort, two-arm crossover study to evaluate patient's preference and HCP satisfaction with subcutaneous versus IV administration of trastuzumab in HER2-positive eBC, following surgery and completion of chemotherapy (neoadjuvant or adjuvant). As (neo)adjuvant treatment may also include trastuzumab, randomized patients are stratified by de novo vs. non de novo trastuzumab. Patients in Arm A receive trastuzumab SC (4 cycles) followed by trastuzumab IV (4 cycles). Patients randomized to Arm B receive trastuzumab IV (4 cycles) followed by trastuzumab SC (4 cycles). Patients enrolled into Cohort 1 receive trastuzumab SC administered via a SID, and patients enrolled into Cohort 2 receive trastuzumab SC administered from a vial with a hand-held

syringe. An estimated 200 patients will be randomized to obtain at least 160 evaluable patients in each cohort, giving a total of approximately 400 patients to obtain 320 evaluable patients. The enrollment of Cohort 1 patients is complete, enrollment of Cohort 2 patients is ongoing.

Study MO28048

MO28048 (SafeHer) is a Phase III, prospective, two-cohort, non-randomized, multicenter, multinational, open label study to assess the safety of assisted- and self-administered trastuzumab SC as adjuvant therapy in patients with operable HER2-positive eBC. Approximately 2,500 patients with HER2-positive eBC whose tumor has been excised will be enrolled into the study. The trial is ongoing and will be conducted at approximately 500 centers in approximately 60 countries.

1.3 STUDY RATIONALE AND BENEFIT–RISK ASSESSMENT

Trastuzumab SC is a new route of administration. It has demonstrated similar PK and efficacy in pCR in the treatment of HER2-positive eBC in the neoadjuvant setting as compared to trastuzumab IV. No new safety signals were identified.

Roche is conducting a global phase IIIb Umbrella study to assess the safety and tolerability of trastuzumab solution injected subcutaneously (vial) in patients with HER2-positive eBC.

This sudy is conducted under this umbrella and will there for contribute safety data to a global database for a pooled analysis.

In this Umbrella study, the safety and tolerability of trastuzumab SC will be explored in a wider patient population to gain better understanding of the safety profile of trastuzumab SC in the treatment of HER2-positive eBC patients, whilst addressing additional local/regional scientific questions and investigations in patient sub-populations.

In this local study, the safety and tolerability of trastuzumab SC will be explored further to gain better understanding of the safety profile of trastuzumab SC in the treatment of HER2-positive eBC when administered at home.

Today, patients receive 18 cycles of trastuzuamb IV every 3 weeks as part of the adjuvant treatment. The administration of trastuzumab subcutaneous formulation at home could represent a more convenient option for patients and health care professionals in the future.

1.3.1 RATIONALE FOR TEST PRODUCT DOSAGE

Trastuzumab SC will be given q3w at a fixed dose of 600 mg. Use of a fixed dose for all patients and all cycles greatly simplifies dosing, reduces the potential for error, and reduces wastage. Fixed doses have been used for other therapeutic monoclonal antibodies, particularly in chronic conditions, such as rheumatoid arthritis (e.g. adalimumab). The fixed dose of trastuzumab for subcutaneous administration was selected with the aim of achieving trastuzumab serum trough concentrations (C_{trough}) that are non-inferior to those obtained with q3w trastuzumab IV

administration. The fixed (600 mg) dose of trastuzumab used in this study was calculated based on PK modelling of preliminary data from the BP22023 study (see Section 1.2.2.7.1) which showed that 600 mg doses of trastuzumab SC were able to achieve serum C_{trough} levels at least as high as those achieved with standard weight-adjusted trastuzumab IV dosing. Trastuzumab exhibits linear pharmacokinetics in the clinical dose range, which is an indication that target receptors are saturated. Therefore, achieving C_{trough} levels with subcutaneous administration that are at least as high as with the IV dosing, indicates that efficacy should be comparable. Patients with lower body weight may be exposed to higher C_{trough} levels than if they were dosed on a weight-adjusted basis. However, studies in which higher than standard (or more frequent) doses of trastuzumab were given (Clinical Study Report 1026709; Vogel et al. 2002) and reports of patients accidentally overdosed with trastuzumab IV, do not indicate any detrimental effect on patient safety. Moreover, based on data from the BP22023 study, the predicted maximal concentrations following eight q3w cycles of 600 mg are expected to be below the C_{trough} of trastuzumab IV observed in the MO16982 study (range 199-375 mg/L). In study MO16982 patients were initially dosed with 6 mg/kg weekly and no increase in AEs was observed (Clinical Study Report 1026709). More recently, results of the HannaH study (BO22227) have been released. The study met its two co-primary endpoints, i.e. observed trastuzumab C_{trough} after 7 cycles and the primary efficacy variable of pathological complete response, thereby demonstrating comparable bioavailability and efficacy of the subcutaneous and IV formulations of trastuzumab.

The fixed dose of 600 mg of trastuzumab SC will be administered with a fixed dose of 10,000 U of rHuPH20 (2,000 U/ml). The dose of rHuPH20 was selected based on non-clinical PK studies with a number of antibodies, including trastuzumab. These studies showed a trend for increasing dispersion and absorption with increasing concentrations of rHuPH20 (Clinical Study Report 1029906; Halozyme Study Report). Of note, a higher amount of rHuPH20 (6,000 U/ml) did not improve the absorption of trastuzumab as compared to a formulation containing 2,000 U/ml rHuPH20. The selected rHuPH20 concentration was further verified in clinical studies for satisfactory absorption parameters. Non-clinical and clinical data demonstrate that the selected amount of rHuPH20 contained in the trastuzumab SC formulation is well-tolerated.

1.3.2 RATIONALE FOR PATIENTS WITH EBC

Surgery is the main modality of local treatment for BC (with or without radiotherapy) and can control loco-regional disease in the majority of patients. Systemic chemotherapy (or endocrine therapy in hormone receptor-positive patients) reduces the risk of relapse and is given either prior to surgery (neoadjuvant therapy) or following surgery (adjuvant therapy) or both. Cytotoxic chemotherapy, endocrine therapy, radiotherapy, and molecular targeted therapies currently represent the backbone of modern systemic BC treatment.

Patients with HER2-positive eBC frequently require extensive treatment lasting months or years. Many require surgery, adjuvant chemotherapy (usually given IV for 4-6 months) and/or hormonal therapy, and radiotherapy (often given daily for 4-6 weeks), as well as trastuzumab. Adjuvant trastuzumab is given IV q1w or q3w for a total of 18 cycles.

Trastuzumab is a standard component of adjuvant treatment in patients with HER2-positive eBC It is fact that patients have to attend clinics q3w ubntil 18 infusions of trastuzumab IV are completed.

Trastuzumab infusions are given over 30 to 90 minutes (or longer if there are infusion-related symptoms). Subcutaneous administration of trastuzumab is quicker (lasting up to 5 minutes) and this alone could improve convenience for patients (and clinic staff).

Furthermore, subcutaneous administration does not require IV access (which can be problematic in some patients after completion of chemotherapy) and, based on the findings of the BP22023 study (Wynne et al. 2010) and previous observations with subcutaneous alemtuzumab administration (Lundin et al. 2002), may reduce the incidence of infusion-related symptoms. Use of a SID may also enable self-administration of trastuzumab in the future. This could potentially further improve convenience for patients and compliance with therapy.

The most clinically relevant AE associated with trastuzumab IV is left ventricular cardiac dysfunction or CHF. Cardiac toxicity, as measured by the rate of NYHA class III/IV CHF was the most significant AE, occurring in 0%–3.8% of patients in the trastuzumab-containing arms of six randomized adjuvant trials. The current study is designed to investigate the safety and tolerability of two subcutaneous administration methods for trastuzumab in the adjuvant setting, i.e. assisted administration using a conventional syringe (vial formulation) and self-administration using a SID. The observed incidence of CHF-related SAEs served as basis for the determining the sample size for the current trial. Patient satisfaction with self-administration using a SID is assessed as part of the secondary objectives of the study.

The trastuzumab SC dose selected for this study is consistent with the findings of the BP22023 (CP2) trial and identical to that evaluated in the recently completed BO22227 (HannaH) study (see Section 1.2.2.7.2 for details). Efficacy is hence expected to be comparable to that that observed in trastuzumab IV trials. Safety data from the BP22023 and BO22227 studies show that trastuzumab SC is well tolerated, with no safety signals detected compared to trastuzumab IV in the BO22227 study. The benefit to risk ratio of adjuvant trastuzumab SC in the current trial is therefore expected to be favorable. Further, the convenience of subcutaneous administration of trastuzumab will give patients greater independence which is expected to increase compliance.

Several targeted drugs with different molecular pathways have achieved approval for mBC, but trastuzumab is the only such therapy that is currently approved for adjuvant or neoadjuvant treatment of eBC (Untch 2010). The use of trastuzumab in the adjuvant setting is also supported by international treatment guidelines for women with HER2-positive BC (NCCN 2012; Goldhirsch et al. 2011; Aebi et al. 2011).

The introduction of trastuzumab at the end of 1990s has particularly improved the outcome for eBC patients with HER2-positive disease (Colozza et al. 2006).

1.3.3 BENEFITS OF SC ADMINISTRATION OF TRASTUZUMAB

The current marketed formulation of trastuzumab is for IV administration. Trastuzumab infusions are given over 30 to 90 minutes (or longer if there are infusion-related symptoms).

Subcutaneous administration of trastuzumab is quicker (lasting approximately 5 minutes) and this alone could improve convenience for patients (and clinic staff). Furthermore, subcutaneous administration does not require IV access, which can be problematic in some patients after

completion of chemotherapy. It is expected that with the switch from IV to subcutaneous administration route, HCP time would be saved. Time gained could be invested in other patient care activities, or increase the number of patients that could be treated at a site. Further it is expected that the amount of medical supplies and resources required would be reduced.

Trastuzumab SC (formulated with rHuPH20) has been evaluated in three completed clinical trial: BP22023 (CP2), BO22227 (HannaH) a clinical trial using conventional syringes and hypodermic needles for administration and a PK study BO25532 (CP3) to demonstrate comparable exposure between subcutaneous administration with the SID (Single Injection Device) versus with the conventional syringe and needle. Trastuzumab was well tolerated after single-dose administration by both methods and no apparent differences related to the injection method were observed (Wynne et al. 2012). Two clinical trial with trastuzumab SC are ongoing in patients with eBC: MO22982 (PrefHer) will evaluate patient's preference and HCP satisfaction, and MO28048 (SafeHer) will assess the safety of assisted- and self-administered trastuzumab SC (see Section 1.2.2.7.4)

As mentioned earlier, trastuzumab is now a standard component of adjuvant treatment in patients with HER2-positive eBC, and is supported by all major treatment guidelines. The long-term benefits trastuzumab IV treatments have been demonstrated. The dosing of trastuzumab SC in this study has been chosen to achieve serum trough levels at least as high as those achieved by the IV formulation.

Efficacy of trastuzumab SC is hence expected to be comparable to IV. Trastuzumab IV has an established safety profile, supported by a clinical database comprised of approximately 14,000 female patients with HER2-positive eBC (in addition to patients with HER2-positive MBC). Safety data from the BP22023 and BO22227 studies show that trastuzumab SC is well tolerated, with no new safety signals detected compared to trastuzumab IV in the BO22227 study. The benefit to risk ratio of trastuzumab SC administration in this trial is therefore expected to be favorable. The main advantage of trastuzumab SC is the reduced administration time (approximately 5 minutes) compared to the IV infusions. The convenience of subcutaneous administration of trastuzumab will give patients greater independence which is believed to lead to an improved quality of life.

Beside improving patients convenience and comfort, subcutaneous administration of trastuzumab may also optimize medical resource utilization by reducing administration time, requiring no dedicated infusion staff and no need for infusion bag preparation.

2. OBJECTIVES

2.1 PRIMARY OBJECTIVE

The primary objective of this study is to assess the overall safety and tolerability of subcutaneous (SC) trastuzumab with assisted administration using a conventional syringe and needle (vial formulation, hereafter referred to as trastuzumab SC vial) when administered at home for the treatment of patients with HER2-positive (HER2+) early breast cancer (eBC).

2.2 SECONDARY OBJECTIVES

To describe:

- The evolution of individual patient experience across treatment periods:
    - Patient experience with trastuzumab IV administered in the hospital.
    - Patient experience with trastuzumab SC vial administered in the hospital.
    - Patient experience with trastuzumab SC vial administered at home.
- Patient reporting of symptoms.
- Patient reporting on the quality of care provided during treatment in the hospital and at home.
- HCP overall satisfaction and perceived time savings with trastuzumab SC vial administered in the hospital.
- Disease-free survival (DFS)

3. STUDY DESIGN

3.1 DESCRIPTION OF STUDY

The study is a Phase IIIb, open-label, multinational, multicenter, study to assess the safety and tolerability of trastuzumab subcutaneous formulation when administered at home for the treatment of patients with HER2-positive eBC. Safety data will be collected and transferred to a global database for a pooled analysis.

This study specifically investigates the administration of trastuzumab subcutaneous vial administered at home by a HCP. Collecting information on how patients experience being treated at home is an important aspect of this study. The evolution of individual patient experience across treatment periods will be evaluated. This study incorporates patient reported outcomes for experience and satisfaction with the treatment provided.

Patients with HER2+ eBC who completed the first 6 cycles of trastuzumab IV as part of the (neo)adjuvant treatment can be included to continue to receive 12 cycles of trastuzumab to complete a total of 18 cycles of adjuvant trastuzumab.

The 12 cycles of trastuzumab will be administered according to following schedule:

- Trastuzumab IV will be administered in the hospital by a HCP for the first 3 cycles (Trastuzumab Cycle 7 to 9 defined as treatment period 1)
- Trastuzumab SC vial will be administered in the hospital by a HCP for the next 3 cycles (Trastuzumab Cycle 10 to 12 defined as treatment period 2)
- Trastuzumab SC vial will be administered at home by a HCP for the following 6 cycles (Trastuzumab Cycle 13 to 18 defined as treatment period 3)

The study is expected to include approximately 100 patients.

The study is estimated to last approximately 4 years. Patient enrolment will be staggered according to local study timelines and is expected to take up to 1 year. Patients will be treated in the study for a period of 8 months with a follow-up of 24 months.

Schedules of assessments for patients are provided in Appendix 1.

3.2 PATIENT POPULATION

Patients with HER2-positive early breast cancer who were eligible for a (neo)adjuvant treatment with trastuzumab, eg. clinical stage I (T1, N0, M0) to IIIC (any T, N3, M0) and completed the first 6 cycles of trastuzumab IV as part of the (neo)adjuvant treatment can be included to continue to receive 12 cycles of adjuvant trastuzumab to complete a total of 18 cycles.

3.3 INTERNAL DATA MONITORING COMMITTEE

Adequate monitoring and scientific oversight will be ensured by the country medical monitor and the medical monitor of the data management company. Both roles ensure an adequate monitoring of the safety of the patients.

3.4 LENGTH OF STUDY

The study is estimated to last approximately 4 years from enrollment of the first patient into study (up to 1 year of recruitment 8 months of study treatment, and 2 years of follow-up).

3.5 SAFETY FOLLOW-UP

All patients will be followed up until investigator-assessed disease progression occurs, withdrawn consent, loss to follow-up, death or up to 24 months after the patient has received his/her last study treatment, whichever occurs first.

3.6 END OF STUDY

Last patient last visit (LPLV) in the follow-up period.

3.7 OVERALL NUMBER OF PATIENTS

The study is expected to include approximately 100 patients.

3.8 PATIENT ENROLMENT

100 patients with HER2+ eBC who completed the first 6 cycles trastuzumab IV as part of a (neo)adjuvant treatment, will be enrolled over a period of 12 months in 23 sites in 2 countries (Belgium and Israel).

3.9 COUNTRIES/CENTERS

The study will be performed in 2 countries (Belgium and Israel) with approximately 23 sites.

3.10 RATIONALE FOR STUDY DESIGN

Trastuzumab SC is a new administration route that has demonstrated efficacy and tolerability in the treatment of HER2-positive eBC (see Section 1.2.2). In this study, the safety and tolerability of trastuzumab SC will be explored further to gain better understanding of the safety profile of trastuzumab SC in the treatment of HER2-positive eBC when administered at home.

The administration of trastuzumab subcutaneous formulation at home could represent a more convenient option for patients and health care professionals in the future.

Surgery is the main modality of local treatment for BC (with or without radiotherapy) and can control loco-regional disease in the majority of patients. Systemic chemotherapy (or endocrine therapy in hormone receptor-positive patients) reduces the risk of relapse and is given either prior to surgery (neoadjuvant therapy) or following surgery (adjuvant therapy) or both. Cytotoxic chemotherapy, endocrine therapy, radiotherapy, and molecular targeted therapies currently represent the backbone of modern systemic BC treatment.

Patients with HER2-positive eBC frequently require extensive treatment lasting months or years. Many require surgery, adjuvant chemotherapy (usually given IV for 4-6 months) and/or hormonal therapy, and radiotherapy (often given daily for 4-6 weeks), as well as trastuzumab. Adjuvant trastuzumab is given IV q1w or q3w for a total of one year. This necessitates regular clinic visits and, when started after completion of adjuvant chemotherapy and adjuvant radiotherapy if indicated, it greatly extends the period of time over which the patient is obliged to attend the hospital or clinic which can cause inconvenience and increased costs to patients. Even when started concurrently with the taxane component of chemotherapy (Herceptin license permitting), trastuzumab monotherapy still continues for several months after completion of other systemic therapy.

If safety and compliance are provided, the at home administration of trastuzumab subcutaneous formulation might represent a more convenient treatment option for patients in the future.

3.10.1 RATIONALE FOR POOLING STRATEGY

This study is part of a multi-center, international global umbrella study.

The data from this study and other local studies conducted in various countries will be pooled due to similar designs, endpoints and data structure. A large global database of safety and efficacy data will be created, whilst ensuring these data are collected in a standard, high-quality manner.

Safety and efficacy data will be pooled, summarized and analyzed where appropriate. Data will be further explored using the following subgroups of interest:

- eBC neo-adjuvant
- eBC adjuvant
- mBC first line
- mBC second and later line

- mBC long responders
- trastuzumab SC monotherapy
- trastuzumab SC combination therapy

3.10.2 RATIONALE FOR TEST PRODUCT REGIMEN

Trastuzumab IV and trastuzumab SC are regarded as investigational medicinal products (IMPs).

The regimen consisting of 1 year trastuzumab treatment administered 3-weekly is considered standard of care in patients with eBC, as supported by all major treatment guidelines (NCCN 2011; Goldhirsch et al. 2011; Aebi et al. 2011). This 1-year treatment duration is consistent with the current label for trastuzumab IV. Based on the comparability of treatment parameters, the efficacy of trastuzumab SC is expected to be similar to that observed in trastuzumab IV trials.

Patients have completed the first 6 cycles trastuzumab IV as part of their normal care, before being enrolled in this study. All eligible patients will receive the following 12 cycles trastuzumab in the study.

Trastuzumab IV will be administered for the first 3 cycles in this study unless intolerable toxicity or investigator-assessed disease progression occurs, or the patient withdraws consent. Trastuzumab IV will be administered as a three-weekly regimen. The recommended initial loading dose of trastuzumab IV is 8 mg/kg body weight. The recommended maintenance dose of trastuzumab IV at three-weekly intervals is 6 mg/kg body weight, beginning three weeks after the loading dose.

Trastuzumab IV loading dose should be administered as a 90-minute intravenous infusion. Do not administer as an intravenous push or bolus. Patients should be observed for at least six hours after the start of the first infusion and for two hours after the start of the subsequent infusions for symptoms like fever and chills or other infusion-related symptoms.Interruption or slowing the rate of the infusion may help control such symptoms. The infusion may be resumed when symptoms abate. If the initial loading dose was well tolerated, the subsequent doses can be administered as a 30-minute infusion.

All eligible patients will receive the next 6 cycles trastuzumab subcutaneously. A fixed dose of 600 mg trastuzumab SC throughout the study, administered q3w (irrespective of body weight) for 6 cycles , unless intolerable toxicity or investigator-assessed disease progression occurs, or the patient withdraws consent. Known advantages of the use of a fixed dose for all patients and all cycles include a greatly simplified dosing, reduced potential for error, and reduced wastage. The fixed dose of 600 mg trastuzumab SC was selected with the aim of achieving trastuzumab serum trough concentrations (C_{trough}) that are non-inferior to those obtained with trastuzumab IV administration. Importantly, this dose is consistent with the findings of the BP22023 (CP2) trial and identical to that evaluated in the recently reported BO22227 (HannaH) study.

3.11 OUTCOME MEASURES

3.11.1 SAFETY OUTCOME MEASURES

The safety outcome measures for this study are as follows:

The primary objective is safety and tolerability as a function of collected Aes. AEs will be monitored and documented continuously during study (at each 3-weekly treatment visit and during the post-treatment follow-up, as detailed in Section 5.3.1.

Summaries will include:

- Incidence and severity by NCI CTCAE version 4.0 of AEs and SAEs
- AEs leading to premature discontinuation of study treatment
- Cardiac safety
  - Cardiac AEs
  - CHF (according to NCI CTCAE version 4.0 and New York Heart Association [NYHA] Classification)
  - LVEF over time. In the event of an asymptomatic decline in LVEF, an algorithm (Appendix 2) will be used to determine whether to continue trastuzumab treatment
- Secondary safety assessments will include the following:
  - Exposure to study medication
  - Duration of treatment, follow-up, and safety observation
  - ECOG
  - Concomitant medications
  - Laboratory data, vital signs and physical examination
  - Premature withdrawals and major protocol violations

3.11.2 PATIENT-REPORTED OUTCOME MEASURES

- Patient experience with the treatment provided during the in-hospital part of the study: Patients will be asked to complete a questionnaire (PSQ1 - Appendix 6) related to the quality of care provided during trastuzumab administration in the hospital prior to the first dose trastuzumab SC vial administered at home = prior to cycle 13.

- Patient experience with the treatment provided during the at home part of the study: Patients will be asked to complete a questionnaire (PSQ2 - Appendix 6) related to the quality of care provided during trastuzumab SC vial administration at home prior to the fifth dose trastuzumab SC vial administered at home = prior to cycle 17.

- Patient reporting of symptoms using the MD Anderson Symptom Inventory (MDASI, appendix 4):Patients will be asked to rate the severity of 13 core items on a scale from 0 to 10. Patients will be asked to complete the MDASI 4 times: Prior to the first dose trastuzumab IV = prior to cycle 7, prior to the first dose trastuzumab SC vial = prior to cycle 10, prior to the first dose trastuzumab SC vial administered at home = prior to cycle 13 and prior to the fourth dose trastuzumab SC vial administered at home = prior to cycle 16.

3.11.3 HCP-REPORTED OUTCOME MEASURES

- HCP overall satisfaction and perceived time savings with trastuzumab SC vial in the hospital will be assessed by a Health Care Professional Questionnaire (HCPEX-1): The HCPEX-1 questionnaire will be completed by a HCP administering trastuzumab IV and SC vial in the hospital after at least 3 patients completed the in hospital part of the study (3 patients must have completed treatment period 1 and 2).

3.11.4 OTHER

Patients will be interviewed by a HCP to assess:

- Patient experience with trastuzumab IV and SC vial administered in the hospital.

  Patients will be interviewed by a HCP to complete the PEX-P1 (appendix 7) prior to the third dose trastuzumab SC = prior to cycle 12.

- Patient experience with trastuzumab SC vial administered at home.

  Patients will be interviewed by a HCP to complete the PEX-P2 (appendix 7) at the safety follow-up visit, following 4 weeks after the last trastuzumab SC vial administration at home.

4. MATERIALS AND METHODS

4.1 PATIENTS

The study population will include patients with eBC with Eastern Cooperative Oncology Group (ECOG) performance status 0–1 (Oken et al. 1983). This can include patients with concomitant

hormonal therapy and prior anti-HER2 therapy. Furthermore, patients must have given their informed consent and must not meet any of the exclusion criteria detailed in Section 4.3.

4.2 INCLUSION CRITERIA

Patients and partners must agree to use a barrier method of contraception during the treatment period and for at least 7 months after the last dose of study drug. Please see Section 5.2.3 for further details.

Patients must meet the following criteria for study entry:

1. Female and male patients aged ≥ 18 years
2. Signed informed consent prior to any study specific procedure
3. Able and willing to comply with protocol
4. ECOG performance status 0–1
5. Hormonal therapy will be allowed as per institutional guidelines
6. Left ventricular ejection fraction (LVEF) of ≥ 50% measured by echocardiography (ECHO) or multiple gated acquisition (MUGA) scan prior to first dose of trastuzumab, or, for those who were receiving trastuzumab when beginning the study, documented results within an acceptable limit from a cardiac assessment within 3 months prior to enrolment
7. HER2-positive disease IHC3^{+} or ISH positive as determined in a local laboratory that is experienced/certified in HER2-expression testing using an accurate and validated assay
8. Histologically confirmed non-metastatic primary invasive adenocarcinoma of the breast
9. No evidence of residual, locally recurrent or metastatic disease after completion of surgery and chemotherapy (neo-adjuvant or adjuvant). Available patient data supporting continuation of trastuzumab, no restaging required
10. Use of concurrent curative radiotherapy will be permitted
11. Patients completed the first 6 cycles of trastuzumab IV as part of the (neo)adjuvant treatment

4.3 EXCLUSION CRITERIA

Patients who meet any of the following criteria will be excluded from study entry:

1. History of other malignancy which could affect compliance with the protocol or interpretation of results. Patients with curatively treated carcinoma in situ of the cervix or basal cell carcinoma, and patients with other curatively treated malignancies who have been disease-free for at least 5 years, are eligible
2. Patients with severe dyspnea at rest or requiring supplementary oxygen therapy
3. Patients with other concurrent serious diseases that may interfere with planned treatment, including severe pulmonary conditions/illness

4. Serious cardiac illness or medical conditions that would preclude the use of trastuzumab, specifically: history of documented congestive heart failure (CHF), high-risk uncontrolled arrhythmias, angina pectoris requiring medication , clinically significant valvular disease, evidence of transmural infarction on electrocardiogram (ECG), diagnosed poorly controlled hypertension
5. Known infection with human immunodeficiency virus (HIV), active hepatitis B virus (HBV) or hepatitis C virus (HCV)
6. Pregnant or lactating women. Positive serum pregnancy test in women of childbearing potential, premenopausal or less than 12 months of amenorrhea post-menopause, within 7 days prior to the first dose of study drug
7. Women of childbearing potential, premenopausal or less than 12 months of amenorrhea post-menopause (unless surgically sterile), and male patients with partners of childbearing potential who are unable or unwilling to use adequate contraceptive measures during study treatment. In this study, menopause is defined as a minimum of 12 consecutive months of amenorrhea during which time no other biological or physiological cause had been identified as a potential cause of this state. Examples of adequate contraceptive measures are intrauterine device, barrier method (condoms, diaphragm) also in conjunction with spermicidal jelly, or total abstinence. Oral, injectable, or implant hormonal contraceptives are <u>not</u> acceptable
8. Concurrent enrolment in another clinical trial using an investigational anti-cancer treatment, including hormonal therapy, bisphosphonate therapy and immunotherapy, within 28 days prior to the first dose of study treatment
9. Known hypersensitivity to trastuzumab, murine proteins, to any of the excipients of Herceptin®, or the adhesive of the subcutaneous device, or a history of severe allergic or immunological reactions, e.g. difficult to control asthma
10. Patients assessed by the Investigator to be unable or unwilling to comply with the requirements of the protocol
11. Inadequate bone marrow function (as indicated by any of the following):
    a) Absolute neutrophil count (ANC) < 1,500 / mm^3 (< 1.5 x 10^9/L)
    b) Platelets < 100,000 / mm3 (< 100 x 10^9/L)
    c) Hemoglobin < 10 g/dL
12. Impaired hepatic function (as indicated by any of the following):
    a) Serum total bilirubin > 1.5 x upper limit of normal (ULN)
    b) Alanine amino transferase (ALT) and/or aspartate amino transferase (AST) > 2.5 x ULN
    c) Alkaline phosphatase (ALP) > 2.5 x ULN
13. Inadequate renal function, as indicated by serum creatinine > 1.5 x ULN

4.4 STUDY TREATMENT

Trastuzumab SC vial and trastuzumab IV are regarded as IMP in this study.

4.4.1 FORMULATION, PACKAGING, AND HANDLING

The study drugs will be manufactured by the Sponsor. Study drug packaging will be overseen by the Sponsor clinical trial supplies department and bear a label with the identification required by local law, the protocol number, drug identification and dosage.

The packaging and labeling of the study medication will be in accordance with Sponsor standards and local regulations.

The study drug must be stored according to the details on the Product Information. The drug label indicates the storage temperature.

Upon arrival of investigational products at the site, site personnel should check them for damage and verify proper identity, quantity, integrity of seals and temperature conditions, and report any deviations or product complaints to the Monitor upon discovery.

4.4.1.1 Trastuzumab IV

Herceptin 150 mg powder for concentrate for solution for infusion. One 15 ml clear glass type I vial with butyl rubber stopper laminated with a fluoro-resin film containing 150 mg of trastuzumab.

Appropriate aseptic technique should be used. Each vial of Herceptin is reconstituted with 7.2 ml of water for injections (not supplied). Use of other reconstitution solvents should be avoided.

After reconstitution with water for injections the reconstituted solution is physically and chemically stable for 48 hours at 2°C – 8°C. Any remaining reconstituted solution should be discarded.

Solutions of Herceptin for infusion are physically and chemically stable in polyvinylchloride, polyethylene or polypropylene bags containing sodium chloride 9 mg/ml (0.9%) solution for injection for 24 hours at temperatures not exceeding 30°C.

From a microbiological point of view, the reconstituted solution and Herceptin infusion solution should be used immediately. The product is not intended to be stored after reconstitution and dilution unless this has taken place under controlled and validated aseptic conditions. If not used immediately, in-use storage times and conditions are the responsibility of the user.

Vials of trastuzumab SC should be stored at 2°C to 8°C in appropriate facilities at the institution.

Trastuzumab for intravenous administration will be supplied by Roche. Packaging of trastuzumab for intravenous use will be overseen by the Roche Clinical Trial Supplies department.

For further details, see the trastuzumab IV local prescribing information.

4.4.1.2 Trastuzumab SC

The drug product in the vials for manual injection contains 120 mg/mL trastuzumab. The drug product contains 2000 units/mL rHuPH20 (manufactured in a Chinese hamster ovary [CHO] cell line) acting as a permeation enhancer, histidine/histidine-HCl (buffer), alpha,alpha-trehalose dihydrate (bulking agent), methionine (stabilizer), and polysorbate 20 (stabilizer/emulsifier) in water for injection (WFI) at a pH of 5.5 ± 0.5. Vials of trastuzumab SC should be stored at 2°C to 8°C in appropriate facilities at the institution.

- Ro 045.2317/F07 - Vial for manual subcutaneous injection formulation: Trastuzumab 600 mg/5 mL plus rHuPH20 10000 units/5 mL vial. According to the Medical Device Directive, the drug/device combination is considered an integral medicinal product and therefore as a single IMP for this study.

Trastuzumab for subcutaneous administration will be supplied by Roche. Packaging of trastuzumab for subcutaneous use will be overseen by the Roche Clinical Trial Supplies department. Each IMP unit will bear a label with the identification required by local law, the protocol number, drug identification and dosage. The packaging and labelling of trastuzumab SC will be in accordance with Roche standard and local regulations.

For further details, see the trastuzumab Investigator's Brochure or local prescribing information.

4.4.2 DOSAGE, ADMINISTRATION, AND COMPLIANCE

The trastuzumab regimen in this daughter protocol will only include mono therapy, for the treatment of HER2+ eBC. Patients will receive 12 cycles of trastuzumab to complete a total of 18 cycles of trastuzumab therapy.

Trastuzumab IV will be administered intravenously q3w for the first 3 cycles.

Trastuzumab IV will be administered intravenously in the hospital.
The recommended initial loading dose is 8 mg/kg body weight.

The recommended maintenance dose of Herceptin at three-weekly intervals is 6 mg/kg body weight, beginning three weeks after the loading dose.

If the patient misses a dose of trastuzumab IV by one week or less, then the usual maintenance dose (three-weekly regimen: 6 mg/kg) should be given as soon as possible. Do not wait until the next planned cycle. Subsequent maintenance doses (three-weekly regimen: 6 mg/kg respectively) should then be given according to the previous schedule.

If the patient misses a dose of trastuzumab IV by more than one week, a re-loading dose should be given over approximately 90 minutes (three-weekly regimen: 8 mg/kg). Subsequent Herceptin maintenance doses (three-weekly regimen 6 mg/kg respectively) should then be given (three-weekly regimen every 3 weeks) from that point.

Trastuzumab SC vial will be administered subcutaneously with a fixed dose of 600 mg (irrespective of body weight) q3w for the next 9 cycles.

Concurrent curative radiotherapy or anti-hormone therapy will be allowed as per institutional guidelines.

Trastuzumab SC will be supplied as a vial for manual administration via hand-held syringe and will be subcutaneously injected slowly over a period of up to 5 minutes by a trained HCP.

- **Vial**: ready to use solution (600 mg trastuzumab/5mL and rHuPH20 10000 units/5 mL) for manual administration using a hand-held syringe.

The recommended dose for trastuzumab SC is 600mg/5ml given every 3 weeks. The fixed dose should not be adjusted to the patient's body weight and there is no need for a loading dose on the first administration.

Vials of trastuzumab SC should be stored at 2°C to 8°C in appropriate facilities at the institution. Once removed from the refrigerator, trastuzumab SC must be administered within 6 hours and should not be kept above 25°C.

At all times during this study the trastuzumab SC vials will be stored in appropriate facilities at the institution (hospital pharmacy).

Trastuzumab SC vial is supplied as a ready-to-use solution and does not need to be reconstituted nor diluted.

It is not necessary to prepare trastuzumab SC in a sterile environment, this can be done in the administration room.

During the at home period of the study, trastuzumab SC will be administered at home by trained HCP.

The HCP administering trastuzumab SC vial at home will pick up the unopened trastuzumab SC vial at the pharmacy of the institution. During transport the storage conditions as described above are to be respected.

Empty trastuzumab SC vials need to be returned to the hospital pharmacy on the next pick-up.

If a patient misses a dose of trastuzumab SC, it tis recommended that the next dose of 600mg/5ml be administered as soon as possible. Subsequent doses should be given according to the normal schedule.

Guidelines for dosage modification and treatment interruption or discontinuation are provided in Section 5.1.4.

4.4.3 INVESTIGATIONAL MEDICINAL PRODUCT ACCOUNTABILITY

All IMP required for completion of this study will be provided by the Sponsor. The investigational site will acknowledge receipt of IMP, using the IxRS to confirm the shipment condition and content. Any damaged shipments will be replaced.

The investigator is responsible for the control of the drugs under investigation. Adequate records for the receipt (e.g. Drug Receipt Record) and disposition (e.g. Drug Dispensing Log) of the IMP must be maintained. Accountability and patient compliance will be assessed by maintaining adequate "drug dispensing" and return records.

Accurate records must be kept for each IMP provided by the sponsor. These records must contain the following information:

- Documentation of IMP shipments received from the sponsor (date received, quantity and batch number)
- Disposition of unused IMP not dispensed to a patient
- A Drug Dispensing Log must be kept current and should contain the following information:
    - Identification of the patient to whom the IMP was dispensed;
    - The date(s), quantity and batch number of the IMP dispensed to the patient.

4.4.3.1 Assessment of Compliance

The investigator is responsible for ensuring that the study drug is administered in compliance with the protocol. Delegation of this task must be approved by the investigator and clearly documented. Patient compliance will be assessed by maintaining adequate study drug dispensing records. All records and drug supplies must be available for inspection by the Roche Study Monitor at every monitoring visit.

Copies of the dispensing & inventory logs will be retrieved by the Monitor at study end.

4.4.3.2 Destruction of the IMPs

Used and unused IMP will be kept at the site (or designated pharmacy, depending on local practice) for accountability and destruction. Local or institutional regulations may require immediate destruction of used IMPs for safety reasons e.g., cytotoxicity. In these cases, it may be acceptable for the investigational site staff to destroy the dispensed IMP before inspection by the Monitor, provided that source document verification is performed on the remaining inventory and reconciled against the documentation of quantity shipped, dispensed, returned and destroyed. Written authorization must be obtained from the Sponsor at study start up before destruction of unused trastuzumab SC can take place at a site.

Written documentation of destruction must contain the following:

- Identity (batch numbers and patient numbers) of the IMP(s) destroyed
- Quantity of the IMP(s) destroyed
- Date of destruction (date discarded in designated hazardous container for destruction)
- Method of destruction (the site must provide the Sponsor with documentation of their institutional policy and procedures for handling and disposing of hazardous drugs)
- Name and signature of responsible person who discarded the IMP in a hazardous container for destruction

4.4.4 POST-TRIAL ACCESS TO TRASTUZUMAB SC

Roche does not intend to provide trastuzumab SC (or IV) or other study interventions to patients after conclusion of the study or any earlier patient withdrawal. Subsequent treatment will be at the investigator's discretion and according to local practice.

4.5 CONCOMITANT THERAPY

4.5.1 PERMITTED THERAPY

Concomitant therapy includes any medication (e.g., prescription drugs, over-the-counter drugs, herbal/homeopathic remedies, nutritional supplements) used by a patient from screening to the safety follow-up visit. Thereafter only medication applicable for long term reporting must be reported, including breast cancer treatments, anticancer treatment given to treat a recurrence, medication related to the treatment of SAEs that are applicable for long-term reporting. All concomitant medications should be reported to the investigator and recorded on the Concomitant Medications electronic Case Report Form (eCRF). All protocol-allowed medications taken by the patient for concomitant disease(s) should be continued as necessary during the study and be recorded on the eCRF.

Any medication which is necessary for the management of side effects of trastuzumab, may be used at the discretion of the investigator. Concomitant medication and treatment should be prescribed according to the investigator's judgment and following the local labels for trastuzumab.

Concomitant medication may include:

- Paracetamol (acetaminophen) or other analgesics, and diphenhydramine, chlorpheniramine, or other antihistamines used according to local clinical practice for the prevention and treatment of IRR associated with trastuzumab
- Hormonal therapy for patients with hormone receptor positive disease (according to local clinical practice)
- Bisphosphonates may be given according to their product license and routine clinical practice, at the investigator's discretion, to treat documented osteoporosis
- Palliative surgical procedures. Any diagnostic, therapeutic or surgical procedure performed during the study period should be recorded including the dates, description of the procedure(s), and any clinical findings
- Palliative radiotherapy (if indicated)

4.5.2 PROHIBITED THERAPY

The following therapies are prohibited:

- Concurrent treatment with other systemic HER2-directed immunotherapy
- Concurrent investigational agents or anthracyclines

4.6 STUDY ASSESSMENTS

All patients must provide written informed consent before any study-specific assessments or procedures are performed.

Prior to enrolment in the study, the Investigator will assess each patient with regard to the protocol's inclusion and exclusion criteria to determine his/her eligibility for the study. Patients must fulfill all the entry criteria for participation in the study.

An Eligibility Screening Form documenting the Investigator's assessment of each patient with regard to the protocol's inclusion and exclusion criteria is to be completed by the Investigator.

An Eligibility Screening Failure Log must be maintained by the Investigator.

4.6.1 DESCRIPTION OF STUDY ASSESSMENTS

4.6.1.1 Mandatory Procedure for Enrolment

Once a patient has fulfilled the entry criteria, he/she will be assigned a study number in a consecutive order at each center. Each center will also have a center number assigned by the sponsor.

A patient Enrollment and Identification Code List must be maintained by the Investigator.

The socio-demographic/baseline characteristic data listed below will be captured, where available. For the schedule of mandatory clinical assessments, please refer to appendix 1.

4.6.1.1.1 Timing of Screening and Pretreatment Assessments

Written informed consent for participation in the study must be obtained before performing any study-specific screening tests or evaluations. Informed Consent Forms for enrolled patients and for patients who are not subsequently enrolled will be maintained at the study site.

Please see appendix 1 for the schedule of screening and pretreatment assessments.

4.6.1.1.2 Medical History and Demographic Data

Medical history includes clinically significant diseases, surgeries, BC disease history (including ER/PgR status, prior anti-cancer treatments, current medications and symptoms), and all medications (e.g., prescription drugs, over-the-counter drugs, herbal/homeopathic remedies, nutritional supplements) used by the patient starting from the screening visit. The year of start and end date (when applicable) of the past and current concomitant treatments for eBC, as well as the route and dose, will be collected when available.

Demographic data will include date of birth, gender, and self-reported ethnic origin.

4.6.1.1.3 HER2 Status

To be eligible for the study, patients with eBC must have confirmed HER2-positivity defined as one of the following and in line with local reibursement criteria if applicable:

- A score of 3+ by IHC

- Gene amplification positive by ISH performed by a validated and approved test in a certified and experienced center

4.6.1.1.4 Vital signs and physical examination

Assessment of vital signs assessment includes pulse, blood pressure, body weight, height, and body temperature. Vital signs measurements will be taken while the patient is in a seated position. Height is only measured at screening.

A general physical exam (including a general neurological exam, as clinically indicated) will be performed. Physical examinations will be performed according to local practice; however, particular attention should be given to the cardiovascular system.

Any abnormality identified at screening should be recorded on the General Medical History and Baseline Conditions eCRF. At subsequent visits, new or worsened abnormalities should be recorded as AEs on the Adverse Event eCRF.

4.6.1.1.5 Pregnancy test

A serum pregnancy test in women of childbearing potential will be performed within 7 days prior to the first dose of study treatment. Any positive urine pregnancy test needs to be confirmed by serum pregnancy test.

4.6.1.1.6 Past and current concomitant treatments

All concomitant medications and prior treatments for eBC must be reported in the eCRF starting at the Screening visit.

The year of start and end date (when applicable) of the past and current concomitant treatments for eBC, as well as the route and dose, will be collected when available.

4.6.1.1.7 ECOG performance status

To be eligible for the study, patients should have ECOG PS 0–1 according to the ECOG PS Scale (Oken et al. 1982).

4.6.1.1.8 Baseline safety data

Baseline safety data will be collected as described in Section 4.6.1.2. Baseline safety data include: AEs and SAEs (as described in Section 4.6.1.2.3), cardiac safety (standard 12-lead ECG and LVEF assessments as described in Section 4.6.1.2.3, hematology and biochemistry (as described in Section 4.6.1.2.4) and concomitant medication.

4.6.1.2 Clinical Assessments During Treatment Period

4.6.1.2.1 Timing of Assessments during Treatment

Please see appendix 1 for the schedule of assessments performed during the treatment period.

4.6.1.2.2 Tumor and response evaluations: Breast cancer follow-up

DFS is a secondary efficacy endpoint for patients with eBC in this study. DFS is defined as time from the date of first treatment to the date of local, regional or distant recurrence, contralateral BC or death due to any cause. Diagnosis of BC relapse will be made based on routine clinical, radiological and laboratory criteria as described in Appendix 1. Acceptable methods of confirmation of recurrence include radiology, CT scan, brain scan, ultrasound, or cytology, as per local practice. In case of uncertainty, disease relapse should be confirmed by histological or cytological examination of a suspicious lesion, if possible.

In case of a suspicious recurrence that leads to death quite quickly without having the possibility to confirm relapse of disease, efforts should be made to obtain an autopsy report.

4.6.1.2.2 ECOG performance status

Performance status will be evaluated using the ECOG Performance Status Scale (Oken et al. 1982).

4.6.1.2.3 Safety clinical assessments

The following clinical safety assessments and procedures will be performed. For the schedule of mandatory safety assessments please refer to Appendix 1. For full details of safety assessments please refer to Section 5.1.

4.6.1.2.3.1 Adverse events

The National Cancer Institute (NCI) Common Terminology Criteria for Adverse Events (CTCAE) version 4.0 will be used to evaluate the clinical safety of the treatment in this study. In addition, symptomatic left ventricular dysfunction will be graded according to NYHA classification.

In addition, SAEs and any Cardiac Event related to CHF occurring during the study will be reported to the Sponsor **within 24 hours** of the investigator becoming aware of them.

For other instructions on documenting and handling AEs please refer to Appendix 1 and Section 5.3.

4.6.1.2.3.2 Vital signs and physical examination

These assessments will be performed as described in Section 4.6.1.1.2 for enrolment of patients.

4.6.1.2.3.3 Electrocardiograms

A standard 12-lead ECG needs to be performed as specified in Appendix 1, Schedule of Assessments.

4.6.1.2.3.4 LVEF

LVEF assessments should be performed by either ECHO or MUGA scan within 3 months prior to the first trastuzumab SC administration to be eligible for participation in the study: the same imaging technique should be used per patient throughout the study. ECHO should be the method

of choice for these assessments. LVEF assessment should be performed as per institutional practice.

Symptomatic left ventricular dysfunction (congestive heart failure [CHF]) will be graded according to NCI-CTCAE version 4.0 and the New York Heart Association functional classification. Any patient who develops clinical signs and symptoms suggesting CHF, with the diagnosis confirmed by a suggestive chest X-ray and a drop in LVEF by ECHO or MUGA will be discontinued from the study medication. CHF should be treated and monitored according to standard medical practice. The incidence of CHF will also be recorded throughout the study.

4.6.1.2.3.5 Concomitant medication

All concomitant medications must be reported in the eCRF starting at the Screening visit. These include:

- Date and extent of primary surgery
- Any loco-regional radiation therapy (extent or volume and total dose)
- Any hormonal therapy and/or surgical and radiation-induced ovarian ablation and drug induced ovarian suppression (type, drug name, dose and schedule, anticipated duration of therapy)
- Bisphosphonate therapy
- Any additional medication that is necessary for the management of the patient may be used at the discretion of the Investigator

All concomitant medications are to be reported until the Safety Follow-up visit. Thereafter, only medication applicable for long-term reporting must be reported, including:

- Breast cancer treatments (e.g., hormonal therapy)
- Anti-cancer treatments given to treat a recurrence
- Medications related to the treatment of SAEs that are applicable for long-term reporting (e.g., treatment of heart failure)

4.6.1.2.4 Laboratory assessments

Normal ranges for the study laboratory parameters must be supplied to the Sponsor before the study starts (Appendix 1).

The following laboratory assessments and procedures will be performed in patients with eBC. For the schedule of mandatory laboratory assessments in patients with eBC please refer to Appendix 1. HER2 status determination is described in Section 4.6.1.1.3.

4.6.1.2.4.1 Hematology and biochemistry

Hematology tests include: Hb, WBC and differential, ANC, and platelet count.

Biochemistry tests include: creatinine, urea (BUN), SGPT (ALT), SGOT (AST), total bilirubin, ALP, albumin, sodium, potassium, and calcium.

All hematology and blood chemistry laboratory tests will be completed at local laboratories. Additional hematology and biochemistry tests may be performed as per institutional practice, but these data will not be collected.

4.6.1.2.4.2 Pregnancy test

Pregnancy testing should be completed as clinically indicated for the duration of study treatment and until at least 7 months after the last dose of study treatment. Any positive urine pregnancy test needs to be confirmed by serum pregnancy test.

4.6.1.2.5 Other Assessments During Treatment Period

4.6.1.2.5.1 Patient Reported Outcomes

4.6.1.2.5.1.1 Patient reporting of symptoms

- Patient reporting of symptoms using the MD Anderson Symptom Inventory (MDASI, appendix 4):Patients will be asked to rate the severity of 13 core items on a scale from 0 to 10. Patients will be asked to complete the MDASI 4 times: Prior to the first dose trastuzumab IV = prior to cycle 7, prior to the first dose trastuzumab SC vial = prior to cycle 10, prior to the first dose trastuzumab SC vial administered at home = prior to cycle 13 and prior to the fourth dose trastuzumab SC vial administered at home = prior to cycle 16.

4.6.1.2.5.1.2 Patient reporting of experience and overall satisfaction

- Patient experience with the treatment provided during the in-hospital part of the study: Patients will be asked to complete a questionnaire (PSQ1, appendix 6) related to the quality of care provided during trastuzumab administration in the hospital prior to the first dose trastuzumab SC vial administered at home = prior to cycle 13.

- Patient experience with the treatment provided during the at home part of the study: Patients will be asked to complete a questionnaire (PSQ2, appendix 6) related to the quality of care provided during trastuzumab SC vial administration at home prior to the fifth dose trastuzumab SC vial administered at home = prior to cycle 17.

4.6.1.2.5.2 HCP reported Outcomes

- HCP overall satisfaction and perceived time savings with trastuzumab SC vial in the hospital will be assessed by a Health Care Professional Questionnaire (HCPEX-1, appendix 5): The HCPEX-1 questionnaire will be completed by a HCP administering trastuzumab IV and SC vial in the hospital after at least 3 patients completed the in hospital part of the study (3 patients must have completed treatment period 1 and 2).

4.6.1.3.5.3 Other

Patients will be interviewed by a HCP to assess:

- Patient experience with trastuzumab IV and SC vial administered in the hospital.

  Patients will be interviewed by a HCP to complete the PEX-P1 (appendix 7) prior to the third dose trastuzumab SC = prior to cycle 12.

- Patient experience with trastuzumab SC vial administered at home. Patients will be interviewed by a HCP to complete the PEX-P2 (appendix 7) at the safety follow-up visit, following 4 weeks after the last trastuzumab SC vial administration at home.

4.6.1.3 Clinical Assessments at the Safety Follow-up visit

Patients who complete the treatment or discontinue from the study early will be asked to return to the clinic 28 days after the last dose of study drug for a safety follow-up visit.

The clinical assessments to be performed at the safety follow-up visit are identical to the assessments during the treatment period (see Section 4.6.1.2) except that no treatment drug will be administered.

Please see Appendix 1 for the schedule of assessments performed at the safety follow-up visit.

4.6.1.4 Clinical Assessments in Follow-up visits

Cardiac assessments at 6, 12, 18 and 24 months following treatment cessation will be performed. At these visits, concomitant medication will be recorded, routine breast cancer follow-up will be performed.

After the safety follow-up visit, AEs should be followed as outlined in Sections 5.5 and 5.6.

Please see Appendix 1 or the schedule of assessments performed in the follow-up visits.

After the follow-up visit, patients will continue follow-up as recommended in routine clinical practice (every 6 months to evaluate disease progression and cardiac function) for a maximum of 24 months following treatment cessation or until disease progression, whichever occurs first.

Please see Appendix 1 for the schedule of assessments performed in the follow-up visits.

4.6.1.5 Follow-Up Assessments

After the safety follow-up visit, AEs should be followed as outlined in Sections 5.5 and 5.6.

Please see Appendix 1 for the schedule of follow-up assessments.

4.6.1.6 Assessments at Unplanned Visits

Please see Appendix 1 for assessments that are required to be performed in case of an unplanned visit.

4.7 PATIENT, STUDY, AND SITE DISCONTINUATION

4.7.1 PATIENT DISCONTINUATION

The Investigator has the right to discontinue a patient from study drug or withdraw a patient from the study at any time. In addition, patients have the right to voluntarily discontinue study drug or withdraw from the study at any time for any reason. Reasons for discontinuation of study drug or withdrawal from the study may include, but are not limited to, the following*:*

- Patient withdrawal of consent at any time
- Any medical condition that the Investigator or Sponsor determines may jeopardize the patient's safety if he or she continues in the study
- Investigator or Sponsor determines it is in the best interest of the patient
- Patient non-compliance, specifically defined as non-compliance with the study procedures, the schedule of assessments or protocol-defined timelines

4.7.1.1 Discontinuation from Study Drug

Patients must discontinue study drug if they experience any of the following:

- Changes in LVEF (see Appendix 2)
- Pregnancy: A patient must be instructed to stop taking the test "drug" and immediately inform the Investigator if she becomes pregnant during the study.

Patients who discontinue study drug prematurely will be asked to return to the clinic for a safety follow-up visit (see Section 5.6) and may undergo follow-up assessments (see Section 4.6.1.4). The primary reason for premature study drug discontinuation should be documented on the appropriate eCRF. Patients who discontinue study drug prematurely will not be replaced.

4.7.1.2 Withdrawal from Study

Every effort should be made to obtain information on patients who withdraw from the study. The primary reason for withdrawal from the study should be documented on the appropriate eCRF. Patients will not be followed for any reason after consent has been withdrawn. Patients who withdraw from the study will not be replaced.

4.7.2 STUDY AND SITE DISCONTINUATION

The Sponsor has the right to terminate this study at any time. Reasons for terminating the study may include, but are not limited to, the following:

- The incidence or severity of AEs in this or other studies indicates a potential health hazard to patients.
- Patient enrollment is unsatisfactory.

The Sponsor will notify the Investigator if the study is placed on hold, or if the Sponsor decides to discontinue the study or development program.

The Sponsor has the right to replace a site at any time. Reasons for replacing a site may include, but are not limited to, the following:

- Excessively slow recruitment
- Poor protocol adherence
- Inaccurate or incomplete data recording
- Non-compliance with the International Conference on Harmonization (ICH) guideline for Good Clinical Practice

5. ASSESSMENT OF SAFETY

5.1 SAFETY PLAN

Trastuzumab IV will be administered intravenously.

The recommended maintenance dose of Herceptin at three-weekly intervals is 6 mg/kg body weight, beginning three weeks after the loading dose.

Trastuzumab SC vial will be administered subcutaneously at a fixed does of 600 mg regardless of body weight.

5.1.1 GENERAL SAFETY ASSESSMENTS

Patients will be assessed by prior medical history, vital signs (including blood pressure, heart rate, temperature), weight and height (screening only), physical examination, AEs and concomitant medications. A complete medical history (including demographic profile and prior treatments for cancer) will be documented at screening. A general physical exam (including general neurological exam, as clinically indicated) will be performed at screening, every cycle during trastuzumab SC treatment, at the post-treatment safety follow-up visit and subsequently according to the ASCO 2006 Guideline for Breast Cancer Follow-up in the adjuvant setting (Khatcheressian et al. 2006) during the follow-up period (see Appendix 1, Schedule of Assessments). During physical examination, particular attention should be given to the cardiovascular system. Apart from physical exams, subcutaneous injection sites will be checked at every visit and blood pressure will be measured before and after trastuzumab SC administration every 3 cycles.

AEs will be monitored and documented continuously during study (at each 3-weekly treatment visit and during the post-treatment follow-up, as detailed in Section 5.3.1). Serious adverse events (SAEs) will also be monitored, documented and reported; refer to Sections 5.4.2 and 5.5 for details on SAE reporting and follow-up requirements, respectively. All AEs and SAEs (including patients' symptoms and signs of toxicity and clinically significant haematological and biochemical parameters) will be graded according to National Cancer Institute (NCI) Common Terminology Criteria for Adverse Events (CTCAE) version 4.0 (see Appendix 3). Changes in concomitant medication will be recorded at each study visit.

Trastuzumab will be given as specified in Section 4.4.2.

Before starting a new treatment cycle, toxicity must have resolved as specified in the following sections.

Trastuzumab administration may be delayed to assess or treat AEs such as cardiac AEs, myelosuppression, or other events. No dose reduction will be allowed for trastuzumab.

5.1.2 CARDIAC SAFETY ASSESSMENTS

Cardiac function will be evaluated regularly throughout the study, by measuring LVEF using echocardiography, MUGA scan or MRI (method selected according to local practice), ECG, and assessment of cardiac signs and symptoms.

Cardiac safety assessments will be performed at screening, approximately 3-monthly during trastuzumab treatment with the results available prior to trastuzumab administration, at the Safety Follow-up visit (LVEF assessment may occur prior to the day of the Safety Follow-up visit if clinically indicated) and then at 6, 12, 18 and 24 months after treatment cessation and immediately as per Section 5.1.3 in case of cardiac failure; see Appendix 1, Schedule of Assessments.

5.1.2.1 LVEF Assessment

The screening LVEF assessment should be performed within 3 months prior to first trastuzumab IV administration. To be eligible for participation in this study, patients must have a baseline LVEF ≥ 50%. The method of assessment (ECHO, MUGA, or MRI) is at the Investigator’s discretion; however, to the extent possible, the same imaging technique is to be used for each patient throughout the study. LVEF assessment results must be available before/on the day of the next scheduled trastuzumab administration, and, should a reduction in LVEF be noticed compared to screening, a decision to give or hold that dose must be made based on the algorithm provided in Appendix 2. In addition, any patient who develops clinical signs or symptoms suspicious of cardiac failure at any time during the study should undergo an LVEF assessment immediately.

Of note, if MUGA scans are chosen, Investigators must be aware that there may be local guidelines which govern how many MUGA scans (or the amount of irradiation) a patient is allowed to have in a year, and must ensure that patients are able to adhere to the cardiac assessment schedule as outlined in Appendix 1 In case additional LVEF assessments become necessary for the medical management of a patient, the Investigator may use echocardiography instead of a MUGA scan to remain within the locally accepted amount of irradiation.

Symptomatic left ventricular dysfunction (congestive heart failure) will be graded according to NCI-CTCAE version 4.0 and the New York Heart Association (NYHA) functional classification (see Appendix 3).

5.1.3 MANAGEMENT OF SPECIFIC AEs

Cardiac safety will be monitored throughout the study, as described in Section 5.1.2. In addition to the scheduled assessments, any patient who develops clinical signs or symptoms suspicious of cardiac failure at any time during the study should undergo an LVEF assessment immediately. Patients whose LVEF falls ≥ 10 percentage points from screening and to a LVEF < 50% for eBC patients may require temporary or permanent cessation of trastuzumab in accordance with the

treatment continuation/discontinuation algorithm shown in Appendix 2. A repeat LVEF assessment should be performed approximately 3 weeks later. If the LVEF has not improved or has declined further, trastuzumab should be discontinued. All such patients should be referred for assessment by a cardiologist and followed up. Trastuzumab should also be discontinued in any patient who develops clinically significant heart failure; see Section 4.6.1.2.2.

5.1.3.1 Infusion-Associated Symptoms and Allergic Reactions

Administration of monoclonal antibodies, including trastuzumab, may cause infusion-associated symptoms such as fever, chills, hypotension, shortness of breath, skin rashes, headache, nausea, vomiting, or allergic reactions. Patients with extensive pulmonary disease, e.g., lymphangitis, multiple metastases, recurrent pleural effusions, and those with preexisting pulmonary compromise who are treated with trastuzumab, may be at increased risk of serious infusion-associated symptoms. Therefore, careful consideration must be made before enrolling patients with chronic lung disease into the study.

Study treatment will be administered in a setting with emergency equipment and staff that is trained to monitor for and respond to medical emergencies. Patients who experience an NCI-CTCAE version 4.0 Grade 4 allergic reaction, acute respiratory distress syndrome (ARDS), or bronchospasm will be discontinued from study treatment.

Patients who experience infusion-associated symptoms may be managed by:

- Slowing or stopping the trastuzumab infusion
- Supportive care with oxygen, beta-agonists, antihistamines, antipyretics, or corticosteroids as appropriate at the Investigator's discretion

Premedication with corticosteroids, antihistamines, and antipyretics may be used before subsequent trastuzumab infusions at the Investigator’s discretion.

If infusion-associated symptoms occur, patients will be monitored until complete resolution of signs and symptoms.

5.1.4 MANAGEMENT OF SPECIFIC ADVERSE EVENTS

5.1.4.1 Dose Modifications, Interruptions and Delays for Trastuzumab

Administration of trastuzumab SC may be delayed to assess or treat AEs, as detailed in table 2.

Table 2 Action to be Taken in Case of Trastuzumab-Related Toxicity

Toxicity related to trastuzumab study treatment	Action pertaining to trastuzumab SC*
1. Non-hematological, grade 1 or 2	Continue trastuzumab SC therapy

Toxicity related to trastuzumab study treatment	Action pertaining to trastuzumab SC*
(excluding cardiac) toxicity	
2. Non-hematological, grade 3 or 4 (excluding cardiac) toxicity and toxicity resolved within a maximum of 5 weeks calculated from last planned administration	Hold trastuzumab SC therapy until recovery to grade ≤ 2.
3. Non-hematological, grade 3 or 4 (excluding cardiac), toxicity NOT resolved to grade <2 or disappeared within a maximum of 5 weeks calculated from last planned administration	Discontinue trastuzumab SC permanently.
4. Recurrence of non-hematological grade 3 or 4 (excluding cardiac) toxicity upon re-challenge	Discontinue trastuzumab SC permanently.
5. Cardiac toxicity: asymptomatic drop in LVEF ≥ 10 percentage points from screening and to a LVEF < 50%	Trastuzumab SC therapy to be held, continued or resumed according to the algorithm depicted in Appendix 2.
6. Cardiac toxicity: symptomatic CHF	Discontinue trastuzumab SC permanently.
7. Cardiac toxicity: other than significant asymptomatic LVEF drop or CHF	Actions must follow rules 1 to 3 for non-hematological toxicities
8. Hematological toxicity	Trastuzumab needs not to be withheld for hematological toxicity

* Concurrent anti-cancer/endocrine therapy (as applicable) may continue at the Investigator's discretion.

If the patient misses a dose of trastuzumab SC, then the usual maintenance dose should be given as soon as possible, with subsequent maintenance doses given q3w. No dose adjustment is needed in case of delayed administration of trastuzumab SC as a fixed (600 mg) dose of trastuzumab is given for all subcutaneous cycles in this study.

Dose reductions are not permitted for toxicity. Patients who experience infusion-related or injection-related symptoms may be pre-medicated with paracetamol and antihistamines for subsequent infusions/injections.

5.2 SAFETY PARAMETERS AND DEFINITIONS

Safety assessments will consist of monitoring and recording AEs, including SAEs; measurement of protocol-specified safety laboratory assessments; measurement of protocol-specified vital

signs; and other protocol-specified tests that are deemed critical to the safety evaluation of the study.

Certain types of events require immediate reporting to the Sponsor, as outlined in Section 5.4.

5.2.1 ADVERSE EVENTS

According to the ICH guideline for Good Clinical Practice, an AE is any untoward medical occurrence in a clinical investigation subject administered a pharmaceutical product, regardless of causal attribution. An AE can therefore be any of the following:

- Any unfavorable and unintended sign (including an abnormal laboratory finding), symptom, or disease temporally associated with the use of a medicinal product, whether or not considered related to the medicinal product
- Any new disease or exacerbation of an existing disease (a worsening in the character, frequency, or severity of a known condition), except as described in Section 5.3.5.9
- Recurrence of an intermittent medical condition (e.g., headache) not present at baseline
- Any deterioration in a laboratory value or other clinical test (e.g., ECG, X-ray) that is associated with symptoms or leads to a change in study treatment or concomitant treatment or discontinuation from study drug
- AEs that are related to a protocol-mandated intervention, including those that occur prior to assignment of study treatment (e.g., screening invasive procedures such as biopsies)

5.2.2 SERIOUS ADVERSE EVENTS (IMMEDIATELY REPORTABLE TO THE SPONSOR)

An SAE is any AE that meets any of the following criteria:

- Fatal (i.e., the AE actually causes or leads to death)
- Life threatening (i.e., the AE, in the view of the Investigator, places the patient at immediate risk of death)

    This refers to an event in which the patient was at risk of death at the time of the event; it does not refer to an event which hypothetically might have caused death if it was more severe

- Requires or prolongs inpatient hospitalization (see Section 5.3.5.10)
- Results in persistent or significant disability/incapacity (i.e., the AE results in substantial disruption of the patient's ability to conduct normal life functions)
- Congenital anomaly/birth defect in a neonate/infant born to a mother exposed to study drug
- Significant medical event in the IInvestigator's judgment (e.g., may jeopardize the patient or may require medical/surgical intervention to prevent one of the outcomes listed above)

The terms “severe” and “serious” are not synonymous. Severity refers to the intensity of an AE (rated as mild, moderate, or severe, or according to NCI CTCAE criteria; see Section 5.3.3); the

event itself may be of relatively minor medical significance (such as severe headache without any further findings).

Severity and seriousness need to be independently assessed for each AE recorded on the eCRF.

Serious AEs are required to be reported by the Investigator to the Sponsor within 24 hours after learning of the event (see Section 5.4.2 for reporting instructions).

5.2.3 PREGNANCY AND CONTRACEPTION

In patients of childbearing potential and women < 1 year after the onset of menopause, appropriate contraceptive measures are mandatory during study treatment (examples: barrier method [condoms, diaphragm]; intra-uterine devices; surgical methods, or abstinence). Based on PK considerations, contraceptive measures are recommended for at least 7 months following the last dose of trastuzumab.

A patient must be instructed to stop taking the test “drug” and immediately inform the Investigator if she becomes pregnant during the study. The Investigator should report all pregnancies within 24 hours to the Sponsor, using the Clinical Trial Pregnancy Reporting Form, [gcp_for000023]. The Investigator should counsel the patient, discuss the risks of continuing with the pregnancy, and the possible effects on the fetus. Monitoring of the patient should continue until conclusion of the pregnancy. Pregnancies occurring up to 7 months after the completion of trastuzumab must also be reported to the Investigator.

For male patients with a female partner of childbearing potential, cooperation of female partner is required (i.e., use of two forms of contraception as stated above) during the study and for at least 7 months following the last dose of study treatment when a highly effective form of contraception is not appropriate.

Due to potential dual risk of embryo-fetal toxicity (such as oligohydramnios) resulting from systemic exposure to rHuPH20 and trastuzumab, the need for strict adherence to the guidance on contraceptive usage should be reinforced. Should a woman become pregnant during the active treatment phase of a subcutaneous trastuzumab trial her participation should be ended. Should a woman choose to continue with both the pregnancy and trastuzumab treatment, a multi-disciplinary team should closely follow her. There is no evidence to suggest that male exposure to rHuPH20 poses a risk to the developing foetus.

5.3 METHODS AND TIMING FOR CAPTURING AND ASSESSING SAFETY PARAMETERS

The Investigator is responsible for ensuring that all AEs (see Section 5.2.1 for definition) are recorded on the Adverse Event eCRF and reported to the Sponsor in accordance with instructions provided in this section and in Sections 5.4–5.6. The Investigator is also responsible for reporting medical device complaints (see Section 5.4.4).

For each AE recorded on the Adverse Event eCRF, the Investigator will make an assessment of seriousness (see Section 5.2.2 for seriousness criteria), severity (see Section 5.3.3), and causality (see Section 5.3.4).

5.3.1 ADVERSE EVENT REPORTING PERIOD

Investigators will seek information on AEs at each patient contact. All AEs, whether reported by the patient or noted by study personnel, will be recorded in the patient's medical record and on the Adverse Event eCRF.

After informed consent has been obtained **but prior to initiation of study drug**, only SAEs caused by a protocol-mandated intervention should be reported (e.g., SAEs related to invasive procedures such as biopsies).

After initiation of study drug, all AEs/SAEs, regardless of relationship to study drug, will be reported until study closure. The investigator does not need to actively monitor subjects for adverse events once the trial has ended. However, if becoming aware of any serious adverse events and non-serious adverse events of special interest occurring to a subject, the investigator should report those to the sponsor (see Section 5.6).
Symptomatic congestive heart failure must be reported irrespective of causal relationship during the full course of the study, even if the patient starts a new anticancer regimen.

5.3.2 TABLE 3 ELICITING ADVERSE EVENT INFORMATION

A consistent methodology of non-directive questioning should be adopted for eliciting AE information at all patient evaluation timepoints. Examples of non-directive questions include the following:

"How have you felt since your last clinic visit?"

"Have you had any new or changed health problems since you were last here?"

5.3.3 ASSESSMENT OF SEVERITY OF ADVERSE EVENTS

The AE severity grading scale for the NCI CTCAE (v4.0) will be used for assessing AE severity. Table 4 will be used for assessing severity for AEs that are not specifically listed in the NCI CTCAE.

Table 4 Assessment of AE Severity

CTC Grade	Equivalent To:	Definition
Grade 1	Mild	Mild; asymptomatic or mild symptoms; clinical or diagnostic observations only; or intervention not indicated
Grade 2	Moderate	Moderate; minimal, local, or non-invasive intervention indicated; or limiting age-appropriate instrumental activities of daily living [a]
Grade 3	Severe	Severe or medically significant, but not immediately life-threatening; hospitalization or prolongation of hospitalization indicated; disabling; or limiting self-care activities of daily living [b,c]
Grade 4	Life threatening	Life-threatening consequences or urgent intervention indicated [d]
Grade 5	Death	Death related to adverse event [d]

[a] Instrumental activities of daily living refer to preparing meals, shopping for groceries or clothes, using the telephone, managing money, etc.

[b] Examples of self-care activities of daily living include bathing, dressing and undressing, feeding one's self, using the toilet, and taking medications, as performed by patients who are not bedridden.

[c] If an event is assessed as a "significant medical event," it must be reported as a serious adverse event (see Section 5.4.2 for reporting instructions), per the definition of SAE in Section 5.2.2.

[d] Grade 4 and 5 events must be reported as serious adverse events (see Section 5.4.2 for reporting instructions), per the definition of serious adverse event in Section 5.2.2.

5.3.4 ASSESSMENT OF CAUSALITY OF ADVERSE EVENTS

Investigators should use their knowledge of the patient, the circumstances surrounding the event, and an evaluation of any potential alternative causes to determine whether or not an AE is considered to be related to the study drug, indicating "yes" or "no" accordingly. The following guidance should be taken into consideration:

- Temporal relationship of event onset to the initiation of study drug
- Course of the event, considering especially the effects of dose reduction, discontinuation of study drug, or reintroduction of study drug (where applicable)
- Known association of the event with the study drug or with similar treatments

- Known association of the event with the disease under study
- Presence of risk factors in the patient or use of concomitant medications known to increase the occurrence of the event
- Presence of non-treatment-related factors that are known to be associated with the occurrence of the event

For patients receiving combination therapy, causality will be assessed individually for each protocol-mandated therapy.

5.3.5 PROCEDURES FOR RECORDING ADVERSE EVENTS

Investigators should use correct medical terminology/concepts when recording AEs on the Adverse Event eCRF. Avoid colloquialisms and abbreviations.

Only one AE term should be recorded in the event field on the Adverse Event eCRF.

5.3.5.1 Diagnosis versus Signs and Symptoms

For all AEs a diagnosis (if known) should be recorded on the Adverse Event eCRF rather than individual signs and symptoms (e.g., record only liver failure or hepatitis rather than jaundice, asterixis, and elevated transaminases). However, if a constellation of signs and/or symptoms cannot be medically characterized as a single diagnosis or syndrome at the time of reporting, each individual event should be recorded on the Adverse Event eCRF. If a diagnosis is subsequently established, all previously reported AEs based on signs and symptoms should be nullified and replaced by one AE report based on the single diagnosis, with a starting date that corresponds to the starting date of the first symptom of the eventual diagnosis.

5.3.5.2 Adverse Events Occurring Secondary to Other Events

In general, AEs occurring secondary to other events (e.g., cascade events or clinical sequelae) should be identified by their primary cause, with the exception of severe or serious secondary events. However, medically significant AEs occurring secondary to an initiating event that are separated in time should be recorded as independent events on the Adverse Event eCRF. For example:

- If vomiting results in mild dehydration with no additional treatment in a healthy adult, only vomiting should be reported on the eCRF.
- If vomiting results in severe dehydration, both events should be reported separately on the eCRF.
- If a severe gastrointestinal hemorrhage leads to renal failure, both events should be reported separately on the eCRF.
- If dizziness leads to a fall and subsequent fracture, all three events should be reported separately on the eCRF.
- If neutropenia is accompanied by a mild, non-serious infection, only neutropenia should be reported on the eCRF.

- If neutropenia is accompanied by a severe or serious infection, both events should be reported separately on the eCRF.

All AEs should be recorded separately on the Adverse Event eCRF if it is unclear as to whether the events are associated.

Recurrence of breast cancer (as defined in Section 4.5.1.7.1) should not be reported as an AE since this is clearly consistent with progression/relapse of the underlying disease. Hospitalization due solely to the relapse of underlying malignancy should NOT be reported as an SAE. Clinical symptoms of relapse may be reported as AEs if the symptom cannot be determined as exclusively due to the relapse of the underlying malignancy, or does not fit the expected pattern of relapse for the disease under study.

If there is any uncertainty about an adverse event being due only to the disease under study, it should be reported as an AE or SAE.

5.3.5.3 Persistent or Recurrent Adverse Events

A persistent AE is one that extends continuously, without resolution, between patient evaluation timepoints. Such events should only be recorded once on the Adverse Event eCRF. The initial severity of the event should be recorded, and the severity should be updated to reflect the most extreme severity any time the event worsens. If the event becomes serious, the Adverse Event eCRF should be updated to reflect this.

A recurrent AE is one that resolves between patient evaluation timepoints and subsequently recurs. Each recurrence of an AE should be recorded separately on the Adverse Event eCRF.

5.3.5.4 Abnormal Laboratory Values

Not every laboratory abnormality qualifies as an AE. A laboratory test result should be reported as an AE if it meets any of the following criteria:

- Accompanied by clinical symptoms
- Results in a change in study treatment (e.g., dosage modification, treatment interruption, or treatment discontinuation)
- Results in a medical intervention (e.g., potassium supplementation for hypokalemia) or a change in concomitant therapy
- Clinically significant in the Investigator’s judgment

It is the Investigator’s responsibility to review all laboratory findings. Medical and scientific judgment should be exercised in deciding whether an isolated laboratory abnormality should be classified as an AE.

If a clinically significant laboratory abnormality is a sign of a disease or syndrome (e.g., alkaline phosphatase and bilirubin 5 times the upper limit of normal [ULN] associated with cholecystitis), only the diagnosis (i.e., cholecystitis) should be recorded on the Adverse Event eCRF.

If a clinically significant laboratory abnormality is not a sign of a disease or syndrome, the abnormality itself should be recorded on the Adverse Event eCRF, along with a descriptor

indicating if the test result is above or below the normal range (e.g., "elevated potassium," as opposed to "abnormal potassium"). If the laboratory abnormality can be characterized by a precise clinical term per standard definitions, the clinical term should be recorded as the AE. For example, an elevated serum potassium level of 7.0 mEq/L should be recorded as “hyperkalemia.”

Observations of the same clinically significant laboratory abnormality from visit to visit should not be repeatedly recorded on the Adverse Event eCRF, unless the etiology changes. The initial severity of the event should be recorded, and the severity or seriousness should be updated any time the event worsens.

5.3.5.5 Abnormal Vital Sign Values

Not every vital sign abnormality qualifies as an AE. A vital sign result should be reported as an AE if it meets any of the following criteria:

- Accompanied by clinical symptoms
- Results in a change in study treatment (e.g., dosage modification, treatment interruption, or treatment discontinuation)
- Results in a medical intervention or a change in concomitant therapy
- Clinically significant in the Investigator’s judgment

It is the Investigator’s responsibility to review all vital sign findings. Medical and scientific judgment should be exercised in deciding whether an isolated vital sign abnormality should be classified as an AE.

If a clinically significant vital sign abnormality is a sign of a disease or syndrome (e.g., high blood pressure), only the diagnosis (i.e., hypertension) should be recorded on the Adverse Event eCRF.

Observations of the same clinically significant vital sign abnormality from visit to visit should not be repeatedly recorded on the Adverse Event eCRF, unless the etiology changes. The initial severity of the event should be recorded, and the severity or seriousness should be updated any time the event worsens.

5.3.5.6 Abnormal Liver Function Tests

The finding of an elevated ALT or AST ($> 3 \times$ ULN) in combination with either an elevated total bilirubin ($> 2 \times$ ULN) or clinical jaundice in the absence of cholestasis or other causes of hyperbilirubinemia is considered to be an indicator of severe liver injury. Therefore, Investigators must report as an AE the occurrence of either of the following:

- Treatment-emergent ALT or AST $> 3 \times$ ULN in combination with total bilirubin $> 2 \times$ ULN
- Treatment-emergent ALT or AST $> 3 \times$ ULN in combination with clinical jaundice

The most appropriate diagnosis or (if a diagnosis cannot be established) the abnormal laboratory values should be recorded on the Adverse Event eCRF (see Section 5.3.5.1) and reported to the Sponsor within 24 hours after learning of the event, either as an SAE or a non-serious AE of special interest (see Section 5.4.2).

5.3.5.7 Deaths

Deaths that occur before study closure regardless of relationship to study drug, must be recorded on the Adverse Event eCRF (as an outcome) and immediately reported to the Sponsor (see Section 5.4.2). This includes death attributed to progression of BC. For deaths occurring after this period, please refer to Section 5.6.

A local iDMC will monitor the frequency of deaths from all causes.

Death should be considered an outcome and not a distinct event. The event or condition that caused or contributed to the fatal outcome should be recorded as the single medical concept on the Adverse Event eCRF. Generally, only one such event should be reported. The term **"sudden death"** should only be used for the occurrence of an abrupt and unexpected death due to presumed cardiac causes in a patient with or without preexisting heart disease, within 1 hour of the onset of acute symptoms or, in the case of an unwitnessed death, within 24 hours after the patient was last seen alive and stable. If the cause of death is unknown and cannot be ascertained at the time of reporting, **"unexplained death"** should be recorded on the Adverse Event eCRF. If the cause of death later becomes available (e.g., after autopsy), "unexplained death" should be replaced by the established cause of death.

For eBC, during post-study survival follow-up, deaths attributed to progression of eBC should be recorded only on the Survival eCRF.

5.3.5.8 Preexisting Medical Conditions

A preexisting medical condition is one that is present at the screening visit for this study. Such conditions should be recorded on the General Medical History and Baseline Conditions eCRF.

A preexisting medical condition should be recorded as an AE only if the frequency, severity, or character of the condition worsens during the study. When recording such events on the Adverse Event eCRF, it is important to convey the concept that the preexisting condition has changed by including applicable descriptors (e.g., "more frequent headaches").

Worsening of the Underlying Condition

Medical occurrences or symptoms of deterioration that are anticipated as part of the patient's underlying BC should be recorded as an adverse event if judged by the Investigator to have unexpectedly worsened in severity or frequency or changed in nature at any time during the study. When recording an unanticipated worsening of BC on the Adverse Event eCRF, it is important to convey the concept that the condition has changed by including applicable descriptors (e.g., "accelerated BC").

5.3.5.9 Hospitalization or Prolonged Hospitalization

Any AE that results in hospitalization or prolonged hospitalization should be documented and reported as an SAE (per the definition of SAE in Section 5.2.2), except as outlined below.

The following hospitalization scenarios are not considered to be SAEs:

- Hospitalization for respite care

- Planned hospitalization required by the protocol (e.g., for study drug administration or insertion of access device for study drug administration)}
- Hospitalization for a preexisting condition, provided that all of the following criteria are met:
    - The hospitalization was planned prior to the study or was scheduled during the study when elective surgery became necessary because of the expected normal progression of the disease
    - The patient has not suffered an AE
- Hospitalization due solely to progression of the underlying cancer

5.3.5.10 Overdoses

Study drug overdose is the accidental or intentional use of the drug in an amount higher than the dose being studied. An overdose or incorrect administration of study drug is not an AE unless it results in untoward medical effects.

Any study drug overdose or incorrect administration of study drug should be noted on the Study Drug Administration eCRF.

All AEs associated with an overdose or incorrect administration of study drug should be recorded on the Adverse Event eCRF. If the associated AE fulfills serious criteria, the event should be reported to the Sponsor within 24 hours after learning of the event (see Section 5.4.2).

5.3.5.11 Patient-Reported Outcome Data

AE reports will not be derived from PRO data. However, if any patient responses suggestive of a possible AE are identified during site review of the PRO questionnaires, site staff will alert the Investigator, who will determine if the criteria for an AE have been met and will document the outcome of this assessment in the patient's medical record per site practice. If the event meets the criteria for an AE, it will be reported on the Adverse Event eCRF.

5.4 IMMEDIATE REPORTING REQUIREMENTS FROM INVESTIGATOR TO SPONSOR

The Investigator must report the following events to the Sponsor within 24 hours after learning of the event, regardless of relationship to study drug:

- SAEs
- Cardiac Events related to CHF including abnormality test finding such as LVEF drop
- Pregnancies

The Investigator must report new significant follow-up information for these events to the Sponsor within 24 hours after becoming aware of the information. New significant information includes the following:

- New signs or symptoms or a change in the diagnosis
- Significant new diagnostic test results
- Change in causality based on new information

- Change in the event's outcome, including recovery
- Additional narrative information on the clinical course of the event

Investigators must also comply with local requirements for reporting SAEs to the local health authority and IRB/EC.

All SAEs regardless of the relationship to the study drug or the time elapsed from the last study drug administration, even if the study has been closed ***MUST*** be collected and reported. All participating Investigators and the respective independent Ethics Committees (ECs) will be notified of all Suspected Unexpected Serious Adverse Reactions (SUSARs) that are reported during the study. An AE only qualifies as a SUSAR when all of the following conditions are met:

- The event is serious (SAE);
- The event is deemed related to the study drug, according to the criteria provided in Section 5.3.4. (Note: any suspicion of a causal relationship should lead to an assessment of 'related');
- When assessed against the known safety profile of trastuzumab SC (as described in the IB), the event is considered unexpected (not foreseen in the IB).

When all patients at a particular site are off treatment as defined by the protocol:

- Individual SUSAR reports originating in that particular trial will be forwarded to all participating Investigators and the IECs associated with their sites, on an expedited basis;
- Individual SUSARs considered to be a significant safety issue and/or which result in Roche recommending a change to the Informed Consent Form (ICF), will be reported in an expedited manner to all Investigators and reviewing IECs;
- SUSAR reports originating from other trials using the same IMP will be provided as six-monthly SUSAR Reports (SSRs) to all Investigators and IECs where long-term follow-up studies are carried out.

5.4.1 EMERGENCY MEDICAL CONTACTS

To ensure the safety of study patients, an Emergency Call Center Help Desk will access the Roche Medical Emergency List, escalate emergency medical calls, provide medical translation service (if necessary), connect the investigator with a Roche Medical Monitor, and track all calls. The Emergency Call Center Help Desk will be available 24 hours per day, 7 days per week.

The telephone number for the Help Desk is 00 32 2 525 82 11.

5.4.2 REPORTING REQUIREMENTS FOR SERIOUS ADVERSE EVENTS

For reports of SAEs, Investigators should record all case details that can be gathered within 24 hours on the Adverse Event eCRF and submit the report via the electronic data capture (EDC) system. A report will be generated and sent to Roche Safety Risk Management by the EDC system.

In the event that the EDC system is unavailable, a paper Serious Adverse Event/Non-Serious Adverse Event of Special Interest CRF and Fax Coversheet should be completed and faxed to Roche Safety Risk Management or its designee within 24 hours after learning of the event, using the fax numbers provided to Investigators (see "Protocol Administrative and Contact Information & List of Investigators"). Once the EDC system is available, all information will need to be entered and submitted via the EDC system.

5.4.3 REPORTING REQUIREMENTS FOR PREGNANCIES

5.4.3.1 Pregnancies in Female Patients

For women of childbearing potential (defined as pre-menopausal, less than 1 year after the onset of menopause or not surgically sterilized), appropriate contraceptive measures are mandatory during study treatment (see Section 5.2.3). Based on PK considerations, contraceptive measures are recommended for at least 7 months following the last dose of trastuzumab.

Female patients of childbearing potential will be instructed to immediately inform the Investigator if they become pregnant during the study or within 7 months after the last dose of study drug. A Pregnancy Report eCRF should be completed by the Investigator within 24 hours after learning of the pregnancy and submitted via the EDC system. A pregnancy report will automatically be generated and sent to Roche Safety Risk Management. Pregnancy should not be recorded on the Adverse Event eCRF. The Investigator should discontinue study drug and counsel the patient, discussing the risks of the pregnancy and the possible effects on the fetus. Monitoring of the patient should continue until conclusion of the pregnancy.

In the event that the EDC system is unavailable, a Pregnancy Report worksheet and Pregnancy Fax Coversheet should be completed and faxed to Roche Safety Risk Management or its designee within 24 hours after learning of the pregnancy, using the fax numbers provided to Investigators (see "Protocol Administrative and Contact Information & List of Investigators").

5.4.3.2 Pregnancies in Female Partners of Male Patients

Male patients will be instructed through the Informed Consent Form to immediately inform the Investigator if their partner becomes pregnant during the study or within 7 months after the last dose of study drug. A Pregnancy Report eCRF should be completed by the Investigator within 24 hours after learning of the pregnancy and submitted via the EDC system. Attempts should be made to collect and report details of the course and outcome of any pregnancy in the partner of a male patient exposed to study drug. The pregnant partner will need to sign an Authorization for Use and Disclosure of Pregnancy Health Information to allow for follow-up on her pregnancy. Once the authorization has been signed, the Investigator will update the Pregnancy Report eCRF with additional information on the course and outcome of the pregnancy. An Investigator who is contacted by the male patient or his pregnant partner may provide information on the risks of the pregnancy and the possible effects on the fetus, to support an informed decision in cooperation with the treating physician and/or obstetrician.

In the event that the EDC system is unavailable, follow reporting instructions provided in Section 5.4.3.1.

5.4.3.3 Abortions

Any spontaneous abortion should be classified as an SAE (as the Sponsor considers spontaneous abortions to be medically significant events), recorded on the Adverse Event eCRF, and reported to the Sponsor within 24 hours after learning of the event (see Section 5.4.2).

5.4.3.4 Congenital Anomalies/Birth Defects

Any congenital anomaly/birth defect in a child born to a female patient or female partner of a male patient exposed to study drug should be classified as an SAE, recorded on the Adverse Event eCRF, and reported to the Sponsor within 24 hours after learning of the event (see Section 5.4.2).

5.4.4 REPORTING REQUIREMENTS FOR CARDIAC EVENTS RELATED TO CHF

The Investigator must report all cardiac events related to CHF (serious & non-serious) to the Sponsor within 24 hours after learning of the event, regardless of relationship to study drug.

5.5 FOLLOW-UP OF PATIENTS AFTER ADVERSE EVENTS

5.5.1 INVESTIGATOR FOLLOW-UP

The Investigator should follow each AE until the event has resolved to baseline grade or better, the event is assessed as stable by the Investigator, the patient is lost to follow up, or the patient withdraws consent. Every effort should be made to follow all SAEs until a final outcome can be reported.

During the study period, resolution of AEs (with dates) should be documented on the Adverse Event eCRF and in the patient's medical record to facilitate source data verification. If, after follow-up, return to baseline status or stabilization cannot be established, an explanation should be recorded on the Adverse Event eCRF.

All pregnancies reported during the study should be followed until pregnancy outcome. If the EDC system is not available at the time of pregnancy outcome, follow reporting instructions provided in Section 5.4.3.1.

After the 28 day period following the last dose of trastuzumab in an individual patient, AE follow-up will continue as follows:

Related AEs and SAEs will be followed until one of the following occurs:

- Resolved or improved to baseline state
- Investigator confirms that no further improvement can be expected
- Start of a new anti-cancer regimen
- Death

Unrelated severe or life threatening AEs and SAEs will be followed until one of the following occurs:

- Resolved or improved to baseline state
- Severity improved to Grade 2
- Investigator confirms that no further improvement can be expected
- Start of new anti-cancer regimen
- Death

Unrelated Grade 1 or Grade 2 AEs will be followed until 4 weeks after the last dose of study drug in an individual patient.

Cardiac AEs and SAEs: Follow until one of the following occurs:

- Resolved or improved to baseline state
- Investigator confirms that no further improvement can be expected
- Death
- Clinical or safety data will no longer be collected, or final database closure.

The final outcome of each adverse event must be recorded on the eCRF.

Follow-up of Abnormal Laboratory Test Values

In the event of medically significant unexplained abnormal laboratory test values, the tests should be repeated and followed up until they have returned to the normal range or baseline state and/or an adequate explanation of the abnormality is found. If a clear explanation is established it should be recorded on the eCRF.

5.5.2 SPONSOR FOLLOW-UP

For SAEs and pregnancies, the Sponsor or a designee may follow up by telephone, fax, electronic mail, and/or a monitoring visit to obtain additional case details and outcome information (e.g., from hospital discharge summaries, consultant reports, autopsy reports) in order to perform an independent medical assessment of the reported case.

5.6 POST-STUDY ADVERSE EVENTS

At the safety follow-up visit, the Investigator should instruct each patient to report to the Investigator any subsequent adverse events. The Sponsor should be notified if the Investigator becomes aware of any death or serious adverse event occurring at any time, after a patient has discontinued study participation, even after study closure, regardless of relationship to treatment of study drug. The investigator is not required to actively monitor patients once the study has ended.

The Sponsor should also be notified if the Investigator becomes aware of the development of cancer or a congenital anomaly/birth defect in a subsequently conceived offspring of a patient that participated in this study.

The Investigator should report these events to Roche Safety Risk Management on the Adverse Event eCRF. If the Adverse Event eCRF is no longer available, the Investigator should report

these events, **indefinitely**, directly to Roche Safety Risk Management via telephone (see "Protocol Administrative and Contact Information & List of Investigators").

5.7 EXPEDITED REPORTING TO HEALTH AUTHORITIES, INVESTIGATORS, INSTITUTIONAL REVIEW BOARDS, AND ETHICS COMMITTEES

To determine reporting requirements for single adverse event cases, the Sponsor will assess the expectedness of these events using the following reference documents:

- Trastuzumab Investigator's Brochure

The Sponsor will compare the severity of each event and the cumulative event frequency reported for the study with the severity and frequency reported in the applicable reference document.

Reporting requirements will also be based on the Investigator's assessment of causality and seriousness, with allowance for upgrading by the Sponsor as needed.

6. STATISTICAL CONSIDERATIONS AND ANALYSIS PLAN

The following is an outline of the statistical methodology that will be used to report and analyze this study. A more detailed description will be provided in a separate statistical analysis plan (SAP) that may include additional exploratory analysis not explicitly mentioned in the following sections.

- A descriptive analysis of the safety parameters will be performed.
- In addition, patient experience and overall satisfaction, patient reporting of symptoms and health care professional experience and satisfaction will be analyzed descriptively.

Details will be described in the statistical analysis plan that will be finalized before data base lock.

No formal hypothesis will be tested nonetheless, statistical tests will be applied and p-values calculated to explore specific aspects of the study material

Local safety data will be transferred to a global database and will be pooled for analysis. These data will also be analyzed in subgroups according to study design, patient population, and other relevant factors.

All safety summaries will be based on the safety population, defined as all recruited patients who receive at least one dose of study medication (trastuzumab).

Where it is stated that data will be summarized, unless alternative methods are given, the following will apply:

- continuous data will be summarized using: mean, median, range, standard deviation and standard error
- discrete data will be summarized using frequency counts and percentages

6.1 DETERMINATION OF SAMPLE SIZE

One hundred subjects are foreseen to be enrolled to assess the overall safety and tolerability of trastuzumab SC vial when administered at home. This sample size was not based on a formal calculation but mainly driven by feasibility and a reasonable width of a 95% confidence interval for the rate of AEs and SAEs. Based on an expected rate of AEs of 97% (23) and 100 subjects, a 95% confidence interval of 91% to 99% would be obtained. For an expected rate of SAEs of 21% (23) and 100 subjects, a 95% confidence interval ranging from 13% to 29% would be obtained (confidence intervals calculated using Clopper-Pearson methodology).

6.2 SUMMARIES OF CONDUCT OF STUDY

In order to assess the conduct of the study, major protocol violations will be summarized and listed. Such violations will be defined prior to the first reporting event but will include at least the following:

- Noncompliance with inclusion criteria
- Noncompliance with exclusion criteria
- Noncompliance with study treatment
- Use of disallowed concomitant medication

6.3 SUMMARIES OF BASELINE CHARACTERISTICS

All demography and baseline disease characteristics (collected at either the Screening or Baseline visits) will be summarized using the ITT population.

Summaries will include:

- Demography (age, gender)
- HER2 determination
- Medical History (including BC Disease History)
- Pregnancy Test Results
- Prior Medications

6.4 SAFETY ANALYSES

Safety endpoints of this study are:

- Adverse Events (AEs)
- Serious Adverse Events (SAEs)

- LVEF
- Exposure to study medication
- Duration of treatment
- Duration of follow-up
- Vital Signs, weight, height and ECOG
- Concomitant Medications
- Hematology and Serum Chemistry

All safety summaries will be based on the safety population.

AEs will be coded centrally by the Sponsor using the latest MedDRA dictionary and summarized by body system and preferred term.

Summaries will include frequency counts and percentages. For certain AEs (or groups of AEs) of interest, e.g. Cardiac AEs, 95% confidence intervals for incidences will be provided. Confidence intervals will be calculated using Clopper-Pearson methodology.

Summaries will include:

- The incidence of AEs and SAEs
    - overall
    - by severity using NCI CTCAE version 4.0
    - by relationship to study drug
- The incidence of AEs leading to premature discontinuation of study treatment
    - overall
    - by severity using NCI CTCAE version 4.0
    - by relationship to study drug
- The incidence of specific Cardiac AEs and SAEs
    - overall
    - by severity using NCI CTCAE version 4.0
    - by relationship to study drug

These adverse event summaries will be based on treatment emergent adverse events (those events starting on or after first study drug and within 28 days after last study drug). Similar summaries for the safety follow-up period (starting after 28 days after last study drug and continuing to study end) will be produced. AEs and SAEs occurring prior to first study drug will be listed only. AEs and SAEs starting in-hospital and in the home setting will be reported separately and combined

Cardiac monitoring (12-lead ECG and LVEF) will be summarized by cycle including change from baseline summaries where appropriate.

Exposure to study treatment (trastuzumab) will be summarized overall and by cycle. Summary measures will include:

- days on drug
- total daily dose
- cumulative dose

Duration of the treatment period will be summarized in both days and cycles.

Duration of safety follow-up will be summarized in days.

Vital signs will be summarized by cycle including change from baseline as appropriate.

Concomitant medications will be coded centrally by the Sponsor using the latest INN (International Nonproprietary Name) dictionary and summarized during the treatment phase by super class term and preferred term.>>

Anti-cancer treatments given to treat a recurrence will also be summarized during safety follow-up.

Hematology and serum chemistry results will be summarized by cycle including change from baseline as appropriate. For laboratory parameters where CTC grading is available, shift tables and change from baseline to worst on-treatment value will be produced.

The primary analysis will be undertaken once all patients have completed the study (treatment phase and 28-days safety follow-up visit). In addition to this primary analysis there will be annual interim analyses for safety reporting and presentation of safety and efficacy results. These annual reporting events will start 1 year after first patient's first visit and end when the last patient has finished follow-up.

6.5 PATIENT REPORTED OUTCOME ANALYSIS

The questionnaires that assess patient overall satisfaction and experience with the administration of trastuzumab SC vial in the hospital and at home will be analyzed descriptively by presenting frequencies and percentages for the different questions.

Patient reporting of symptoms using the MDASI will be analyzed descriptively at each time point the questionnaire was assessed and for each item separately using descriptive statistics like mean, standard deviation, median, interquartiles range and minimum and maximum values.

7. DATA COLLECTION AND MANAGEMENT

7.1 DATA QUALITY ASSURANCE

Data management will be performed by a CRO.

The Sponsor will supply eCRF specifications for this study. A contract research organization (CRO) will be responsible for data management of this study, including quality checking of the data. Data entered manually will be collected via EDC using eCRFs. Sites will be responsible for data entry into the EDC system. In the event of discrepant data, the CRO will request data clarification from the sites, which the sites will resolve electronically in the EDC system.

The CRO will produce a Data Quality Plan that describes the quality checking to be performed on the data.

The overall procedures for quality assurance of clinical study data are described in the Roche Standard Operational Procedures

Accurate and reliable data collection will be assured by verification and cross–check of the eCRFs against the investigator's records by the study monitor (source document verification and critical variables checks).

A comprehensive validation check program utilizing front-end checks in the eCRF will verify the data. Discrepancies and queries will be generated accordingly in the eCRF for online resolution by the investigator at the site.

The eCRF data will be reviewed on an ongoing basis for medical and scientific plausibility according to the Study Management Team Data Review Plan as described in the Data Quality Plan.

Roche will perform oversight of the data management of this study, including approval of the CRO's data management plans and specifications. Data will be periodically transferred electronically from the CRO to Roche, and Roche's standard procedures will be used to handle and process the electronic transfer of these data.

eCRFs and correction documentation will be maintained in the EDC system's audit trail. System backups for data stored at the CRO and records retention for the study data will be consistent with the CRO's standard procedures.

Roche will ensure the quality of safety and efficacy data to be transferred to the global database.

7.2 ELECTRONIC CASE REPORT FORMS

eCRFs are to be completed using a Sponsor-designated EDC system. Sites will receive training and a have access to a manual for appropriate eCRF completion. eCRFs will be submitted electronically to Roche and should be handled in accordance with instructions from the Sponsor.

All eCRFs should be completed by designated, trained site staff. eCRFs should be reviewed and electronically signed and dated by the Investigator or a designee.

At the end of the study, the Investigator will receive patient data for his or her site in a readable format on a compact disc that must be kept with the study records. Acknowledgement of receipt of the compact disc is required.

7.3 PATIENT-REPORTED OUTCOME DATA

PRO data will be collected on paper. During the in hospital part of the study the questionnaires will be provided to the patient by the study nurse or other study-related personnel. During the at home period the patient will receive a patient diary in which all planned visit dates will be marked. This diary will also contain all questionnaires to be completed by the patient while he or she is been treated at home.

Patients will complete the questionnaires at defined time points during the study. The nurse administering trastuzumab subcutaneous vial at home, will remind the patient of the timing for each questionnaires to be completed. The completed questionnaires will be handed by the patient to the site at the first visit following the date of completion of the respective questionnaire. The study site personnel will transfer the PRO data into the eCRF.

Once the study is complete, the PRO data, audit trail, and trial and system documentation will be archived. The Investigator will receive patient data for the site in both human- and machine-readable formats on an archival-quality compact disc that must be kept with the study records as source data. Acknowledgement of receipt of the compact disc is required. In addition, the Sponsor will receive all patient data in a machine-readable format on a compact disc.

7.4 SOURCE DATA DOCUMENTATION

Study monitors will perform ongoing source data verification to confirm that critical protocol data (i.e., source data) entered into the eCRFs by authorized site personnel are accurate, complete, and verifiable from source documents.

Source documents (paper or electronic) are those in which patient data are recorded and documented for the first time. They include, but are not limited to, hospital records, clinical and office charts, laboratory notes, memoranda, patient-reported outcomes, evaluation checklists, pharmacy dispensing records, recorded data from automated instruments, copies of transcriptions that are certified after verification as being accurate and complete, microfiche, photographic negatives, microfilm or magnetic media, X-rays, patient files, and records kept at pharmacies, laboratories, and medico-technical departments involved in a clinical trial.

Before study initiation, the types of source documents that are to be generated will be clearly defined in the Trial Monitoring Plan. This includes any protocol data to be entered directly into the eCRFs (i.e., no prior written or electronic record of the data) and considered source data.

Source documents that are required to verify the validity and completeness of data entered into the eCRFs must not be obliterated or destroyed and must be retained per the policy for retention of records described in Section 7.6.

To facilitate source data verification, the Investigators and institutions must provide the Sponsor direct access to applicable source documents and reports for trial-related monitoring, Sponsor audits, and IRB/EC review. The investigational site must also allow inspection by applicable health authorities.

7.5 USE OF COMPUTERIZED SYSTEMS

When clinical observations are entered directly into an investigational site's computerized medical record system (i.e., in lieu of original hardcopy records), the electronic record can serve as the source document if the system has been validated in accordance with health authority requirements pertaining to computerized systems used in clinical research. An acceptable computerized data collection system allows preservation of the original entry of data. If original data are modified, the system should maintain a viewable audit trail that shows the original data as well as the reason for the change, name of the person making the change, and date of the change.

7.6 RETENTION OF RECORDS

Records and documents pertaining to the conduct of this study and the distribution of IMP, including eCRFs, ePRO data, Informed Consent Forms, laboratory test results, and medication inventory records, must be retained by the Principal Investigator for at least 15 years after completion or discontinuation of the study, or for the length of time required by relevant national or local health authorities, whichever is longer. After that period of time, the documents may be destroyed, subject to local regulations.

No records may be disposed of without the written approval of the Sponsor. Written notification should be provided to the Sponsor prior to transferring any records to another party or moving them to another location.

8. ETHICAL CONSIDERATIONS

8.1 COMPLIANCE WITH LAWS AND REGULATIONS

This study will be conducted in full conformance with the ICH E6 guideline for Good Clinical Practice and the principles of the Declaration of Helsinki, or the laws and regulations of the country in which the research is conducted, whichever affords the greater protection to the individual. The study will comply with the requirements of the ICH E2A guideline (Clinical Safety Data Management: Definitions and Standards for Expedited Reporting). Studies conducted in the United States or under a U.S. Investigational New Drug (IND) application will comply with U.S. FDA regulations and applicable local, state, and federal laws. Studies conducted in the EU/EEA will comply with the EU Clinical Trial Directive (2001/20/EC).

8.2 INFORMED CONSENT

Roche's sample Informed Consent Form (and ancillary sample Informed Consent Forms such as a Child's Assent or Caregiver's Informed Consent Form, if applicable) will be provided to each site. If applicable, it will be provided in a certified translation of the local language. The Sponsor or its designee must review and approve any proposed deviations from Roche's sample Informed Consent Forms or any alternate consent forms proposed by the site (collectively, the "Consent Forms") before IRB/EC submission. The final IRB/EC-approved Consent Forms must be provided to Roche for health authority submission purposes according to local requirements.

The Consent Forms must be signed and dated by the patient or the patient's legally authorized representative before his or her participation in the study. The case history or clinical records for

each patient shall document the informed consent process and that written informed consent was obtained prior to participation in the study.

The Consent Forms should be revised whenever there are changes to study procedures or when new information becomes available that may affect the willingness of the patient to participate. The final revised IRB/EC-approved Consent Forms must be provided to the Sponsor for health authority submission purposes.

Patients must be re-consented to the most current version of the Consent Forms (or to a significant new information/findings addendum in accordance with applicable laws and IRB/EC policy) during their participation in the study. For any updated or revised Consent Forms, the case history or clinical records for each patient shall document the informed consent process and that written informed consent was obtained using the updated/revised Consent Forms for continued participation in the study.

A copy of each signed Consent Form must be provided to the patient or the patient's legally authorized representative. All signed and dated Consent Forms must remain in each patient's study file or in the site file and must be available for verification by study monitors at any time.

8.3 INSTITUTIONAL REVIEW BOARD OR ETHICS COMMITTEE

This protocol, the Informed Consent Forms, any information to be given to the patient, and relevant supporting information must be submitted to the EC by the Principal Investigator and reviewed and approved by the EC before the study is initiated. In addition, any patient recruitment materials must be approved by the EC.

The Principal Investigator is responsible for providing written summaries of the status of the study to the EC annually or more frequently in accordance with the requirements, policies, and procedures established by the EC. Investigators are also responsible for promptly informing the EC of any protocol amendments (see Section 9.5).

In addition to the requirements for reporting all AEs to the Sponsor, Investigators must comply with requirements for reporting SAEs to the local health authority and EC. Investigators may receive written IND safety reports or other safety-related communications from the Sponsor. Investigators are responsible for ensuring that such reports are reviewed and processed in accordance with health authority requirements and the policies and procedures established by their EC, and archived in the site's study file.

8.4 CONFIDENTIALITY

The Sponsor maintains confidentiality standards by coding each patient enrolled in the study through assignment of a unique patient identification number. This means that patient names are not included in data sets that are transmitted to any the Sponsor location.

Patient medical information obtained by this study is confidential and may only be disclosed to third parties as permitted by the Informed Consent Form (or separate authorization for use and

disclosure of personal health information) signed by the patient, unless permitted or required by law.

Medical information may be given to a patient's personal physician or other appropriate medical personnel responsible for the patient's welfare, for treatment purposes.

Data generated by this study must be available for inspection upon request by representatives of the U.S. FDA and other national and local health authorities, the Sponsor monitors, representatives, and collaborators, and the EC for each study site, as appropriate.

8.5 FINANCIAL DISCLOSURE

Investigators will provide the Sponsor with sufficient, accurate financial information in accordance with local regulations to allow the Sponsor to submit complete and accurate financial certification or disclosure statements to the appropriate health authorities. Investigators are responsible for providing information on financial interests during the course of the study and for 1 year after completion of the study.

9. STUDY DOCUMENTATION, MONITORING, AND ADMINISTRATION

9.1 STUDY DOCUMENTATION

The Investigator must maintain adequate and accurate records to enable the conduct of the study to be fully documented, including but not limited to the protocol, protocol amendments, Informed Consent Forms, and documentation of EC and governmental approval. In addition, at the end of the study, the Investigator will receive the patient data, which includes an audit trail containing a complete record of all changes to data.

9.2 SITE INSPECTIONS

Site visits will be conducted by the Sponsor or an authorized representative for inspection of study data, patients' medical records, and eCRFs. The Investigator will permit national and local health authorities, The Sponsor monitors, representatives, and collaborators, and the ECs to inspect facilities and records relevant to this study.

9.3 ADMINISTRATIVE STRUCTURE

The study will have an internal Data Monitoring Committee.

9.4 PUBLICATION OF DATA AND PROTECTION OF TRADE SECRETS

The results of this study may be published or presented at scientific meetings. If this is foreseen, the Investigator agrees to submit all manuscripts or abstracts to the Sponsor prior to submission. This allows the Sponsor to protect proprietary information and to provide comments based on information from other studies that may not yet be available to the Investigator.

The Sponsor will comply with the requirements for publication of study results. In accordance with standard editorial and ethical practice, the Sponsor will generally support publication of multicenter trials only in their entirety and not as individual center data. In this case, a coordinating Investigator will be designated by mutual agreement.

Authorship will be determined by mutual agreement and in line with International Committee of Medical Journal Editors authorship requirements. Any formal publication of the study in which contribution of the Sponsor personnel exceeded that of conventional monitoring will be considered as a joint publication by the Investigator and the appropriate the Sponsor personnel.

Any inventions and resulting patents, improvements, and/or know-how originating from the use of data from this study will become and remain the exclusive and unburdened property of the Sponsor, except where agreed otherwise.

9.5 PROTOCOL AMENDMENTS

Any protocol amendments will be prepared by the Sponsor. Protocol amendments will be submitted to the IRB/EC and to regulatory authorities in accordance with local regulatory requirements.

Approval must be obtained from the IRB/EC and regulatory authorities (as locally required) before implementation of any changes, except for changes necessary to eliminate an immediate hazard to patients or changes that involve logistical or administrative aspects only (e.g., change in Medical Monitor or contact information).

10. REFERENCES

1. Aebi S, Davidson T, Gruber G, Cardoso F; ESMO Guidelines Working Group. Primary breast cancer: ESMO Clinical Practice Guidelines for diagnosis, treatment and follow-up. Ann Oncol. 2011; 22 Suppl 6:vi12-24.
2. Baselga J. Treatment of HER2-overexpressing breast cancer. Ann Oncol 2010; Suppl 7:vii36-40.
3. Baselga J, Cortés J, Kim SB, et al. Pertuzumab plus Trastuzumab plus Docetaxel for Metastatic Breast Cancer. N Engl J Med. 2012; 366(2):109-19.
4. Chang J, Clark GM, Allred DC, et al. Survival of patients with metastatic breast cancer. Importance of prognostic markers of the primary tumor. Cancer 2003; 97:545-53.
5. Cho HS, Mason K, Ramyar KX, et al. Structure of the extracellular region of HER2 alone and in complex with the Herceptin Fab. Nature 2003; 421:756-60.
6. Coates A, et al. On the receiving end-patient perception of the side-effects of cancer chemotherapy. Eur J Cancer Clin Oncol 1983; 19: 203-208.
7. Cobleigh MA, Vogel CL, Tripathy D, et al. Multinational study of the efficacy and safety of humanized anti-HER2 monoclonal antibody in women who have HER2-overexpressing metastatic breast cancer that has progressed after chemotherapy for metastatic disease. J Clin Oncol. 1999; 17:2639-48.
8. Colozza M, de Azambuja E, Cardoso F, et al. Breast cancer: achievements in adjuvant systemic therapies in the pre-genomic era. Oncologist 2006; 11:111-25.
9. Crowe JP, Patrick RJ, Rybicki LA, et al. A data model to predict HER2 status in breast cancer based on the clinical and pathologic profiles of a large patient population at a single institution. Breast 2006; 15:728-35.
10. Dawood S, Broglio K, Buzdar AU, Hortobagyi GN, Giordano SH. Prognosis of women with metastatic breast cancer by HER2 status and trastuzumab treatment: an institutional-based review. J Clin Oncol. 2010; 28:92-8.
11. Ewer MS, O'Shaughnessy JA. Cardiac toxicity of trastuzumab-related regimens in HER2-overexpressing breast cancer. Clin Breast Cancer. 2007; 7:600-7.
12. Ferlay J, Autier P, Boniol M, et al. Estimates of the cancer incidence and mortality in Europe in 2006. Annals of Oncology 2007; 18:581-592
13. F. Hoffmann-La Roche Ltd. FDA Approves Perjeta (Pertuzumab) for People With HER2-Positive Metastatic Breast Cancer. http://www.roche.com/media/media_releases/med-cor-2012-06-11.htm. Press release 2012 June 11 (Accessed: 2012 August 30).
14. Franklin MC, Carey KD, Vajdos FF, et al. Insights into ErbB signaling from the structure of the ErbB2-pertuzumab complex. Cancer Cell 2004; 5:317−28.
15. Frost GI. Recombinant human hyaluronidase (rHuPH20): an enabling platform for subcutaneous drug and fluid administration. Expert Opin. Drug Deliv 2007; 4:427-440.

16. Garnock-Jones KP, Keating GM, Scott LJ. Trastuzumab: A review of its use as adjuvant treatment in human epidermal growth factor receptor 2 (HER2)-positive early breast cancer. Drugs. 2010; 70:215-39.

17. Geyer CE, Forster J, Lindquist D, et al. Lapatinib plus capecitabine for HER2-positive advanced breast cancer. N Engl J Med 2006; 355:2733−2743.

18. Gianni L, Dafni U, Gelber RD, et al. Treatment with trastuzumab for 1 year after adjuvant chemotherapy in patients with HER2-positive early breast cancer: a 4-year follow-up of a randomised controlled trial. Lancet Oncol 2011; 12:236-44.

19. Goldhirsch A, Wood WC, Coates AS, Gelber RD, Thürlimann B, Senn HJ; Panel members. Strategies for subtypes-dealing with the diversity of breast cancer: highlights of the St. Gallen International Expert Consensus on the Primary Therapy of Early Breast Cancer 2011. Ann Oncol. 2011; 22:1736-47.

20. Gschwind A, Fischer OM, Ullrich A. The discovery of receptor tyrosine kinases: targets for cancer therapy. Nat Rev Cancer 2004; 4:361-70.

21. Harris J. Clinical Review of NDA 21-640. FDA Report: December 1 2003:1-18. Http://www.fda.gov/cder/foi/nda/2004/21-640_Vitrase_Medr.pdf (accessed 20051210).

22. Hylenex: Full Prescribing Information; Administrative Review of NDA 21-859 http://www.fda.gov/cder/foi/label/2005/021859lbl.pdf [Accessed 2008 April 18).

23. Ismael G, Hegg R, Muehlbauer S, et al. Lancet Oncology. 2012; Subcutaneous versus intravenous administration of (neo)adjuvant trastuzumab in patients with HER2-positive, clinical stage I-III breast cancer (HannaH study): a phase 3, open-label, multicentre, randomised trial. Lancet Oncol. 2012 Aug 8. [Epub ahead of print].

24. Jackisch C, Dank M, Frasci G. Additional safety results of HannaH: A Phase III randomised, open-label, international study of the subcutaneous formulation of trastuzumab (H) in HER2-positive early breast cancer patients. ESMO 2012 (abstract)

25. Jahanzeb M. Adjuvant trastuzumab therapy for HER2-positive breast cancer. Clin Breast Cancer 2008; 8:324-33.

26. Joensuu H, Bono P, Kataja V, et al. Fluorouracil, epirubicin, and cyclophosphamide with either docetaxel or vinorelbine, with or without trastuzumab, as adjuvant treatments of breast cancer: final results of the FinHer Trial. J Clin Oncol 2009 27:5685-92.

27. Khatcheressian JL, Wolff AC, Smith TJ, et al. for the American Society of Clinical Oncology. American Society of Clinical Oncology 2006 update of the breast cancer follow-up and management guidelines in the adjuvant setting. J Clin Oncol 2006; 24:5091-7.

28. Lund MJ, Butler EN, Hair BY, et al. Age/race differences in HER2 testing and in incidence rates for breast cancer triple subtypes: a population-based study and first report. Cancer 2010; 116:2549-59.

29. Lundin J, Kimby E, Björkholm M, et al. Phase II trial of subcutaneous anti-CD52 monoclonal antibody alemtuzumab (Campath-1H) as first line treatment for patients with B-cell chronic lymphocytic leukemia (B-CLL). Blood 2002; 100:768-73.

30. Ménard S et al. HER2 as a prognostic factor in breast cancer. Oncology 2001; 61(Suppl. 2):67–72.

31. Moasser MM. The oncogene HER2: Its signaling and transforming functions and its role in human cancer pathogenesis. Oncogene 2007; 26:6469-87.

32. National Comprehensive Cancer Network. NCCN clinical practice guidelines in oncology: Breast cancer, Version 2.2012 [online]. Available from URL: http://www.nccn.org/professionals/physician_gls/pdf/breast.pdf [Accessed 2012 July 18]

33. Oken MM, Creech RH, Tormey DC, et al. Toxicity and response criteria of the Eastern Cooperative Oncology Group. Am J Clin Oncol. 1982; 5:649-55.

34. Pauletti G, Dandekar S, Rong H, et al. Assessment of methods for tissue-based detection of the HER-2neu alteration in human breast cancer: a direct comparison of fluorescent in-situ hybridization and immunohistochemistry. J Clin Oncol 2000; 18:3651−64.

35. Perez EA, Romond EH, Suman VJ et al. Updated results of the combined analysis of NCCTG N9831 and NSABP B-31. Adjuvant chemotherapy with or without trastuzumab (H) in patients with HER2-positive breast cancer. Proc ASCO 2007, abstract 512.

36. Perez EA, Suman VJ, Davidson NE, et al. Results of chemotherapy alone, with sequential or concurrent addition of 52 weeks of trastuzumab in the NCCTG N9831 HER2-positive adjuvant breast cancer trial. San Antonio Breast Cancer Symposium 2009 abstract 80.

37. Perez EA, Romond EH, Suman VJ, et al. Four-year follow-up of trastuzumab plus adjuvant chemotherapy for operable human epidermal growth factor receptor 2-positive breast cancer: Joint Analysis of Data From NCCTG N9831 and NSABP B-31. J Clin Oncol 2011; 29:3366-73.

38. Piccart-Gebhart MJ, Procter M, Leyland-Jones B et al. Trastuzumab after adjuvant chemotherapy in HER2-positive breast cancer. N Engl J Med 2005; 353:1659-72.

39. Pivot X, Semiglazov V, Chen SC et al. Subcutaneous injection of trastuzumab – analysis of administration time and injection site reactions. ESMO 2012 (abstract)

40. Press MF, Sauter G, Bernstein L, et al. Diagnostic evaluation of HER-2 as a molecular target: an assessment of accuracy and reproducibility of laboratory testing in large, prospective, randomized clinical trials. Clin Cancer Res. 2005; 11:6598–607.

41. Procter M, Suter TM, de Azambuja E, et al. Longer-term assessment of trastuzumab-related cardiac adverse events in the Herceptin Adjuvant (HERA) trial. J Clin Oncol 2010; 28:3422-8.

42. Rastogi P, Jeong J, Geyer CE. Five year update of cardiac dysfunction on NSABP B-31, a randomized trial of sequential doxorubicin/cyclophosphamide (AC)→paclitaxel (T) vs. AC→T with trastuzumab(H). ASCO Annual Meeting 2007 abstract LBA513.

43. Romond EH, Perez EA, Bryant J, et al. Trastuzumab plus adjuvant chemotherapy for operable HER2-positive breast cancer. N Engl J Med 2005; 353:1673−84.

44. Ross JS et al. The HER-2/neu oncogene in breast cancer: Prognostic factor, predictive factor and target for therapy. Stem Cells 1998; 16:413-428

45. Ross JS, Slodkowska EA, Symmans WF, et al. The HER-2 receptor and breast cancer: ten years of targeted anti-HER-2 therapy and personalized medicine. Oncologist 2009; 14:320-68.

46. Russell SD, Blackwell KL, Lawrence J, et al. Independent adjudication of symptomatic heart failure with the use of doxorubicin and cyclophosphamide followed by trastuzumab adjuvant therapy: a combined review of cardiac data from the National Surgical Adjuvant breast and Bowel Project B-31 and the North Central Cancer Treatment Group N9831 clinical trials. J Clin Oncol. 2010; 28:3416-21.

47. Sant M, Allemani C, Capocaccia R, et al. Stage at diagnosis is a key explanation of differences in breast cancer survival across Europe. Int J Cancer 2003; 106: 416-22.

48. Schechter AL, Stern DF, Vaidyanathan L, et al. The neu oncogene: An erb-B-related gene encoding a 185,000-Mr tumour antigen. Nature 1984; 312:513-6.

49. Sjögren S, Inganäs M, Lindgren A, et al. Prognostic and predictive value of c-erbB-2 overexpression in primary breast cancer, alone and in combination with other prognostic markers. J Clin Oncol 1998 Feb;16(2):462-9.

50. Slamon DJ, Clark GM, Wong SG et al. Human breast cancer: correlation of relapse and survival with amplification of the HER-2/neu oncogene, Science 1987; 235:177-82

51. Slamon DJ, Godolphin W, Jones LA, et al. Studies of the HER-2/neu proto-oncogene in human breast and ovarian cancer. Science 1989; 244:707-12Slamon DJ, Leyland-Jones B, Shak S, et al. Use of chemotherapy plus a monoclonal antibody against HER-2 for metastatic breast cancer. N Engl J Med 2001; 344:783−92.

52. Slamon D, Eiermann W, Robert N, et al. BCIRG-006 Phase III trial comparing AC→T with AC→TH and with TCH in the adjuvant treatment of HER2-amplified early breast cancer patients: third planned efficacy analysis. San Antonio Breast Cancer Symposium 2009 abstract 62.

53. Slamon D, Eiermann W, Robert N, et al. (Breast Cancer International Research Group). Adjuvant trastuzumab in HER2-positive breast cancer. N Engl J Med. 2011; 365:1273-83.

54. Smith I, Procter M, Gelber RD, et al. 2-year follow-up of trastuzumab after adjuvant chemotherapy in HER2-positive breast cancer: a randomised controlled trial. Lancet 2007; 369:29-36.

55. Spielmann M, Roche H, Delozier T, et al. Trastuzumab for patients with axillary-node-positive breast cancer: results of the FNCLCC-PACS 04 trial. J Clin Oncol 2009; 27:6129-34.

56. Sundaresan S, Penuel E, Sliwkowski MX. The biology of human epidermal growth factor receptor 2. Curr Oncol Rep. 1999; 1:16-22.

57. Tan-Chiu E, Yothers G, Romond E, et al. Assessment of cardiac dysfunction in a randomized trial comparing doxorubicin and cyclophosphamide followed by paclitaxel, with or without trastuzumab as adjuvant therapy in node-positive, human epidermal growth factor receptor 2-overexpressing breast cancer: NSABP B-31. J. Clin. Oncol 2005; 23:7811-9.

58. Untch M. Targeted Therapy for Early and Locally Advanced Breast Cancer. Breast Care (Basel) 2010; 5:144-52.

59. Verma S, Lavasani S, Mackey J, et al. Optimizing the management of HER2-positive early breast cancer: the clinical reality. Curr Oncol 2010; 17:20-33.

60. Vogel C, Cobleigh M, Tripathy D, et al. Efficacy and safety of trastuzumab as a single agent in first-line treatment of HER2-overexpressing metastatic breast cancer. J Clin Oncol 2002; 20:719-26.

61. Wynne C, Harvey V, Schwabe C, Waaka D, McIntyre C, Bittner B. The development of a formulation of trastuzumab for subcutaneous administration. European Society for Medical Oncology Congress 2010 (Abs 218PD).

62. Wynne CJ, Wakka DS, Ellis-Pegler RB, et al. Comparative pharmacokinetics of trastuzumab subcutaneous formulation administered using a proprietary single-use injection device, or manually using a syringe. European Society for Medical Oncology Congress 2012 (Abs submitted).

63. Yarden Y, Sliwkowski M. Untangling the ErbB signalling network. Nat Rev Mol Cell Biol 2001; 2:127-37

Appendix 1
Schedule of Assessments

	Screening	Baseline	Treatment period<br>(cycles trastuzumab)				Safety FU<br>Visit (o)	Follow-up visits
(Treatment cycle)	Day -28 to 1	Day -7 to 1	Period 1 – Hospital<br>[Trastuzumab IV]<br>(Cycle 7-9)<br>All visits within +/- 3 days of scheduled treatment day	Period 2 – Hospital<br>[Trastuzumab SC]<br>(Cycle 10-12)<br>All visits within +/- 3 days of scheduled treatment day	Period 3 – Home<br>[Trastuzumab SC]<br>(Cycle 13-18)		4 weeks after last study treatment<br>+7 days	Continuing for up to 24 months after treatment cessation<br>+/- 15 days
					(Cycle 13-15)<br>All visits within +/- 3 days of schedu-led treat-ment day	(Cycle 16-18)<br>All visits within +/- 3 days of sche-duled treat-ment		

												day				
Explain study and obtain signed informed consent (a)	x															
Demographics and medical history (b,c)	x															
Review inclusion/exclusion criteria	x															
Vital signs and physical assessment (d)	x		X	X	X	X	X	X	X	X	X	X	X	X	x	
Weight, height (e)	x		X	X	X	X	X	X	X	X	X	X	X	X	x	
ECOG performance status (v)	x		X(v)			x(v)			X(v)						X	
Cardiac monitoring:<br>12-lead ECG (f)<br>LVEF (f)<br>Signs/symptoms (f)	 X<br>X*<br>X		Cardiac safety assessments will be performed approximately 3-monthly during treatment period.												 X<br>X<br>X	Cardiac assessments at 6, 12, 18 and 24 months following treatment cessation
Pregnancy test (g)		X														

Hematology and biochemistry (h)	x		X(h)	X(h) (If not done during first part of treatment period)	X (h)	X (h) (If not done during first part of treatment period)	X	
Routine breast cancer follow-up (i)	Assessments as per institutional practice or ASCO adjuvant follow-up guidelines 2006 to be reported 6-monthly (i)							
AEs and SAEs (j)	x	X	X	X	X	X	X	X
Past and Current Concomitant medications (k)	x	X	X	X	X	X	X	X
Trastuzumab IV (l)			x					
Trastuzumab SC (m)				X	X	X		
PRO MDASI (p)			X	X	X	X		
PRO PSQ1 (q)					X			
PRO PSQ2 (r)						X		
HCPEX-1 (s)				X				

PEX-P1 (t)				X				
PEX-P2 (u)							X	

Treatment period 1: The first 3 administrations of trastuzumab will be performed intravenously at 6mg/kg in the hospital (cycle 7 to 9).

Treatment period 2: The next 3 administrations of trastuzumab will be performed subcutaneously in the hospital. Trastuzumab SC vial will be administered by a HCP at a fixed dose of 600 mg, into the thigh over a period of up to 5 minutes, using conventional handheld syringes (cycle 10 to 12).

Treatment period 3: The next 6 administrations of trastuzumab will be performed subcutaneously at home. Trastuzumab SC vial will be administered by a HCP at a fixed dose of 600 mg, into the thigh over a period of up to 5 minutes, using conventional handheld syringes (cycle 13 to 18).

AE = adverse event, ASCO = American Society of Clinical Oncology, ECG = electrocardiogram, ECOG = Eastern Cooperative Oncology Group, LPLV = last patient last visit, LVEF = left ventricular ejection fraction, PK = pharmacokinetic, SAE = serious adverse event, SC = subcutaneous

First dose of drug = study cycle 1, Day 1. A treatment period is approximately 3 months in duration, i.e. 4 treatment cycles. Assessments can also be performed more frequently if clinically indicated.

Notes

[a] Written Informed Consent must be obtained before any study-specific assessments or procedures are performed

[b] Demographic data including date of birth, gender, and self-reported ethnic origin where permitted by local regulations

[c] Breast cancer disease history, including ER/PgR status, prior anticancer treatments, current medications and symptoms

[d] Vital signs and physical assessment including pulse, blood pressure, and body temperature

[e] Weight is measured for at baseline, at every treatment cycle, at the Safety Follow-up visit, and at each visit during follow-up period. Height is only measured at screening

[f] LVEF assessments by either ECHO or MUGA scan: the same imaging technique should be used per patient throughout the study. *The screening LVEF assessment should be performed within 3 months prior to the first trastuzumab IV administration. To be eligible for participation in this study, patients must have a baseline LVEF ≥ 50% for eBC patients. Cardiac safety assessments will be performed within 3 months prior to the

first trastuzumab SC vial administration and approximately 3-monthly during further trastuzumab SC vial treatment at the Safety Follow-up visit (LVEF assessment may occur prior to the day of the Safety Follow-up visit if clinically indicated) and then at 6, 12, 18 and 24 months after treatment cessation and immediately in case of cardiac failure.
[g] Applicable to women of childbearing potential (premenopausal or less than 12 months of amenorrhea post-menopause, and who have not undergone surgical sterilization); a serum pregnancy test needs to be completed within 7 days prior to the first dose of study treatment. Additional pregnancy testing should be completed as clinically indicated for the duration of study treatment and until at least 7 months after the last dose of study treatment. Any positive urine pregnancy test needs to be confirmed by serum pregnancy test
[h] Patients will undergo 4 lab evaluations: one at screening, one during hospital treatment period, one during at home treatment period and one during first safety follow-up visit. Choice of cycle within treatment period is upon discretion of the investigator. Hematology: hemoglobin, white blood cells (WBC) and differential, absolute neutrophil count (ANC), platelet count. Biochemistry: creatinine, urea (BUN), SGPT (ALT), SGOT (AST), total bilirubin, alkaline phosphatase, albumin, sodium, potassium and calcium. Additional hematology and biochemistry tests may be performed as per institutional practice, but these data will not be collected
[i] American Society of Clinical Oncology (ASCO) 2006 Guideline for Breast Cancer Follow-up in the adjuvant setting (Khatcheressian et al. 2006). In brief:History/physical examination - every 3 to 6 months for the first 3 years after primary therapy; every 6 to 12 months for years 4 and 5; then annually.Mammography - first post-treatment mammogram 1 year after the initial mammogram that leads to diagnosis, but no earlier than 6 months after definitive radiation therapy. Subsequent mammograms should be obtained as indicated for surveillance of abnormalities.Pelvic examination - regular gynecologic follow-up is recommended for all women. Patients who receive tamoxifen should be advised to report any vaginal bleeding to their physicians. The following are not recommended for routine surveillance: Routine blood tests (full blood counts and liver function tests), imaging studies (chest x-ray, bone scans, liver ultrasound, CT scans, FDG-PET scans, and breast MRI), tumor marker assessments (CA 15-3, CA 27.29, and CEA)[j] After informed consent, but prior to initiation of study treatment, only SAEs caused by a protocol-mandated intervention will be collected. All treatment-emergent AEs occurring until 4 weeks after the last SC administration of trastuzumab will be recorded in the eCRF, irrespective of the type of event and drug-event relationship. From 4 weeks to 6 months after the last study drug administration, related AEs, related/unrelated SAEs and cardiac AEs should be reported, except for pregnancy that needs to be reported to 7 months after the last study drug administration. From 6 months after the last study drug administration until the end of the follow-up period only related AEs/SAEs and cardiac AEs should be reported. NCI-CTCAE version 4 should be used for AE coding
[k] All concomitant medication will be recorded between the Screening and the Safety Follow-up visits. Thereafter, only anti-cancer treatments given to treat a recurrence will be reported
[l] Trastuzumab IV is administered intravenously at 6mg/kg for the subsequent doses, every 3 weeks for the first 3 cycles (cycle 7 to 9)[m] Trastuzumab is administered subcutaneously at a fixed dose of 600 mg, 3-weekly for a total of 9 cycels (cycle 10-18) (regardless of body weight)
[o] Patients will undergo a Safety Follow-up visit 4 weeks after their last dose of study treatment
(p) Patient reporting of symptoms using the MD Anderson Symptom Inventory (MDASI, appendix 4):Patients will be asked to rate the severity of 13 core items on a scale from 0 to 10. Patients will be asked to complete the MDASI 4 times: Prior to the first dose trastuzumab IV = prior to cycle 7, prior to the first dose trastuzumab SC vial = prior to cycle 10, prior to the first dose trastuzumab SC vial administered at home = prior to cycle 13 and prior to the fourth dose trastuzumab SC vial administered at home = prior to cycle 16.

(q) Patient experience with the treatment provided during the in-hospital part of the study: Patients will be asked to complete a questionnaire (PSQ1, appendix 6) related to the quality of care provided during trastuzumab administration in the hospital prior to the first dose trastuzumab SC vial administered at home = prior to cycle13.(r) Patient experience with the treatment provided during the at home part of the study: Patients will be asked to complete a questionnaire (PSQ2, appendix 6) related to the quality of care provided during trastuzumab SC vial administration at home prior to the fifth dose trastuzumab SC vial administered at home = prior to cycle 17.

(s) HCP overall satisfaction and perceived time savings with trastuzumab SC vial in the hospital will be assessed by a Health Care Professional Questionnaire (HCPEX-1, appendix 5): The HCPEX-1 questionnaire will be completed by a HCP administering trastuzumab IV and SC vial in the hospital after at least 3 patients completed the in hospital part of the study (3 patients must have completed treatment period 1 and 2)

(t) Patient experience with trastuzumab IV and SC vial administered in the hospital.

Patients will be interviewed by a HCP to complete the PEX-P1 (appendix 7) prior to the third dose trastuzumab SC = prior to cycle 12.

(u) Patient experience with trastuzumab SC vial administered at home. Patients will be interviewed by a HCP to complete the PEX-P2 (appendix 7) at the safety follow-up visit, following 4 weeks after the last trastuzumab SC vial administration at home.

(v) Patients will undergo 5 ECOG measurements: one at screening , one per treatment period and one during first safety follow-up visit. Choice of cycle within treatment period is upon discretion of the investigator.

Appendix 2
Algorithm for continuation and discontinuation of trastuzumab SC or IV based on LVEF assessment in asymptomatic patients

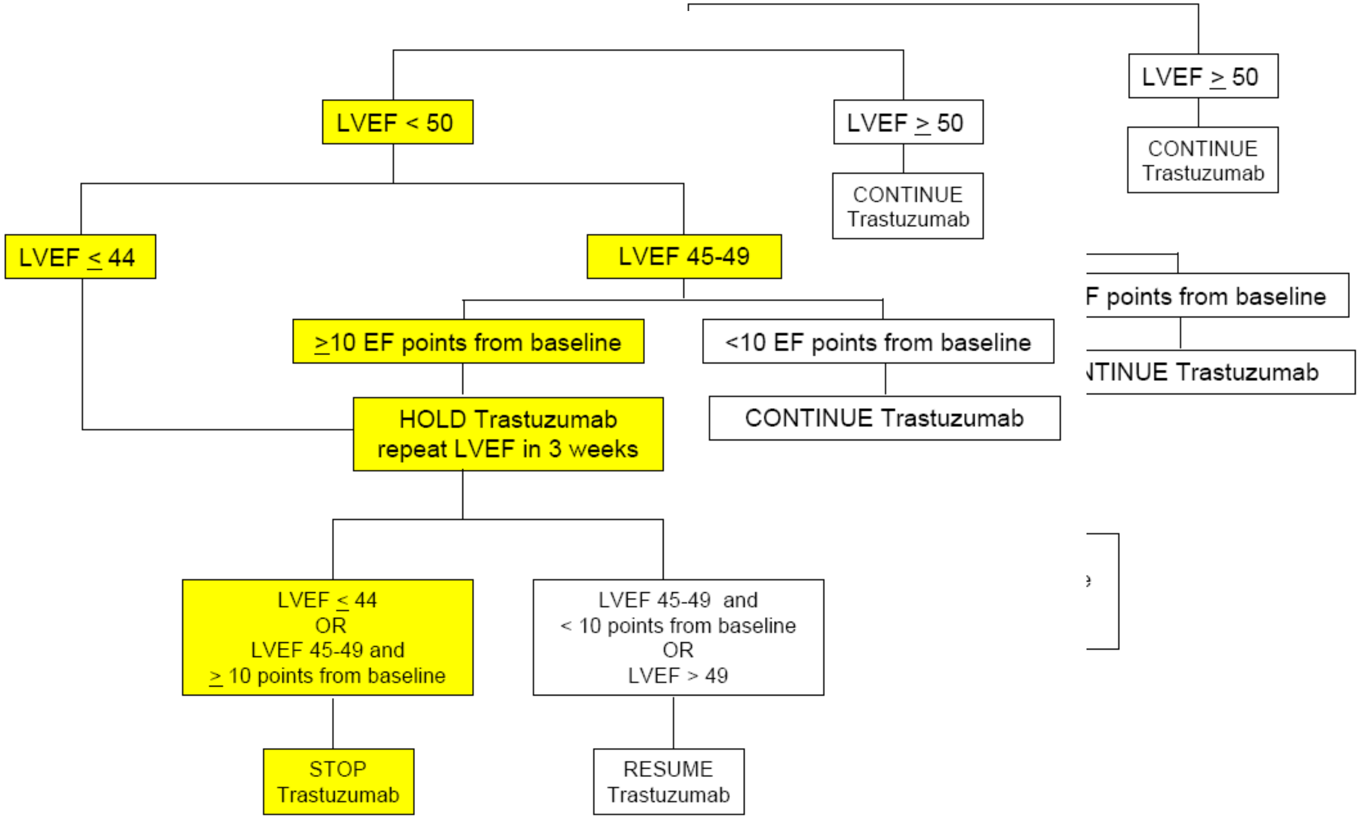

Appendix 3
NYHA Classification and Left Ventricular Systolic Dysfunction NCI CTCAE version 4.0 Grading

Class I	Patients with cardiac disease but without resulting limitations of physical activity. Ordinary physical activity does not cause undue fatigue, palpitation, dyspnea or angina pain.
Class II	Patients with cardiac disease resulting in slight limitations of physical activity. They are comfortable at rest. Ordinary physical activity results in fatigue, palpitation, dyspnea or anginal pain.
Class III	Patients with cardiac disease resulting in marked limitations of physical activity. They are comfortable at rest. Less than ordinary physical activity causes fatigue, palpitation, dyspnea or anginal pain
Class IV	Patients with cardiac disease resulting in inability to carry on any physical activity without discomfort. Symptoms of cardiac insufficiency or of the angina syndrome may be present even at rest. If any physical activity is undertaken, discomfort is increased.

Oxford textbook of internal medicine. Vol 2, pp 2228. Oxford University Press. 1997

Appendix 4
MDASI

M. D. Anderson Symptom Inventory (MDASI) Core Items

Part I. How **severe** are your symptoms?

People with cancer frequently have symptoms that are caused by their disease or by their treatment. We ask you to rate how severe the following symptoms have been ***in the last 24 hours.*** Please fill in the circle below from 0 (symptom has not been present) to 10 (the symptom was as bad as you can imagine it could be) for each item.

	Not Present 0	1	2	3	4	5	6	7	8	9	As Bad As You Can Imagine 10
1. Your **pain** at its WORST?	○	○	○	○	○	○	○	○	○	○	○
2. Your **fatigue (tiredness)** at its WORST?	○	○	○	○	○	○	○	○	○	○	○
3. Your **nausea** at its WORST?	○	○	○	○	○	○	○	○	○	○	○
4. Your **disturbed sleep** at its WORST?	○	○	○	○	○	○	○	○	○	○	○
5. Your feelings of being **distressed (upset)** at its WORST?	○	○	○	○	○	○	○	○	○	○	○
6. Your **shortness of breath** at its WORST?	○	○	○	○	○	○	○	○	○	○	○
7. Your problem with **remembering things** at its WORST?	○	○	○	○	○	○	○	○	○	○	○
8. Your problem with **lack of appetite**	○	○	○	○	○	○	○	○	○	○	○
9. Your feeling **drowsy (sleepy)** at its WORST?	○	○	○	○	○	○	○	○	○	○	○
10. Your having a **dry mouth** at its WORST?	○	○	○	○	○	○	○	○	○	○	○

	Not Present 0	1	2	3	4	5	6	7	8	9	As Bad As You Can Imagine 10
11. Your feeling **sad** at its WORST?											
12. Your **vomiting** at its WORST?	○	○	○	○	○	○	○	○	○	○	○
13. Your **numbness or tingling** at its WORST?											

Part II. How have your symptoms interfered with your life?

Symptoms frequently interfere with how we feel and function. How much have your symptoms interfered with the following items in the last 24 hours:

	Did Not Interfere 0	1	2	3	4	5	6	7	8	9	Interfered Completely 10
14. **General activity?**											
15. **Mood?**	○	○	○	○	○	○	○	○	○	○	○
16. **Work (including work around the house)?**											
17. **Relations with other people?**											
18. **Walking?**											
19. **Enjoyment of life?**											

Appendix 5
HCPEX

HCPEX-1

Healthcare Professionals' Experiences towards Either IV or SC Herceptin (VIAL) in Breast Cancer

(HCP Questionnaire)

At least 3 patients at your center have now finished the "in hospital" part of the trial. Your own experiences and opinions about the different methods are an important part of the study. Your confidentiality will be respected and none of the information we receive will be used in a way that could identify you.

Date: Day / Month / Year

Site Number: Staff Initials:

1. What is your specialty?

☐ oncologist ☐ gynaecologist ☐ oncologist/specialist chemo nurse ☐ other

If other please specify

Now some questions about the subcutaneous (SC) Herceptin administration:

2. Did you personally administer the SC Herceptin in the study?

☐ always ☐ sometimes ☐ never

If never who did administer the SC Herceptin in the study?

☐ another oncologist ☐ another gynaecologist ☐ another oncologist/specialist chemo nurse ☐ other

If other please specify

3. How many minutes preparation time was required for Herceptin SC vial in the pharmacy?

☐ <5 ☐ 6 -10 ☐ 11-15 ☐16-20 ☐ >20 ☐ not sure

4. How many minutes in total did it usually take to administer the Herceptin subcutaneously using the hand held syringe?

☐ <3 ☐ <5 ☐ 6 -15 ☐ 16-30 ☐ 31-60 ☐ 61-90 ☐ >90 ☐ not sure

5. How long do you think the SC sessions usually lasted from patients' arrival until departure?

☐< 2 hours ☐ > 2 but < 3 hours ☐ >3 but <4hours ☐ > 4 hours

6. How reliable was using the hand held syringe to give Herceptin subcutaneously?

☐ not at all ☐ fairly ☐ very

If not at all reliable please give details why:

7. Overall how easy did you/your staff find giving Herceptin subcutaneously using the hand held syringe?

☐ not at all ☐ fairly ☐ very

If not at all easy please give details why

Now some questions about the IV Herceptin administration:

8. Which IV method did **most** of your patients have for their Herceptin treatment during the study?

☐ cannula ☐ Venous Access Device (VAD) ☐ not sure

If VAD which type was this (tick all that apply):-

☐ Hickman ☐ Groshong ☐ PICC ☐ Port-a-Cath ☐ other ☐ not sure

9. Did you personally administer the IV Herceptin in this study?

☐ always ☐ sometimes ☐ never

If never, who did administer the IV Herceptin in the study?

☐ another oncologist ☐ another gynaecologist ☐ another oncologist/specialist
☐chemo nurse ☐ other ☐ not sure

If other please specify

10. Did you personally cannulate the patients having Herceptin in the study?

☐ always ☐ sometimes ☐ never

If never who did cannulate patients in the study?

☐ another oncologist ☐ another gynaecologist ☐ another oncologist/specialist chemo nurse ☐ other ☐ not sure

If other please specify

11. How many minutes preparation time do you think the Herceptin IV required in the pharmacy?

☐ <5 ☐ 6 -10 ☐ 11-15 ☐ 16-20 ☐ >20 ☐ not sure

12. If a patient needed cannulation how many minutes do you think this usually took?

☐ <5 ☐ 6 -10 ☐ 11-15 ☐ 16-20 ☐ >20 ☐ not sure

13. How many minutes in total do you think connecting the Herceptin infusion and administering it IV usually took?

☐ <3 ☐ <5 ☐ 6 -15 ☐ 16-30 ☐ 31-60 ☐ 61-90 ☐ >90 ☐ not sure

14. How long do you think the IV Herceptin sessions usually lasted from patients' arrival until departure?

☐ < 2 hours ☐ > 2 but < 3 hours ☐ >3 but <4hours ☐ > 4 hours

15. How anxious do you think the IV Herceptin treatment made patients feel?

☐ not at all ☐ fairly ☐ very

16. How reliable do you think the IV Herceptin administration was overall?

☐ not at all ☐ fairly ☐ very

If not at all reliable please give details why

17. Overall how easy did you/your staff find IV Herceptin administration

☐ not at all ☐ fairly ☐ very

Now consider using these different methods of Herceptin administration in normal practice outside the study setting:

Overall which method of administration would:
18. be quickest from start of preparation to finish of administration (excluding observation period)?

☐ IV ☐ SC (handheld syringe) ☐ no difference

19. require less resource use for preparation and administration, for example nursing time, facility costs, equipment etc?

☐ IV ☐ SC (handheld syringe) ☐ no difference

Thank you very much for your time

Appendix 6

PSQ1 & PSQ2

PSQ-1

Date :

Site Number : Patient Study Number:

Each question is about **your experience during the in hospital part of the study**
If a question does not apply to you, please use the ”Not applicable” option.

About the clinicians

1a Did the clinicians talk to you in a way that was easy to understand?							1b How important was this to you?				
Not at all	To a small extent	To a moderate extent	To a large extent	To a very large extent	Not applicable		Not important	A little important	Important	Very important	Of utmost importance
☐	☐	☐	☐	☐	☐		☐	☐	☐	☐	☐
2a Do you have confidence in the clinicians’ professional competence?							2b How important was this to you?				
Not at all	To a small extent	To a moderate extent	To a large extent	To a very large extent	Not applicable		Not important	A little important	Important	Very important	Of utmost importance
☐	☐	☐	☐	☐	☐		☐	☐	☐	☐	☐
3a To what degree do you perceive that the clinicians cared about you?							3b How important was this to you?				
Not at all	To a small extent	To a moderate extent	To a large extent	To a very large extent	Not applicable		Not important	A little important	Important	Very important	Of utmost importance
☐	☐	☐	☐	☐	☐		☐	☐	☐	☐	☐
4a Did you perceive the clinicians to be interested in your description of your situation?							4b How important was this to you?				

Not at all	To a small extent	To a moderate extent	To a large extent	To a very large extent	Not applicable		Not important	A little important	Important	Very important	Of utmost importance
☐	☐	☐	☐	☐	☐		☐	☐	☐	☐	☐
5a Did you get enough time to talk and interact with the clinicians?							5b How important was this to you?				
Not at all	To a small extent	To a moderate extent	To a large extent	To a very large extent	Not applicable		Not important	A little important	Important	Very important	Of utmost importance
☐	☐	☐	☐	☐	☐		☐	☐	☐	☐	☐

About the other staff

By the “other staff” we mean: the nursing staff

6a Did the nurses talk to you in a way that was easy to understand?							6b How important was this to you?				
Not at all	To a small extent	To a moderate extent	To a large extent	To a very large extent	Not applicable		Not important	A little important	Important	Very important	Of utmost importance
☐	☐	☐	☐	☐	☐		☐	☐	☐	☐	☐
7a Do you have confidence in the nurses’ professional skills?							7b How important was this to you?				
Not at all	To a small extent	To a moderate extent	To a large extent	To a very large extent	Not applicable		Not important	A little important	Important	Very important	Of utmost importance
☐	☐	☐	☐	☐	☐		☐	☐	☐	☐	☐
8a To what degree do you perceive that the nurses cared about you?							8b How important was this to you?				
Not at all	To a small extent	To a moderate extent	To a large extent	To a very large extent	Not applicable		Not important	A little important	Important	Very important	Of utmost importance
☐	☐	☐	☐	☐	☐		☐	☐	☐	☐	☐

9a Did you perceive the nurses to be interested in your description of your situation?							9b How important was this to you?				
Not at all	To a small extent	To a moderate extent	To a large extent	To a very large extent	Not applicable		Not important	A little important	Important	Very important	Of utmost importance
☐	☐	☐	☐	☐	☐		☐	☐	☐	☐	☐
10a Did you get enough time to talk and interact with the nurses?							10b How important was this to you?				
Not at all	To a small extent	To a moderate extent	To a large extent	To a very large extent	Not applicable		Not important	A little important	Important	Very important	Of utmost importance
☐	☐	☐	☐	☐	☐		☐	☐	☐	☐	☐

Other questions

11a Did you have to wait before you were admitted for services at the institution?					11b How important was this to you?				
No	Yes, but not long	Yes, quite long	Yes, much long		Not important	A little important	Important	Very important	Of utmost importance
☐	☐	☐	☐		☐	☐	☐	☐	☐

12a Did you get the impression that the hospital equipment was in good order?							12b How important was this to you?				
Not at all	To a small extent	To a moderate extent	To a large extent	To a very large extent	Not applicable		Not important	A little important	Important	Very important	Of utmost importance
☐	☐	☐	☐	☐	☐		☐	☐	☐	☐	☐
13a Did you get the impression that the hospital was otherwise in good order?							13b How important was this to you?				
Not at all	To a small extent	To a moderate extent	To a large extent	To a very large extent	Not applicable		Not important	A little important	Important	Very important	Of utmost importance
☐	☐	☐	☐	☐	☐		☐	☐	☐	☐	☐
14a Overall, was the help and treatment you received at the institution satisfactory?							14b How important was this to you?				
Not at all	To a small extent	To a moderate extent	To a large extent	To a very large extent	Not applicable		Not important	A little important	Important	Very important	Of utmost importance
☐	☐	☐	☐	☐	☐		☐	☐	☐	☐	☐

PSQ-2

Date :

Site Number : Patient Study Number:

Each question is about **your experience during the at home part of the study**
If a question does not apply to you, please use the ”Not applicable” option.

About the clinicians

1a Did the clinicians talk to you in a way that was easy to understand?							1b How important was this to you?				
Not at all	To a small extent	To a moderate extent	To a large extent	To a very large extent	Not applicable		Not important	A little important	Important	Very important	Of utmost importance
☐	☐	☐	☐	☐	☐		☐	☐	☐	☐	☐
2a Do you have confidence in the clinicians' professional competence?							2b How important was this to you?				
Not at all	To a small extent	To a moderate extent	To a large extent	To a very large extent	Not applicable		Not important	A little important	Important	Very important	Of utmost importance
☐	☐	☐	☐	☐	☐		☐	☐	☐	☐	☐
3a To what degree do you perceive that the clinicians cared about you?							3b How important was this to you?				
Not at all	To a small extent	To a moderate extent	To a large extent	To a very large extent	Not applicable		Not important	A little important	Important	Very important	Of utmost importance
☐	☐	☐	☐	☐	☐		☐	☐	☐	☐	☐
4a Did you perceive the clinicians to be interested in your description of your situation?							4b How important was this to you?				
Not at all	To a small extent	To a moderate extent	To a large extent	To a very large extent	Not applicable		Not important	A little important	Important	Very important	Of utmost importance
☐	☐	☐	☐	☐	☐		☐	☐	☐	☐	☐

5a Did you get enough time to talk and interact with the clinicians?							5b How important was this to you?				
Not at all	To a small extent	To a moderate extent	To a large extent	To a very large extent	Not applicable		Not important	A little important	Important	Very important	Of utmost importance
☐	☐	☐	☐	☐	☐		☐	☐	☐	☐	☐

About the other staff

By the “other staff” we mean: the nursing staff

6a Did the nurses talk to you in a way that was easy to understand?							6b How important was this to you?				
Not at all	To a small extent	To a moderate extent	To a large extent	To a very large extent	Not applicable		Not important	A little important	Important	Very important	Of utmost importance
☐	☐	☐	☐	☐	☐		☐	☐	☐	☐	☐
7a Do you have confidence in the nurses’ professional skills?							7b How important was this to you?				
Not at all	To a small extent	To a moderate extent	To a large extent	To a very large extent	Not applicable		Not important	A little important	Important	Very important	Of utmost importance
☐	☐	☐	☐	☐	☐		☐	☐	☐	☐	☐
8a To what degree do you perceive that the nurses cared about you?							8b How important was this to you?				
Not at all	To a small extent	To a moderate extent	To a large extent	To a very large extent	Not applicable		Not important	A little important	Important	Very important	Of utmost importance
☐	☐	☐	☐	☐	☐		☐	☐	☐	☐	☐
9a Did you perceive the nurses to be interested in your description of your situation?							9b How important was this to you?				
Not at all	To a small extent	To a moderate extent	To a large extent	To a very large extent	Not applicable		Not important	A little important	Important	Very important	Of utmost importance
☐	☐	☐	☐	☐	☐		☐	☐	☐	☐	☐

10a Did you get enough time to talk and interact with the nurses?							10b How important was this to you?				
Not at all	To a small extent	To a moderate extent	To a large extent	To a very large extent	Not applicable		Not important	A little important	Important	Very important	Of utmost importance
☐	☐	☐	☐	☐	☐		☐	☐	☐	☐	☐

Other questions

11a Did you get the impression that the nurses' equipment was in good order?							11b How important was this to you?				
Not at all	To a small extent	To a moderate extent	To a large extent	To a very large extent	Not applicable		Not important	A little important	Important	Very important	Of utmost importance
☐	☐	☐	☐	☐	☐		☐	☐	☐	☐	☐
12a Overall, was the help and treatment you received at home satisfactory?							12b How important was this to you?				
Not at all	To a small extent	To a moderate extent	To a large extent	To a very large extent	Not applicable		Not important	A little important	Important	Very important	Of utmost importance
☐	☐	☐	☐	☐	☐		☐	☐	☐	☐	☐
13a Overall, what benefit have you had from the care at home?							13b How important was this to you?				
Not at all	To a small extent	To a moderate extent	To a large extent	To a very large extent	Not applicable		Not important	A little important	Important	Very important	Of utmost importance
☐	☐	☐	☐	☐	☐		☐	☐	☐	☐	☐

Appendix 7
PEX-P1 & PEX-2

PEX – P1

Patient Experience with Herceptin IV and SC (vial) administered in the hospital

Date:

Site Number: **Patient Study Number:**

You have now received the drug Herceptin in 2 different ways in the hospital and I'd like to ask you a few questions about your experiences. There aren't any right or wrong answers. The interview should only take about 25 minutes. If you feel uncomfortable at any time please ask me to stop. None of the information will be used outside of the study in a way that could identify you.

First some general questions about the place where you had your study treatment

1. Where did you have your Herceptin study treatment?

☐ hospital chemotherapy department ☐ other chemotherapy clinic ☐ doctor's office ☐ other

2. Was this the same place as for your chemotherapy treatment?

☐ yes ☐ no

3. How long did it take you to travel there?

☐ <1 hour ☐ 1-2 hours ☐ >2 hrs

4. How easy was it travelling there?

☐ not at all ☐ fairly ☐ very

5. Did someone always have to take you there by car or public transport?

☐ always ☐ sometimes ☐ never

6. Was the cost of travelling there a problem for you?

☐ not at all ☐ fairly ☐ very

7. So taking all these things above into consideration was travelling for Herceptin treatment overall a problem for you?

☐ yes ☐ no

So that's the travelling now some questions about the place where you had your study treatment

8. How helpful were the nursing and medical staff who gave you your study treatment

☐ not at all ☐ fairly ☐ very

9. How pleasant overall was the place where you had your study treatment?

☐ not at all ☐ fairly ☐ very

Now I'll ask you about the 3 Herceptin study treatments which were given intravenously (Interviewer some explanation about different types of IV maybe needed please use your descriptions, photos and diagrams to help)

10. How was your IV Herceptin given?

☐ cannula/needle ☐ Venous Access Device(VAD) ☐ both

11. If VAD was this :- ☐ Hickman ☐ PICC ☐ Port-a-Cath ☐ other

Note to interviewer: If patient had VAD only then go to Q 15. If both methods of IV given (VAD and cannula) for any reason please ask Qs 12 to 25.

12. Did the hospital staff ever have any difficulty inserting the cannula (needle)?

☐ very often ☐ sometimes ☐ never

13. How many minutes did insertion of the cannula usually take?

☐ <5 ☐ 6 -10 ☐ 11-15 ☐ 16-20 ☐ >20

14. How painful was this usually?

☐ not at all ☐ fairly ☐ very

Note to interviewer if patient only ever had cannulation for IV go to Q 17

15. How many minutes did connection to the access port/line usually take?

☐ <5 ☐ 6 -10 ☐ 11-15 ☐ 16-20 ☐ >20

16. How painful was this usually?

☐ not at all ☐ fairly ☐ very

17. How long did the IV sessions usually last from arrival until departure?

☐ <2 hours ☐ >2 but <3 hours ☐ >3 but <4 hours ☐ >4hours

18. How anxious did having the IV treatment make you feel?

☐ not at all ☐ fairly ☐ very

19. In general how would you describe these IV Herceptin study sessions?

☐ very unpleasant ☐ somewhat unpleasant ☐ acceptable

Now I'd like to ask you about the 3 Herceptin study treatments which were given subcutaneously in the hospital

20. Did the medical or nursing staff ever have any difficulty giving the Herceptin injection subcutaneously?

☐ very often ☐ sometimes ☐ never

21. How many minutes did it usually take?

☐ <5 ☐ 6 -10 ☐ 11-15 ☐ 16-20 ☐ >20

22. How painful was this usually?

☐ not at all ☐ fairly ☐ very

23. How long did the SC sessions usually last from arrival in the hospital until departure ?

☐ <2 hours ☐ >2 but <3 hours ☐ >3 but <4 hours ☐ >4hours

24. How anxious did having the SC treatment make you feel?

☐ not at all ☐ fairly ☐ very

25. In general how would you describe these SC treatment sessions in the hospital?

☐ very unpleasant ☐ fairly unpleasant ☐ acceptable

Thank you very much for your time and help with this study. I hope that the rest of your treatment goes well.

PEX – P2

Patient experience with the at home administration of Herceptin subcutaneous vial

Date:

Site Number: **Patient Study Number:**

We would like to ask you some questions about your experiences with the at home administration of Herceptin subcutaneous vial. There aren't any right or wrong answers. The interview should only take about 5 minutes. If you feel uncomfortable at any time please ask me to stop. None of the information will be used outside of the study in a way that could identify you.

1. Did the nursing staff ever have any difficulty giving the Herceptin injection subcutaneously?

☐ very often ☐ sometimes ☐ never

2. How many minutes did the injection (it) usually take?

☐ <5 ☐ 6 -10 ☐ 11-15 ☐ 16-20 ☐ >20

3. How painful was this usually?

☐ not at all ☐ fairly ☐ very

4. How long did the SC sessions usually last from arrival until departure of the nurse ?

☐ <2 hours ☐ >2 but <3 hours ☐ >3 but <4 hours ☐ >4hours

5. How anxious did having the SC treatment make you feel?

☐ not at all ☐ fairly ☐ very

6. In general how would you describe these SC treatment sessions at home?

☐ very unpleasant ☐ fairly unpleasant ☐ acceptable

Thank you very much for your time and help with this study. I hope that the rest of your treatment goes well.
