# Supplementary material for: Safety and tolerability of subcutaneous trastuzumab at home administration, results of the phase IIIb open-label BELIS study in HER2-positive early breast cancer
Source: Breast Cancer Res Treat. 2020 Apr 2;181(1):97–105. doi: 10.1007/s10549-020-05604-7 (PMC7182624; doi:10.1007/s10549-020-05604-7)
Supplement: Supplementary file 2 — Supplementary file2 (PDF 55 kb) [file 10549_2020_5604_MOESM2_ESM.pdf]

	PATIENT	DATE	EVENT	GRADE	PERIOD	DAY OF ONSET IN TREATMENT PERIOD
1	Patient 260267004	31/03/2015	INJECTION SITE ERYTHEMA	GRADE1	3	DAY 22
	Patient 260267004	10/03/2015	INJECTION SITE SWELLING	GRADE1	3	DAY 1
	Patient 260267004	21/04/2015	INJECTION SITE SWELLING	GRADE1	3	DAY 43
	Patient 260267004	12/05/2015	INJECTION SITE SWELLING	GRADE1	3	DAY 64
	Patient 260267004	2/06/2015	INJECTION SITE SWELLING	GRADE1	3	DAY 85
	Patient 260267004	23/06/2015	INJECTION SITE SWELLING	GRADE1	3	DAY 106
2	Patient 260269001	22/07/2014	FATIGUE	GRADE 1	2	DAY 23
	Patient 260269001	1/09/2014	INJECTION SITE ERYTHEMA	GRADE1	3	DAY 1
	Patient 260269001	1/09/2014	INJECTION SITE SWELLING	GRADE1	3	DAY 1
	Patient 260269001	22/09/2014	INJECTION SITE SWELLING	GRADE1	3	DAY 22
	Patient 260269001	13/10/2014	INJECTION SITE SWELLING	GRADE1	3	DAY 43
	Patient 260269001	3/11/2014	INJECTION SITE SWELLING	GRADE1	3	DAY 64
	Patient 260269001	24/11/2014	INJECTION SITE SWELLING	GRADE1	3	DAY 85
	Patient 260269001	15/12/2014	INJECTION SITE SWELLING	GRADE1	3	DAY 106
3	Patient 260269002	1/12/2014	INJECTION SITE ERYTHEMA	GRADE1	2	DAY 43
	Patient 260269002	2014-12-UN	FATIGUE	GRADE 1	2	EXACT DAY UNKNOWN
4	Patient 260269003	2/02/2015	INJECTION SITE ERYTHEMA	GRADE1	3	DAY 22
	Patient 260269003	13/03/2015	FATIGUE	GRADE 2	3	DAY 63
5	Patient 260269004	24/02/2015	INJECTION SITE REACTION	GRADE 1	3	DAY 1
	Patient 260269004	17/03/2015	INJECTION SITE SWELLING	GRADE 1	3	DAY 22
	Patient 260269004	7/04/2015	INJECTION SITE SWELLING	GRADE 1	3	DAY 43
	Patient 260269004	28/04/2015	INJECTION SITE SWELLING	GRADE 1	3	DAY 64
	Patient 260269004	28/04/2015	FATIGUE	GRADE 1	3	DAY 64
6	Patient 260273003	5/08/2014	FATIGUE	GRADE 1	3	DAY 1
	Patient 260273003	26/08/2014	FATIGUE	GRADE 1	3	DAY 22
	Patient 260273003	7/10/2014	FATIGUE	GRADE 2	3	DAY 64
	Patient 260273003	28/10/2014	INJECTION SITE PAIN	GRADE 1	3	DAY 85
7	Patient 260284001	12/02/2014	FATIGUE	GRADE 1	1	DAY 35

	Patient 260284001	5/03/2014	FATIGUE	GRADE 2	1	DAY 56
	Patient 260284001	3/04/2014	INJECTION SITE ERYTHEMA	GRADE 1	2	DAY 22
	Patient 260284001	6/04/2014	INJECTION SITE ERYTHEMA	GRADE 1	2	DAY 25
	Patient 260284001	24/04/2014	INJECTION SITE ERYTHEMA	GRADE 1	2	DAY 43
	Patient 260284001	5/06/2014	INJECTION SITE ERYTHEMA	GRADE 1	3	DAY 22
	Patient 260284001	31/07/2014	INJECTION SITE ERYTHEMA	GRADE 1	3	DAY 78
	Patient 260284001	3/04/2014	INJECTION SITE PAIN	GRADE 1	2	DAY 22
8	Patient 260291001	19/02/2014	INJECTION SITE INFLAMMATI	GRADE 1	2	DAY 1
9	Patient 260291004	15/05/2014	FATIGUE	GRADE 1	1	DAY 1
	Patient 260291004	11/12/2014	INJECTION SITE INFLAMMATI	GRADE 1	3	DAY 85
	Patient 260291004	30/12/2014	INJECTION SITE SWELLING	GRADE 1	3	DAY 104
10	Patient 260292001	1/08/2014	INJECTION SITE PAIN	GRADE 1	3	DAY 85
	Patient 260292001	1/08/2014	INJECTION SITE SWELLING	GRADE 1	3	DAY 85
	Patient 260292001	2/08/2014	FATIGUE	GRADE 2	3	DAY 86
12	Patient 260292002	12/06/2014	INJECTION SITE ERYTHEMA	GRADE 1	3	DAY 1
	Patient 260292002	3/07/2014	INJECTION SITE ERYTHEMA	GRADE 1	3	DAY 22
	Patient 260292002	24/07/2014	INJECTION SITE ERYTHEMA	GRADE 1	3	DAY 43
	Patient 260292002	14/08/2014	INJECTION SITE ERYTHEMA	GRADE 1	3	DAY 64
	Patient 260292002	4/09/2014	INJECTION SITE ERYTHEMA	GRADE 1	3	DAY 85
	Patient 260292002	25/09/2014	INJECTION SITE ERYTHEMA	GRADE 1	3	DAY 106
	Patient 260292002	12/06/2014	INJECTION SITE SWELLING	GRADE 1	3	DAY 1
	Patient 260292002	24/07/2014	INJECTION SITE SWELLING	GRADE 1	3	DAY 43
	Patient 260292002	24/07/2014	FATIGUE	GRADE 2	3	DAY 43
	Patient 260292002	14/08/2014	INJECTION SITE SWELLING	GRADE 1	3	DAY 64
	Patient 260292002	4/09/2014	INJECTION SITE SWELLING	GRADE 1	3	DAY 85
13	Patient 260292004	29/09/2014	INJECTION SITE ERYTHEMA	GRADE 1	3	DAY 1
	Patient 260292004	20/10/2014	INJECTION SITE ERYTHEMA	GRADE 1	3	DAY 22
	Patient 260292004	10/11/2014	INJECTION SITE ERYTHEMA	GRADE 1	3	DAY 43
	Patient 260292004	22/12/2014	INJECTION SITE ERYTHEMA	GRADE 1	3	DAY 85
14	Patient 260411001	18/02/2014	INJECTION SITE ERYTHEMA	GRADE 1	2	DAY 22

15	Patient 260411004	28/01/2015	INJECTION SITE SWELLING	GRADE 1	3	DAY 85
16	Patient 260412001	25/04/2014	INJECTION SITE REACTION	GRADE 2	2	DAY 22
	Patient 260412001	18/07/2014	INJECTION SITE REACTION	GRADE 1	3	DAY 43
	Patient 260412001	8/08/2014	INJECTION SITE REACTION	GRADE 1	3	DAY 64
	Patient 260412001	8/08/2014	FATIGUE	GRADE 2	3	DAY 64
	Patient 260412001	1/10/2014	INJECTION SITE REACTION	GRADE 1	3	DAY 118
17	Patient 260412002	8/07/2014	INJECTION SITE ERYTHEMA	GRADE 1	3	DAY 1
	Patient 260412002	25/07/2014	INJECTION SITE ERYTHEMA	GRADE 1	3	DAY 18
	Patient 260412002	25/07/2014	FATIGUE	GRADE 1	3	DAY 18
	Patient 260412002	16/08/2014	INJECTION SITE ERYTHEMA	GRADE 1	3	DAY 40
	Patient 260412002	7/09/2014	INJECTION SITE ERYTHEMA	GRADE 1	3	DAY 62
	Patient 260412002	26/09/2014	INJECTION SITE ERYTHEMA	GRADE 1	3	DAY 81
	Patient 260412002	17/10/2014	INJECTION SITE ERYTHEMA	GRADE 1	3	DAY 102
18	Patient 260412003	14/11/2014	INJECTION SITE ERYTHEMA	GRADE 1	3	DAY 1
	Patient 260412003	5/12/2014	INJECTION SITE ERYTHEMA	GRADE 1	3	DAY 22
	Patient 260412003	26/12/2014	INJECTION SITE ERYTHEMA	GRADE 1	3	DAY 43
	Patient 260412003	16/01/2015	INJECTION SITE ERYTHEMA	GRADE 1	3	DAY 64
	Patient 260412003	6/02/2015	INJECTION SITE ERYTHEMA	GRADE 1	3	DAY 85
	Patient 260412003	27/02/2015	INJECTION SITE ERYTHEMA	GRADE 1	3	DAY 106
	Patient 260412003	14/11/2014	INJECTION SITE INDURATION	GRADE 1	3	DAY 1
	Patient 260412003	26/12/2014	INJECTION SITE INDURATION	GRADE 1	3	DAY 43
	Patient 260412003	16/01/2015	INJECTION SITE INDURATION	GRADE 1	3	DAY 64
	Patient 260412003	6/02/2015	INJECTION SITE INDURATION	GRADE 1	3	DAY 85
	Patient 260412003	5/12/2014	INJECTION SITE PAIN	GRADE 1	3	DAY 22
	Patient 260412003	5/12/2014	INJECTION SITE SWELLING	GRADE 1	3	DAY 22
	Patient 260412003	26/12/2014	INJECTION SITE SWELLING	GRADE 1	3	DAY 43
	Patient 260412003	5/12/2014	INJECTION SITE WARMTH	GRADE 1	3	DAY 22
	Patient 260412003	16/01/2015	INJECTION SITE WARMTH	GRADE 1	3	DAY 64
	Patient 260412003	2015-01-UN	FATIGUE	GRADE 1	3	EXACT DATE UNKNOWN
19	Patient 260414001	24/06/2014	INJECTION SITE ERYTHEMA	GRADE 1	3	DAY 2
	Patient 260414001	15/07/2014	INJECTION SITE ERYTHEMA	GRADE 1	3	DAY 22

	Patient 260414001	5/08/2014	INJECTION SITE ERYTHEMA	GRADE 1	3	DAY 43
	Patient 260414001	26/08/2014	INJECTION SITE ERYTHEMA	GRADE 1	3	DAY 64
	Patient 260414001	16/09/2014	INJECTION SITE ERYTHEMA	GRADE 1	3	DAY 85
	Patient 260414001	7/10/2014	INJECTION SITE ERYTHEMA	GRADE 1	3	DAY 106
	Patient 260414001	22/04/2014	INJECTION SITE INDURATION	GRADE 1	2	DAY 1
	Patient 260414001	13/05/2014	INJECTION SITE INDURATION	GRADE 1	2	DAY 22
	Patient 260414001	22/04/2014	INJECTION SITE PAIN	GRADE 1	2	DAY 1
	Patient 260414001	26/08/2014	INJECTION SITE SWELLING	GRADE 1	3	DAY 64
20	Patient 260414002	30/09/2014	INJECTION SITE ERYTHEMA	GRADE 1	3	DAY 1
	Patient 260414002	9/09/2014	INJECTION SITE SWELLING	GRADE 1	2	DAY 43
21	Patient 260414003	15/07/2014	FATIGUE	GRADE 2	1	DAY 22
	Patient 260414003	7/10/2014	INJECTION SITE SWELLING	GRADE 1	2	DAY 43
	Patient 260414003	28/10/2014	INJECTION SITE SWELLING	GRADE 1	3	DAY 1
	Patient 260414003	14/11/2014	INJECTION SITE SWELLING	GRADE 1	3	DAY 18
22	Patient 260414004	4/07/2014	FATIGUE	GRADE 1	1	DAY 22
	Patient 260414004	25/09/2014	FATIGUE	GRADE 3	2	DAY 43
	Patient 260414004	19/02/2015	INJECTION SITE SWELLING	GRADE 1	3	DAY 127
23	Patient 264555002	14/10/2014	INJECTION SITE HAEMATOMA	GRADE 1	3	DAY 23
	Patient 264555002	3/11/2014	INJECTION SITE HAEMATOMA	GRADE 1	3	DAY 43
	Patient 264555002	13/10/2014	INJECTION SITE PAIN	GRADE 1	3	DAY 22
	Patient 264555002	3/11/2014	INJECTION SITE PAIN	GRADE 1	3	DAY 43
	Patient 264555002	24/11/2014	INJECTION SITE PAIN	GRADE 1	3	DAY 64
	Patient 264555002	14/12/2014	INJECTION SITE PAIN	GRADE 1	3	DAY 84
24	Patient 264555003	4/01/2015	INJECTION SITE OEDEMA	GRADE 1	3	DAY 91
25	Patient 264555004	1/02/2015	INJECTION SITE HAEMATOMA	GRADE 1	3	DAY 85
	Patient 264555004	12/01/2015	INJECTION SITE PAIN	GRADE 2	3	DAY 65
26	Patient 264555006	10/12/2014	INJECTION SITE HAEMATOMA	GRADE 1	3	DAY 1
	Patient 264555006	10/12/2014	INJECTION SITE SWELLING	GRADE 1	3	DAY 1

27	Patient 264555007	19/10/2014	FATIGUE	GRADE 1	2	DAY 22
	Patient 264555007	30/11/2014	INJECTION SITE PAIN	GRADE 1	3	DAY 1
	Patient 264555007	21/12/2014	INJECTION SITE PAIN	GRADE 1	3	DAY 22
	Patient 264555007	12/01/2015	INJECTION SITE PAIN	GRADE 1	3	DAY 44
	Patient 264555007	1/02/2015	INJECTION SITE PAIN	GRADE 1	3	DAY 64
28	Patient 264556004	17/11/2014	INJECTION SITE ERYTHEMA	GRADE 1	2	DAY 1
	Patient 264556004	8/12/2014	INJECTION SITE ERYTHEMA	GRADE 1	2	DAY 22
	Patient 264556004	19/01/2015	INJECTION SITE ERYTHEMA	GRADE 1	3	DAY 1
29	Patient 264558001	7/10/2014	INJECTION SITE ERYTHEMA	GRADE 1	2	DAY 1
	Patient 264558001	10/02/2015	INJECTION SITE ERYTHEMA	GRADE 1	3	DAY 64
30	Patient 264559002	15/05/2014	INJECTION SITE PAIN	GRADE 1	2	DAY 1
31	Patient 264559003	17/07/2014	INJECTION SITE PAIN	GRADE 1	2	DAY 1
	Patient 264559003	17/07/2014	INJECTION SITE RASH	GRADE 1	2	DAY 1
	Patient 264559003	2014-10-UN	FATIGUE	GRADE 1	3	EXACT DATE UNKNOWN
32	Patient 264559004	19/10/2014	INJECTION SITE PAIN	GRADE 1	2	DAY 46
	Patient 264559004	29/01/2015	INJECTION SITE PAIN	GRADE 1	3	DAY 82
	Patient 264559004	18/02/2015	INJECTION SITE PAIN	GRADE 1	3	DAY 102
	Patient 264559004	4/09/2014	INJECTION SITE RASH	GRADE 1	2	DAY 1
	Patient 264559004	11/01/2015	FATIGUE	GRADE 1	3	DAY 64
	Patient 264559004	29/01/2015	INJECTION SITE RASH	GRADE 1	3	DAY 82
33	Patient 264560003	26/06/2014	INJECTION SITE PAIN	GRADE 1	2	DAY 44
34	Patient 264560006	17/09/2014	INJECTION SITE PAIN	GRADE 2	2	DAY 1
	Patient 264560006	12/10/2014	INJECTION SITE PAIN	GRADE 3	2	DAY 26
35	Patient 264560007	12/01/2015	INJECTION SITE PAIN	GRADE 2	2	DAY 2
	Patient 264560007	7/04/2015	INJECTION SITE SWELLING	GRADE 2	3	DAY 23
36	Patient 264560008	4/05/2015	INJECTION SITE PAIN	GRADE 2	3	DAY 22
	Patient 264560008	27/05/2015	INJECTION SITE PAIN	GRADE 2	3	DAY 45

37	Patient 264562005	1/02/2015	INJECTION SITE MACULE	GRADE 1	2	DAY 26
38	Patient 266042002	23/12/2014	INJECTION SITE INFLAMMATI(	GRADE 1	3	DAY 63
39	Patient 260271001	5/03/2014	FATIGUE	GRADE 1	2	DAY 43
40	Patient 260271003	18/12/2014	FATIGUE	GRADE 1	3	DAY 85
41	Patient 260284002	26/02/2014	FATIGUE	GRADE 2	1	DAY 1
42	Patient 260284004	30/12/2014	FATIGUE	GRADE 1	1	DAY 22
43	Patient 260291002	9/10/2014	FATIGUE	GRADE 1	3	DAY 1
44	Patient 260410001	12/05/2014	FATIGUE	GRADE 1	1	DAY 25
45	Patient 260411002	15/04/2014	FATIGUE	GRADE 1	2	DAY 22
46	Patient 260411003	13/05/2014	FATIGUE	GRADE 1	1	DAY 22
	Patient 260411003	3/06/2014	FATIGUE	GRADE 1	1	DAY 43
47	Patient 264554006	2015-03-UN	FATIGUE	GRADE 2	3	EXACT DATE UNKNOWN
48	Patient 264554007	25/01/2015	FATIGUE	GRADE 2	3	DAY 1
49	Patient 264554008	30/04/2015	FATIGUE	GRADE 2	3	DAY 134
50	Patient 264560004	1/05/2014	FATIGUE	GRADE 1	1	DAY 9
51	Patient 266042003	23/03/2015	FATIGUE	GRADE 1	3	DAY 65
52	Patient 267441001	2014-09-UN	FATIGUE	GRADE 1	3	EXACT DATE UNKNOWN
53	Patient 267441002	16/09/2014	FATIGUE	GRADE 2	3	DAY 37

54	Patient 267441003	2015-05-UN	FATIGUE	GRADE 1	3	EXACT DATE UNKNOWN
	Patient 267441003	8/09/2014	FATIGUE	GRADE 1	3	DAY 12
	Patient 267441003	31/10/2014	FATIGUE	GRADE 1	3	DAY 65
	Patient 267441003	21/11/2014	FATIGUE	GRADE 1	3	DAY 86
55	Patient 267441004	31/10/2014	FATIGUE	GRADE 1	3	DAY 1
